# Airborne contaminant exposure and bone development: a systematic review and meta-analysis

**DOI:** 10.1093/toxsci/kfag067

**Published:** 2026-06-02

**Authors:** Madeline K M Vera-Colón, Ruth M Meletz, Celina N Phillipson, Nicole R L Sparks

**Affiliations:** Environmental Health Sciences Graduate Program, University of California Irvine, Irvine, CA 92697, United States; Department of Occupational and Environmental Health, Joe C. Wen School of Population & Public Health, University of California Irvine, Irvine, CA 92697, United States; Environmental Health Sciences Graduate Program, University of California Irvine, Irvine, CA 92697, United States; Department of Occupational and Environmental Health, Joe C. Wen School of Population & Public Health, University of California Irvine, Irvine, CA 92697, United States; Environmental Health Sciences Graduate Program, University of California Irvine, Irvine, CA 92697, United States; Department of Occupational and Environmental Health, Joe C. Wen School of Population & Public Health, University of California Irvine, Irvine, CA 92697, United States; Environmental Health Sciences Graduate Program, University of California Irvine, Irvine, CA 92697, United States; Department of Occupational and Environmental Health, Joe C. Wen School of Population & Public Health, University of California Irvine, Irvine, CA 92697, United States

**Keywords:** air pollution, particulate matter, heavy metals, bone toxicology, bone development, systematic review, meta-analysis, oxidative stress

## Abstract

Air pollution, including particulate matter (PM10, PM2.5) and airborne metals (Cd, Hg, As, Pb) are a systemic health hazard, but osteotoxic effects remain underrecognized. We systematically synthesized human, animal, and in vitro evidence on airborne contaminants and outcomes to skeletal health. Following Office of Health Assessment and Translation (OHAT) guidance, we searched PubMed and Google Scholar (2014–2024; final search November 24, 2024) for English-language studies relating PM or airborne metals to skeletal outcomes, excluding studies limited to adult occupational exposures, age-related bone disease, or non-peer-reviewed reports. Studies were screened and data extracted in Covidence. Risk of bias was assessed using tools from the Quality Assessment and Risk of Bias Tool Repository, and certainty of evidence was graded with GRADE. We used a random-effects meta-analyses model to estimate relative risks (RRs) interpreted as directional consistency hazards for bone loss, impaired osteoblast differentiation, enhanced osteoclast activity, bone marrow adiposity, epigenetic changes, and altered bone microarchitecture. Fifty-three studies met the inclusion criteria. Across outcomes, meta-analyses showed higher hazard risk in exposed versus control groups, with RRs ranging from 7 to 10; for example, bone loss (RR 7.12, 95% CI 4.20–12.06) and altered bone microarchitecture (RR 10.28, 95% CI 4.67–22.61). Epidemiologic and experimental evidence may implicate oxidative stress, inflammation, and epigenetic dysregulation as key mechanisms. Overall certainty was low to moderate due to residual confounding, exposure misclassification, and heterogeneity. Growing evidence indicates that airborne PM and metals adversely affect bone health, particularly during critical developmental windows, underscoring the need to integrate skeletal endpoints into health assessment and policy.

## Graphical Abstract

**Figure:**
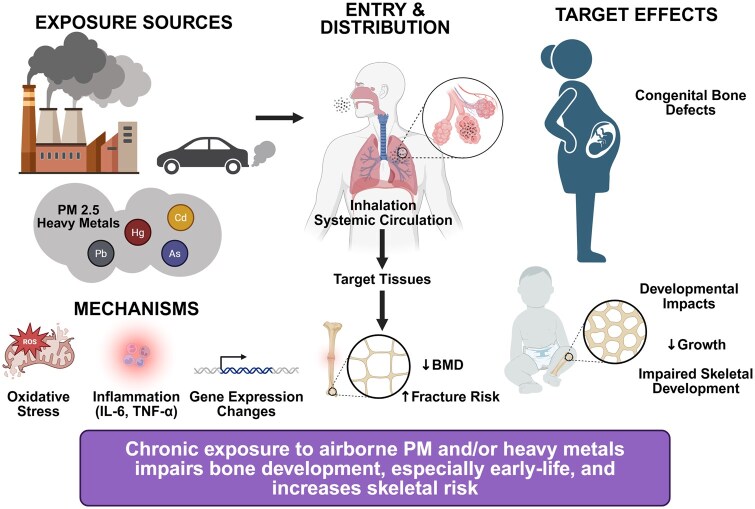

Air pollution is a complex and pervasive environmental hazard comprising a mixture of gaseous, particulate, and heavy metal pollutants (World Health Organization 2025). Particulate matter (PM) represents a large proportion of air pollution and comprises a heterogeneous mixture of constituents suspended in particles (Kim et al. 2015). PM is also one of six criteria pollutants identified by the United States (U.S.) Environmental Protection Agency (EPA), alongside carbon monoxide (CO), lead (Pb), nitrogen dioxide (NO_2_), ozone (O_3_), and sulfur oxides (SO_X_). Regulatory policies designed to define safe exposure levels to regulated ambient pollutants often rely on estimates based on similarly characterized pollutants, rather than precise, real-time assessments (EPA 2019). Of notable concern are particles smaller than 2.5 µm in aerodynamic diameter (PM2.5), capable of penetrating deep into the respiratory tract and absorbed through lower airway epithelial surfaces into systemic circulation (Bhattarai et al. 2020). In contrast, larger PM10 particles tend to deposit in the upper airways and are less likely to reach peripheral tissues (Abu-Elmagd et al. 2017). PM often includes harmful constituents such as heavy metals, including Pb, cadmium (Cd), arsenic (As), and mercury (Hg), whose toxicity is well-established (Tchounwou et al. 2012).

Existing reviews have begun to consolidate evidence linking air pollution exposure with bone-related outcomes. A 2021 systematic review focused on particle air pollution (primarily PM2.5 and PM10) and bone outcomes (bone health/osteoporosis risk/fractures) in human observational studies noted that inflammation and oxidative stress are plausible pathways connecting inhaled PM to skeletal remodeling (Pang et al. 2021). However, findings across studies were inconsistent, likely reflecting heterogeneity in subject characteristics, study design, and analytical approaches (Pang et al. 2021). More recently, a 2024 review in *Current Osteoporosis Reports* similarly describes a firm correlative link between poorer air quality and bone loss/fracture risk, while emphasizing the most damaging pollutant components; however, the precise mechanisms remain incompletely elucidated (Allen et al. 2024). Proposed pathways include systemic inflammatory signaling, oxidative damage in bone-relevant cells from pollutant constituents (including heavy metals), endocrine disruption, and vitamin D deficiency, cascades triggered by pollutant exposure (Allen et al. 2024). Complementing these narrative syntheses, a 2021 systematic review and meta-analysis integrating multiple criteria pollutants reported increased osteoporosis risk with exposure to several air pollutants (including PM2.5 and NO2), underscoring that bone endpoints are emerging as a measurable health outcome in air pollution epidemiology rather than only a downstream comorbidity (Liu et al. 2021). Finally, although most human evidence summarized in prior reviews has focused on older adults, there is increasing recognition that osteoporosis trajectories originate earlier in life and that early bone accrual and peak bone mass are critical determinants of later-life skeletal health, motivating greater attention to developmental susceptibility and early life exposure windows in newer work (Scheepers et al. 2025).

However, important gaps remain in the literature syntheses to date. First, existing reviews often emphasize epidemiologic associations and do not systematically integrate experimental toxicology evidence. For example, Pang et al. (2021) focus on human observational studies of particulate air pollution and osteoporosis-related outcomes and do not synthesize evidence from experimental models in a structured way. Second, skeletal outcomes are frequently framed as clinical comorbidities (e.g. osteoporosis or fracture risk) rather than evaluated explicitly as primary toxicological targets in a hazard-identification framework. Although Allen et al. (2024) highlight a correlative link between air pollution and osteoporosis risk and discuss the plausible inflammatory pathways, the most damaging pollutant components and mechanisms remain unresolved, and the evidence is not organized around hazard identification across evidence streams. Third, developmental windows receive relatively limited attention in these syntheses, with the emphasis largely on adult bone health endpoints rather than fetal, infant, or childhood skeletal development. Together, these limitations motivate an integrated weight-of-evidence (WoE) approach that jointly evaluates human observational and experimental toxicology data while explicitly considering developmental susceptibility.

Airborne PM not only induces airway irritation and inflammation but also acts as an aggregate vehicle for additional toxicants (e.g. heavy metals) to enter the deep lung space, creating additional exposure-related concerns. Heavy metals adsorbed onto PM can be inhaled and transported throughout the body, accumulating in organs, including bone (Lamas et al. 2023; Yang et al. 2023; Liu et al. 2025). Cadmium, for example, has been shown to impair osteoblast function, alter key bone development signaling pathways, and hinder bone mineralization, contributing to reduced bone mineral density (Wan et al. 2023). Chronic exposure to airborne metals is increasingly recognized as a contributor to skeletal disorders, including osteoporosis, fracture risk, and delayed bone development and turnover (Zhang et al. 2020b; Qi et al. 2023; Hamawandi et al. 2025; Duan et al. 2026). In the United States, the EPA sets National Ambient Air Quality Standards (NAAQS) to protect public health. The annual PM2.5 limits are 12 µg/m³ (primary) and 15 µg/m³ (secondary), with a 24-h standard of 35 µg/m³. For PM10, the 24-h limit is 150 µg/m³ (EPA 2019). Yet, multiple studies suggest that adverse health effects may occur even at levels below regulatory thresholds, particularly for vulnerable populations (Dadvand et al. 2013; Wu et al. 2016; Bhattarai et al. 2023; Liu et al. 2025). Indeed, EPA’s recent PM NAAQS review materials note that epidemiological studies evaluated in the 2019 Integrated Science Assessment (ISA) and 2022 ISA Supplement report associations with adverse health effects at PM2.5 concentrations below the then-current primary annual standard (EPA 2019a, 2022, 2024).

Beyond its structural role, bone is increasingly recognized as an endocrine organ that participates in systemic homeostasis through bone-derived signals and mineral metabolism regulation (Wei and Karsenty 2015; Karsenty 2023). This endocrine function provides biological plausibility for bone as both a direct and indirect target of air pollution. For instance, bone interacts with the central nervous system and stress/inflammatory signaling (bone–brain axis), in part through bone-derived hormones such as osteocalcin that act on the brain and influence neurodevelopment and cognition (Oury et al. 2013). Bone also interfaces with renal regulation of calcium/phosphate and vitamin D activation (bone–kidney axis) through tightly coupled mineral homeostasis pathway that underlie that “kidney disease-mineral and bone disorder (CKD-MBD)” framework (KDIGO 2017 Clinical Practice Guideline Update for the Diagnosis, Evaluation, Prevention, and Treatment of Chronic Kidney Disease–Mineral and Bone Disorder (CKD-MBD) 2017). During pregnancy, fetal skeletal mineralization requires substantial active placental calcium transfer, linking maternal mineral physiology, placental transport, and fetal bone accretion (Kovacs and Kronenberg 1997; Christopher and Kovacs 2016). Disruption of pulmonary and systemic inflammation, endocrine signaling, oxidative stress, and metal toxicokinetics may therefore influence skeletal development and remodeling not only through direct effects on osteoblasts/osteoclasts, but also through interconnected organ pathways that regulate mineral balance, growth, and developmental programming.

Although functional congenital anomalies tend to dominate research and clinical attention, structural and mechanistic anomalies, especially those affecting skeletal development, are difficult to diagnose and thereby research. In the United States, approximately 1 in 33 births (∼3%) are affected by congenital anomalies annually (MOD 2025). In California, birth defects accounted for about 1 in 4 infant deaths, underscoring that structural developmental abnormalities contribute substantially to early-life mortality and that survivors may face lifelong morbidity requiring ongoing medical care, making developmental skeletal endpoints a biologically and clinically important focus (MOD 2016, 2025). Although direct causal links between air pollution and skeletal birth defects remain understudied, the developmental period represents a sensitive window in which pollutant-related disruption of osteogenesis and mineralization could yield lasting effects on skeletal integrity across the life course. Consistent with this rationale, prior epidemiologic work has evaluated associations between ambient air pollution/traffic-related exposures and selected structural congenital anomalies, supporting continued investigation of air pollution as a developmental risk factor (Ritz 2010; Vrijheid et al. 2011; Padula et al. 2013).

Taken together, the literature links air pollution to increased risk of musculoskeletal disorders, fractures, and impaired bone growth in both developing and adult populations (Prada et al. 2020; Qu et al. 2025). Mechanistic studies further suggest that airborne heavy metals disrupt osteogenesis by impairing osteoblast differentiation, increasing osteoclast activity, and inducing oxidative stress (Prada et al. 2020; Palma et al. 2022; Ge et al. 2023; Yang et al. 2024). In this systematic review and meta-analysis, we address key limitations of prior syntheses by integrating epidemiologic and experimental evidence within a hazard-identification, WoE framework. Specifically, the scope of interest was guided by the question: “Does airborne contaminant exposure, specifically PM and heavy metals, impact bone-related health outcomes across the life course?”. Accordingly, we evaluate whether airborne particulate matter and heavy metal exposures constitute a potential hazard to bone health by (i) synthesizing human observational evidence alongside experimental toxicology findings, (ii) treating skeletal outcomes as primary endpoints rather than secondary comorbidities, and (iii) explicitly highlighting developmental susceptibility while not excluding adult data.

## Materials and methods

### Protocol

This systematic review followed the seven-step Office of Health Assessment and Translation (OHAT) framework (Rooney et al. 2014), including protocol development, study selection, data extraction, risk of bias assessment, confidence rating, evidence translation, and integration for hazard identification. The protocol was developed a priori, followed by two reviewers independently conducting screening and data extraction. The full protocol can also be found within the Supplementary Materials—Protocol. PRISMA 2020 checklist was also used for transparency (Supplementary Materials—PRISMA 2020 Checklist). This study was funded by NIEHS R00ES032486, and the review protocol was registered with PROSPERO, on December 1, 2025, (PROSPERO title registration: “Airborne contaminant exposure and bone development: A systematic review and meta-analysis”) to enhance transparency, reduce risk of selective reporting, and ensure methodological rigor through a priori specification of eligibility criteria and analytic methods.

The objective of our systematic review is to assess the impact of air pollution, including the specific constituents, heavy metals, and PM, on bone development. Air pollution is a major global health concern, however the mechanistic impact on bone health remains unclear. Existing studies report inconsistent associations between air pollution, as a mixture, and osteoporosis, generally an adult bone ailment, and no prior review has systematically synthesized this evidence. A rigorous synthesis is needed to inform risk assessment and policy.

### Search strategy and information sources

A broad search strategy (Table 1) was applied across PubMed and Google Scholar limited to studies published from 2014 to 2024. Broad searches with limiting the publication date of PubMed and Google Scholar still yielded many papers. Google Scholar results screened in relevance order and limited to the first 10 pages of results (∼100 papers: 10 per page/10 pages total), consistent with common practice due to decreasing topical relevance beyond early ranked results. These paper “hits” were put through a “first-pass” screening to determine field relevance. Titles and keywords were quickly reviewed to exclude irrelevant records (e.g. nonmedical topics, unrelated contaminants, theses, patents, and non-English papers) before import into Covidence for formal PECOS-based screening. No such truncation was applied to PubMed results, due to limited “hits.” Google Scholar was selected due to its transparency and lack of accessibility restrictions within the literature database. Additionally, we wanted to ensure the public had access to search for studies used within this review. Our study year range was selected to more closely reflect younger cohorts exposed to similar pollution signatures as today. However, in animal study papers that reference pre-existing work that is published before our cut-off, they were considered as supplemental references and in context to outcomes, but raw data were not included in analyses. To complement the explicit cutoffs on epidemiological evidence, the authors deemed most comparable to applying the same cutoffs across all studies included in this review, including experimental studies only from the last decade. Applying a consistent temporal boundary across study types minimized structural imbalances in the weight-of-evidence evaluation and prevented disproportionate influence of historically well-characterized toxicants relative to more recently studied exposure paradigms in the quantitative synthesis. The initial search was conducted on July 25, 2024, with the final search completed on November 24, 2024. PM (PM2.5 and 10) and heavy metals (Cd, Hg, As, Pb) were included due to their prevalence in air pollution and recognized systemic toxicity, including effects on the skeletal system (Tchounwou et al. 2012).

**Table 1.** Keyword searches for air pollution, PM, heavy metals, and bone defects/outcomes.

Database	Keywords	Identified	Included
PubMed	“air pollution,” “bone outcomes”	42	16
PubMed	“particulate matter,” “bone defects”	59	5
Google Scholar	“air pollution,” “bone outcomes”	79,200^a^	29
Google Scholar	“particulate matter,” “bone defects”	32,100^a^	18
PubMed	“arsenic,” “osteogenesis,” “mechanism”	9	3
PubMed	“arsenic,” “osteoporosis,” “mechanism”	3	2
PubMed	“lead,” “osteogenesis,” “mechanism”	10	2
PubMed	“lead,” “osteoporosis,” “mechanism”	5	2
PubMed	“cadmium,” “osteogenesis,” “mechanism”	13	10
PubMed	“cadmium,” “osteoporosis,” “mechanism”	46	8
PubMed	“mercury,” “osteogenesis,” “mechanism”	2	0
PubMed	“mercury,” “osteoporosis,” “mechanism”	2	0
Google Scholar	“arsenic”, “osteogenesis,” “mechanism”	1,570^a^	21
Google Scholar	“arsenic,” “osteoporosis,” “mechanism”	11,800^a^	15
Google Scholar	“Pb”, “osteogenesis,” “mechanism”	15,600^a^	3
Google Scholar	“Pb,” “osteoporosis,” “mechanism”	18,200^a^	22
Google Scholar	“cadmium,” “osteogenesis,” “mechanism”	2,940^a^	31
Google Scholar	“cadmium,” “osteoporosis,” “mechanism”	14,500^a^	31
Google Scholar	“mercury,” “osteogenesis,” “mechanism”	2,820^a^	1
Google Scholar	“mercury,” “osteoporosis,” “mechanism”	12,200^a^	7

a Only the first 100 paper (10 search pages; 10 articles per page) were viewed to determine field relevancy (via title and keywords).

### Inclusion and exclusion criteria and study selection

Tables 2 and 3 provide details on the inclusion and exclusion criteria of the population, exposure, comparator, outcome, and study type (PECOS). For both, eligible studies included exposure measurements evaluating the effects of airborne PM (PM2.5, PM10) or airborne heavy metals (Pb, Cd, Hg, As). Outcomes of interest included bone-related developmental and structural endpoints, which included congenital skeletal abnormalities, bone mineral density, bone morphology and/or architecture, and osteoporosis-like or low bone mass phenotypes attributable to exposure. Studies focused exclusively on age-related osteoporosis, osteopenia, or fractures resulting from trauma or nonosteoporotic causes were excluded. Only peer-reviewed studies published between 2014 and 2024 were included, as discussed in the section Search strategy and information sources. Review articles, abstracts, qualitative studies, and dissertations or theses were excluded, as were non-peer-reviewed publications, studies with licensing restrictions that prevented access, and articles published in languages other than English unless a journal-provided translation was available.

**Table 2.** PECOS summary of studies linking air pollution (PM, heavy metals) to bone outcomes in populations, specifically maternal, fetal, adolescence, and adults, for epidemiologic studies.

Decision	Population	Exposure	Comparator	Outcome	Study design	Paper parameters
Inclusion	Human^a^	Indoor or outdoor-specified airborne PM (PM2.5, PM10) and/or airborne metals (Pb, Cd, Hg, As)	Lower exposure, background exposure, or minimally exposed reference groups, as defined within study	Bone-related outcomes including congenital or developmental skeletal abnormalities, craniofacial defects, BMD, bone mineral content, bone histomorphometries and microarchitecture, osteoporosis or low bone mass with exposure-relevant inference	Cross-sectional, Case–control, Cohort,	English 2014–2024 UC access Open access Peer-reviewed
Exclusion	Occupational^b^	NA	NA	Outcomes limited to primary age-related osteoporosis, osteopenia, or fractures attributable to trauma or nonosteoporotic causes	Review articles, Abstracts, Qualitative studies Dissertations and theses reports	Published not in English^c^ Older than 10 yr Licensing prohibited Non-peer reviewed

PECOS (population, exposure, comparator, outcome, study type) of the association between air pollution exposure, specifically PM and heavy metals, and bone-relevant outcomes in developing systems. PM, particulate matter; BMD, bone mineral density.

a Human populations of any age provided the study evaluates airborne PM or metal exposures with relevance to bone development, remodeling, or biologically plausible developmental mechanisms (including prenatal, childhood, adolescence, or mechanistic inference from adult populations).

b Occupational studies excluded due to magnitude of exposure and exposure-timeframe (past developmental window).

c Unless directly translated by journal.

**Table 3.** PECOS summary of studies linking air pollution (PM, heavy metals) to bone outcomes in systems, specifically prenatal, fetal, adolescent, and adults, for toxicologic studies.

Decision	Population	Exposure	Comparator	Outcome	Study design	Paper parameters
Inclusion	In vivo experimental models^a^ in vitro experimental systems^b^	Indoor or outdoor-specified airborne PM (PM2.5, PM10) and/or airborne metals (Pb, Cd, Hg, As)	No exposure, vehicle controls, filtered air, or lower exposure conditions, as defined within individual studies	Bone-related outcomes including congenital or developmental skeletal abnormalities, craniofacial defects, BMD, bone mineral content, bone histomorphometries and microarchitecture, osteoporosis or low bone mass with exposure-relevant inference	Randomized control vs treatment experimental designs	English 2014–2024 UC access Open access Peer-reviewed
Exclusion	in vitro systems irrelevant to bone development and formation	NA	NA	Outcomes limited to primary age-related osteoporosis, osteopenia, or fractures attributable to trauma or nonosteoporotic causes	Review articles, Abstracts, Qualitative studies Dissertations and theses reports	Published not in English^c^ Licensing prohibited Non-peer reviewed

PECOS (population, exposure, comparator, outcome, study type) of the association between air pollution exposure, specifically PM and heavy metals, and bone-relevant outcomes in developing systems. PM, particulate matter; BMD, bone mineral density.

a Animal model systems (e.g. mice, rats, duck, chicken).

b Bone-relevant mammalian cell types only (HSCs, BM-EPCs, BM-MSCs [human/rat/mice], hFOB1.19, ROS728, MC-3T3-E, Saos-2, RAW264.7, human primary osteoblasts).

c Unless directly translated by journal.

Studies meeting PECOS-based eligibility criteria were stratified by contaminant class (PM vs heavy metals) and developmental stage context (humans, in vivo rodent models, and in vitro experiments focused on developing systems) for analysis, as outlined in Tables 2 and 3. For synthesis, eligible studies were then grouped by bone-related outcome domain (e.g. bone loss, impaired osteoblast differentiation, altered microarchitecture, epigenetic changes, enhanced osteoclast activity, bone marrow adiposity), and these outcome-specific groups formed the basis for both narrative summaries and pooled relative risk meta-analyses interpreted as directional consistency hazards. Quantitative pooling was conducted within outcome domains; epidemiologic estimates were synthesized narratively unless compatible data permitted reconstruction.

#### Epidemiological inclusion and exclusion criteria and study selection

For epidemiologic studies (Table 2), eligible study designs included cross-sectional, case-control, and cohort studies conducted in human populations of any age, provided that airborne PM or metal exposures were relevant to bone development, remodeling, or biologically plausible exposure-related mechanisms. Occupational studies were excluded to maintain focus on ambient and environmentally relevant airborne exposures. Evidence from environmental and occupational epidemiology studies demonstrates that exposure timing relative to biologically susceptible windows can influence observed associations and interpretation of effect estimates (Steenland et al. 2018; Arroyave et al. 2021). Because occupational airborne exposures occur at higher intensities and during distinct life stages compared with community-level ambient exposures, inclusion of occupational cohorts could introduce heterogeneity in exposure magnitude, timing, and co-exposure profiles. Restricting inclusion to nonoccupational populations allowed for a more consistent evaluation of environmentally relevant exposure contexts across epidemiologic and experimental evidence (Whitney et al. 2021).

#### Experimental inclusion and exclusion criteria and study selection

For experimental studies (Table 3), including in vivo experimental model systems (e.g. rodent (mouse, rat), duck, chicken), or bone-relevant in vitro mammalian cell types (e.g. HSCs, BM-EPCs, BM-MSCs (human, mouse, rat), hFOB1.19, ROS728, MC-3T3-E, Saos-2, RAW264.7, human primary osteoblasts). Comparators consisted of no-exposure conditions, vehicle controls, filtered air, or lower-exposure groups as defined in individual studies.

### Data extraction

#### Epidemiological data extraction

Data on the study characteristics, population, outcomes, statistical methods, and other relevant findings were extracted after full-text screening. Epidemiological extractions included hazard ratios, identified means from full models, means, odds ratios, and 95% confidence intervals (Table S1). We extracted detailed study-level data, including country, design, sample size, age and sex distribution, exposure metrics and averaging times, follow-up duration, adjustment sets, and coded bone-related outcomes into six a priori domains (bone loss, impaired osteoblast differentiation, bone marrow adiposity, epigenetic changes, enhanced osteoclast activity, and altered bone microarchitecture). When studies reported multiple exposure metrics, time points, or outcome measures, we preferentially used the most fully adjusted estimate at the longest follow-up for meta-analysis, retaining other estimates for narrative synthesis only, and excluded studies with missing or unclear data from quantitative pooling when compatible effect estimates or 2 × 2 tables could not be derived for meta-analysis.

#### Experimental data extraction

Experimental extractions included adjusted *P*-value from sequencing runs, bone measurements (bone mineral density), staining and assay quantification (OCN; ALP; MTT), and fold change gene expression (Supplementary Materials—Extraction). From extraction details, we coded bone-related outcomes into six a priori domains (bone loss, impaired osteoblast differentiation, bone marrow adiposity, epigenetic changes, enhanced osteoclast activity, and altered bone microarchitecture). We utilized experimental sample sizes and mean values, when significance was reported, to construct 2×2 tables to calculate risk ratios in the context of bone-related outcomes.

### GRADE and risk of bias assessment

The Grading of Recommendations Assessment, Development, and Evaluation (GRADE) framework was utilized to assess the quality of evidence and the strength of recommendations in healthcare and clinical guidelines. It is widely adopted by organizations like the WHO, Cochrane, and many clinical practice guideline panels. Risk of bias was assessed using the Quality Assessment and Risk of Bias Tool Repository (QAROBSTR) (Open Science Foundation 2021) and visualized with the Risk of Bias Visualization Tool (robvis) (RiskofBias.info 2025), which helped generate the overall risk of bias. One reviewer performed the risk of basis assessment. Assessments followed predefined criteria to ensure consistency. No automated tools were used to generate these ratings; robvis was applied only to visualize the manually entered risk-of-bias assessments in tabular and graphical form (Table S2).

We did not perform funnel plots, Egger’s regression, or related small-study bias tests. Most outcome groups contained *k* < 10 studies and combined heterogeneous designs (in vivo, in vitro, epidemiological), violating assumptions required for stable asymmetry tests and limiting interpretability. Zero-event studies and sparse data further distort variance–effect relationships that these tests exploit. Accordingly, we addressed potential reporting bias narratively and within GRADE (publication bias/imprecision domains), and we note this limitation explicitly in the *Discussion*.

### Calculated relative risk ratio meta-analysis interpreted as directional consistency hazards

A relative risk assessment was performed across six common bone-related outcomes identified from extracted studies (Bone Loss, Impaired Osteoblast Differentiation, Bone Marrow Adiposity, Epigenetic Changes, Enhanced Osteoclast Activity, and Altered Bone Microarchitecture). For each health outcome, 2×2 tables were constructed, where (a) = exposure + outcome, (b) = exposure + no outcome, (c) = no exposure + outcome, and (d) = no exposure + no outcome. Study effects were computed as a log risk ratio (RR): $RR=\log(\frac{\frac{a}{a+b}}{\frac{c}{c+d}})$, with a large sample variance $v=\frac{1}{a}-\frac{1}{a+b}+\frac{1}{c}-\frac{1}{c+d}$. For sparse-cell handling, a treatment-arm continuity correction was applied to single-zero event arms: If (a = 0) or (c = 0) a 0.5 was added to that event cell only. If both events were 0 after correction, the study was excluded from pooling.

We recalculated unadjusted relative risks (RRs) from reconstructed 2×2 tables because the effect measures reported in individual studies (e.g. adjusted hazard ratios/odds ratio/RRs) were not methodologically comparable for pooling due to differences in exposure scaling/metrics, averaging windows, model specification, and covariate adjustment. Because 2×2 tables do not control for confounding, these unadjusted pooled RRs are not interpreted as adjusted causal estimates in humans; rather, they are used to harmonize binary exposed-versus-control contrasts and summarize concordant directionality of adverse effects across biological systems within a hazard-identification framework. We summed each cell corresponding to (a), (b), (c), and (d) into a final cumulative 2×2 containing all studies identified as having significant outcomes for each health effect. These cumulative cell counts are presented descriptively to summarize total event distributions across studies. However, pooled effect estimates were derived from study-level log risk ratios using inverse-variance random-effects weighting and not from aggregated cell counts. Adjusted hazard ratios and odds ratios from epidemiologic studies were extracted and reported separately (Table S1) but were not pooled due to the reasons above, which precluded methodologically comparable quantitative synthesis across study designs.

For the random-effects meta-analysis, study-level log risk ratios were pooled under a random-effects (RE) model using DerSimonian–Laird for between-study variance:

${Ƭ}_{DL}^{2}=\;\max\{0,\frac{Q-(k-1)}{\sum w_{i}-\frac{\sum w_{i}^{2}}{w_{i}}}\}$ , where $w_{i}=\;\frac{1}{v}$. Random-effects weight was measured, $w_{i}^{*}=\;\frac{1}{v+{Ƭ}_{DL}^{2}}$ and pooled log-RR and standard error was calculated, ${\hat{\theta }}_{RE}=\;\frac{\sum w_{i}^{*}y_{i}}{\sum w_{i}^{*}}$, $SE{(\hat{\theta }}_{RE})=\;\sqrt{\frac{1}{\sum w_{i}^{*}}}$. Alongside of RR, 95% confidence intervals (CI) were calculated: ${\hat{\theta }}_{RE}\;\pm 1.96\sqrt{{{Ƭ}_{DL}^{2}+SE({\hat{\theta }}_{RE})}^{2}}.$

For heterogeneity, between-study heterogeneity was determined by calculating Cochran’s *Q* and *I*^2^ using inverse-variance fixed-effect weights $Q=\sum w_{i}{(y_{i}-{\hat{\theta }}_{FE)}}^{2},$  ${\hat{\theta }}_{FE}=\frac{\sum w_{i}y_{i}}{\sum w_{i}}$, $I^{2}=\max\left(0,\;\frac{Q-(k-1)}{Q}\right)*100$. We also report $\tau_{\text{DL}}^{2}$from the random-effects model. We did not prespecify or perform subgroup analyses or meta-regression, given uniformly low heterogeneity across most outcome groups (nonsignificant *Q*, *I*^2^≈0%, τ^2^≈0) and small numbers of studies per potential subgroup, which would make such analyses underpowered and potentially misleading. Given heterogeneity in exposure metrics, biological systems, and outcome definitions, pooled RRs should be interpreted as indicators of directional consistency of adverse effects rather than quantitative human risk estimates.

We selected a random-effects model a priori because true effects were expected to vary across exposure types, biological systems, and outcome methods; we used the DerSimonian–Laird estimator to quantify between-study variance (reporting *Q*, $I^{2}$, and $\tau_{\text{DL}}^{2}$) and include prediction intervals to reflect the anticipated dispersion of effects.

We did not conduct additional sensitivity analyses because heterogeneity was negligible for most outcomes (*I*^2^ ≈ 0% in 4/6 outcome groups, nonsignificant *Q*, τ^2^ ≈ 0), and effect directions were uniform across studies even under sparse-data continuity correction, making fixed- and random-effects estimates essentially identical. Given the small study counts in several subgroups, further leave-one-out or stratified analyses would be underpowered and unlikely to change the conclusions; therefore, we report the prespecified DL random-effects results with full heterogeneity metrics.

For plotting, we used the natural log scale so that the no-effect line is ln(RR) = 0 and interpreted significance by whether the 95% CI crossed RR = 1. All meta-analysis functions were performed on Python3 via Jupyter notebook. Codes are available at https://github.com/mveracolon/Bone-Development-Air-Pollution.git. Data was visualized using a forest plot to observe RR and 95% CI with heterogeneity metrics included.

## Results

Following the systematic review protocol, we identified and evaluated studies characterizing the relationship between airborne contaminant exposure and bone health across epidemiological and experimental models. To align with toxicological and environmental health frameworks, findings were organized by contaminant class, PM, and heavy metals. Evidence from human, animal, and in vitro studies was integrated, emphasizing developmental and structural outcomes.

### Study selection

**Fig. 1.**
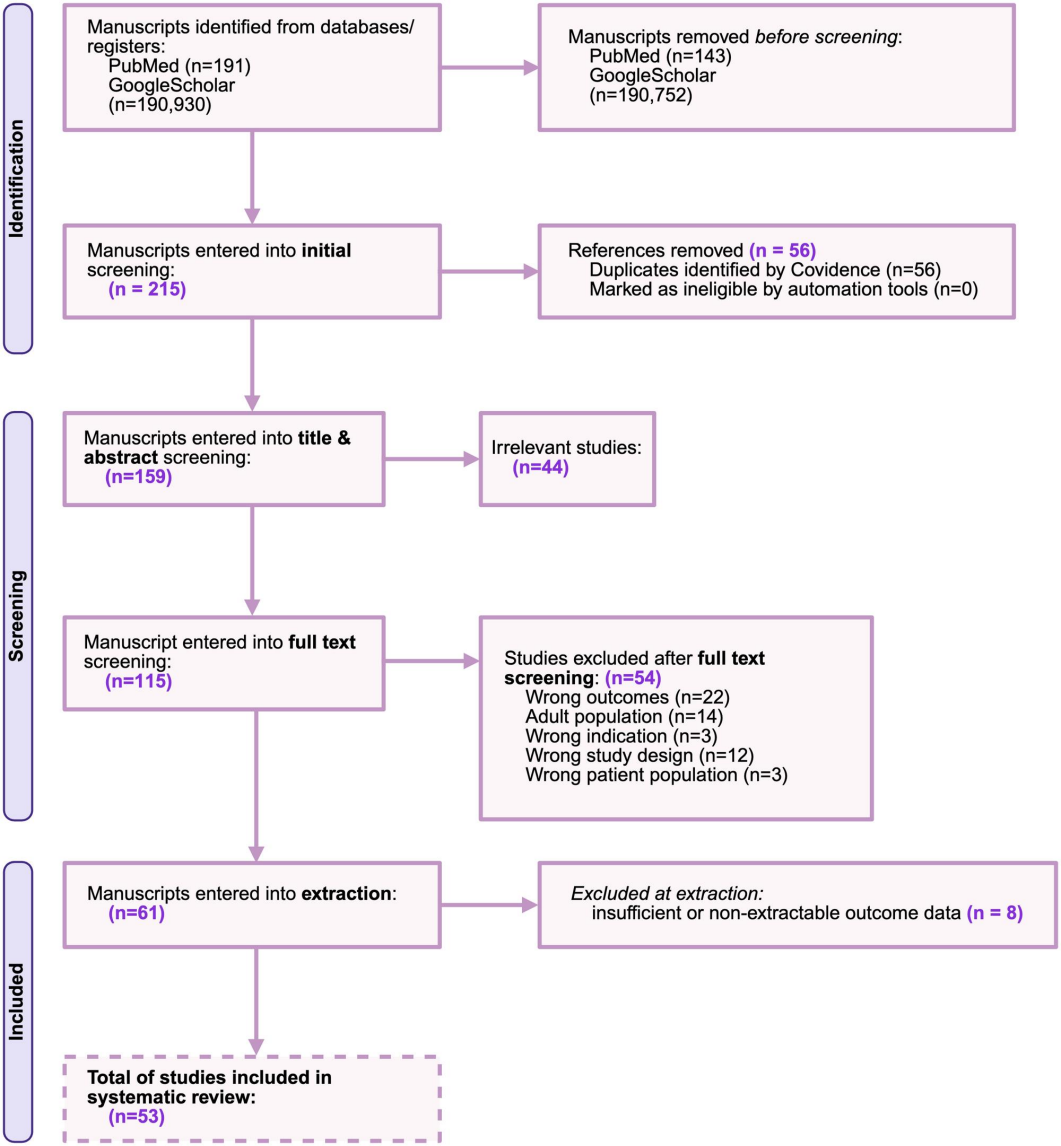
PRISMA flow diagram depicting the study selection process for the systematic review. The numbers excluded prior to manual screening reflect an initial relevance filter applied to all database search engine results (including an additional restriction to Google Scholar to the first 10 pages ∼100 articles (10 manuscripts/10 search pages) before importing citations into Covidence). The diagram outlines the number of manuscripts identified, screened, and assessed for eligibility, along with reasons for exclusion at each stage. Of the 215 manuscripts initially screened, 53 met all inclusion criteria and were included in the final analysis.

As described in the section *Search strategy and information sources*, a broad search strategy was implemented before import into Covidence. This initial field relevance screening (e.g. environmental studies with no biological exploration, other pollutants described, field relevance) resulted in 190,815 articles identified as irrelevant to the review question, and 215 total articles were uploaded onto Covidence. During import, 56 articles were automatically identified and removed by Covidence software as duplicates. The remaining 158 articles underwent initial title and abstract screening, which resulted in the removal of 44 articles as irrelevant. A total of 115 articles were entered into the full-text screening. Of these, 54 articles were excluded due to wrong outcomes (*n* = 22), adult population (*n* = 14), wrong indication (*n* = 3), wrong study design (*n* = 12), or wrong patient population (*n* = 3). 61 articles were included in the data extraction phase; however, 8 additional articles were removed as there was insufficient data for extraction (Fig. 1). A final article counted 53 and proceeded to data extraction.

Studies were separated into PM (*n* = 10) or heavy metal (*n* = 43), then further separated by study design (e.g. epidemiologic, experimental). The selected studies encompassed diverse cellular and bone health outcomes linked to exposure, including bone loss, impaired osteoblast differentiation, bone marrow adiposity, epigenetic changes, enhanced osteoclast activity, and altered bone microarchitecture.

### Study characteristics

Exposures examined included PM10 (*n* = 6), PM2.5 (*n* = 10), As (*n* = 5), Cd (*n* = 24), Hg (*n* = 3), and Pb (*n* = 7). The results sections Relative risk ratio meta-analysis interpreted as directional consistency hazard and Exposure to air pollution and/or PM expand on the included studies and describe the health outcomes that impact bone both directly and indirectly.

#### Epidemiological data study characteristics

For epidemiological studies, studies included developmentally sensitive cohorts to reduce bone health outcomes that can be attributed to aging, such as osteoporosis.

#### Experimental data study characteristics

Experimental work model included animal model systems (mice, rats, duck, chicken) and cell line systems (HSCs, BM-EPCs, BM-MSCs (human/rat/mice), hFOB1.19, ROS728, MC-3T3-E, Saos-2, RAW264.7, human primary osteoblasts).

### GRADE summary

Table 4 summarizes GRADE evidence on airborne contaminants and bone-related outcomes. Moderate-certainty evidence links long-term PM2.5 and PM10 exposure to higher risks of musculoskeletal disorders, fractures, and fetal growth restriction, with maternal exposure during pregnancy associated with impaired skeletal development. Experimental studies provide low-to-moderate certainty that Pb, Cd, and other heavy metals disrupt osteoblast differentiation, bone microarchitecture, and osteoclast activity through oxidative stress. Despite model limitations, consistent pathway disruptions support the plausibility of these clinical associations.

**Table 4:** .

Outcome	No. of studies	Study design	Summary of findings	Overall certainty
Bone loss from heavy metal exposure	**6 human studies** (Lim et al. 2016; Taha et al. 2018; Buha et al. 2019; Zeng et al. 2019; Tang et al. 2022; Wang et al. 2022b) **16 in vivo studies** (Duranova et al. 2014; Wu et al. 2014; Brzóska et al. 2016; Liu et al. 2016; Tomaszewska et al. 2016; Rafiei et al. 2018; Liu et al. 2019; Pamphlett et al. 2019; Zhang et al. 2020b; Luo et al. 2021; Ma et al. 2021a, 2021b; Chen et al. 2022; Torres-Rodriguez et al. 2022; Song et al. 2023; Hu et al. 2023) **7 in vitro studies** (Xu et al. 2014; Zhao et al. 2014; He et al. 2019; Liu et al. 2020; Tong et al. 2023; Wan et al. 2023; Zhou et al. 2023)	Experimental and Observational	Chronic Cd exposure caused trabecular thinning, cortical porosity, and decreased BMD; histological and architectural damage in compact bone; in vivo bone loss and inflammation	Low to Moderate
Impaired osteoblast differentiation	**1 human study** (Zeng et al. 2019) **8 in vivo studies** (Wu et al. 2014; Abnosi et al. 2017; Beier et al. 2017; Liao et al. 2017; Rafiei et al. 2018; Ma et al. 2021a, 2021b; Ran et al. 2023) **12 in vitro studies** (Zhao et al. 2014; Papa et al. 2015; Al-Ghafari et al. 2019; He et al. 2019; Lv et al. 2019; Wu et al. 2019; Liu et al. 2020; Ou et al. 2021; Wang et al. 2022a, 2022b; Zhou et al. 2023; Xu et al. 2024)	Experimental and Observational	Cd suppressed ALP, OCN, Runx2, BMP-2, and Wnt-related genes; Reduced calcium deposition and ALP activity in MSCs; Dose-dependent inhibition of osteoblast gene expression	Low
Altered bone microarchitecture	**1 human study** (Tong et al. 2022) **11 in vivo studies** (Duranova et al. 2014; Wu et al. 2014; Brzóska et al. 2016; Liu et al. 2016; Tomaszewska et al. 2016; Zhang et al. 2020b; Luo et al. 2021; Ma et al. 2021a, 2021b; Chen et al. 2022; Torres-Rodriguez et al. 2022) **3 in vitro studies** (Liu et al. 2020; Song et al. 2023; Wan et al. 2023)	Experimental and Observational	Histology and micro-CT showed reduced trabecular number, cortical thinning, porosity, and disrupted architecture	Low
Enhanced osteoclast activity	**5 in vivo studies** (Liu et al. 2016; Zhang et al. 2020b; Ma et al. 2021a, 2021b; Chen et al. 2022) **7 in vitro studies** (He et al. 2019; Lv et al. 2019; Liu et al. 2020; Wang et al. 2022a, 2022b; Zhou et al. 2023)	Experimental	Cd promoted osteoclast differentiation via RANKL/NFATc1; Increased osteoclastogenesis in BMMs and elevated TRAP and CTSK expression	Low
Oxidative stress and Nrf2 suppression	**1 human study** (Tong et al. 2022) **5 in vivo studies** (Abnosi et al. 2017; Buha et al. 2019; Liu et al. 2019; Zhang et al. 2020b; Luo et al. 2021) **3 in vitro studies** (Zhao et al. 2014; Al-Ghafari et al. 2019; Zhou et al. 2023)	Experimental and Observational	Cd caused elevated ROS and decreased antioxidant enzymes; Suppressed antioxidant defense and lipid peroxidation	Low
Disruption of Wnt/β-catenin signaling	**1 human study** (Zeng et al. 2019) **5 in vivo studies** (Wu et al. 2014; Beier et al. 2015, 2017; Abnosi et al. 2017; Ran et al. 2023) **2 in vitro studies** (Papa et al. 2015; Zhou et al. 2023)	Experimental and Observational	Downregulated Wnt3a and β-catenin; Disrupted calcium signaling and ALP activity	Low
Epigenetic changes and miRNA dysregulation	**1 human study** Chen et al. 2014) **3 in vivo studies** (Rafiei et al. 2018; Li et al. 2021; Ma et al. 2021a) **4 in vitro studies** (Zhao et al. 2014; Wu et al. 2019, 2020; Wang et al. 2022a)	Experimental and Observational	Altered miRNA expression that repressed osteogenic pathways	Low
Molecular effects on bone-related cells	**2 in vivo studies** (Li et al. 2021; Bhattarai et al. 2023) **2 in vitro studies** (Abu-Elmagd et al. 2017; Al-Ghafari et al. 2019)	Experimental	PM altered gene/miRNA expression, inflammatory markers, calcium pathways, oxidative stress, and impaired stem cell function	Low to Very Low
Bone marrow adiposity	**3 in vivo studies** (Beier et al. 2015; Abnosi et al. 2017; Luo et al. 2021) **1 in vitro study** (Zhao et al. 2014)	Experimental	Suppression of osteogenesis and Wnt signaling; Senescence-induced adipogenesis	Low
Musculoskeletal disorders	**2 human studies** (Qi et al. 2023; Cheng et al. 2024)	Observational	PM2.5/10 exposure associated with higher risk of musculoskeletal disorders; Associations remain after full adjustment	Moderate
Skeletal effects of early life exposure	**2 in vivo studies** (Beier et al. 2015; Bhattarai et al. 2023)	Experimental	Altered skeletal morphology, increased marrow adiposity, disrupted growth plate, and reduced osteogenesis-related genes	Low
Bone formation in mice	**2 in vivo studies** (Pamphlett et al. 2019; Ge et al. 2023)	Experimental	PM2.5 disrupted osteoblast maturation and bone formation; PM exposure disrupted architecture in juvenile mice	Low
Congenital malformations	**1 human study** (Farhi et al. 2014)	Observational	PM10 exposure associated with increased risk of congenital anomalies	Moderate
Fracture risk	**1 human study** (Qi et al. 2023)	Observational	Strong association between PM2.5 and fracture risk; Consistent across models and pollutants	Moderate
Bone turnover in children	**1 human study** (Liu et al. 2015)	Observational	PM10 associated with increased bone turnover markers in children	Low
Fetal bone growth	**1 human study** (Cao et al. 2019)	Observational	Maternal PM2.5 exposure during pregnancy associated with significant reductions in fetal growth parameters	Moderate
Placental epigenetic changes	**1 human study** (Maghbooli et al. 2018)	Observational	PM exposure linked to increased global DNA methylation in placental tissue	Low

The strength of evidence does vary. The strongest biological findings, from this subset of metal exposure that concern Cd-related suppression of osteogenic markers and bone remodeling defects, are from experimental systems (cell and animal models) spanning developmental and adult exposures, whereas human evidence remains limited (but suggests associations) particularly for mechanistic endpoints. Moderate evidence links bone loss to Wnt/β-catenin disruption, though variability in study design and limited human data reduce certainty. Other outcomes, such as marrow adiposity, skeletal malformations, and epigenetic changes, remain inconclusive, often based on isolated or underpowered studies. Preliminary observations, including organoid bone formation, placental epigenetic changes, and fetal growth effects, require replication and functional validation.

### Relative risk ratio meta-analysis interpreted as directional consistency hazard

**Fig. 2.**
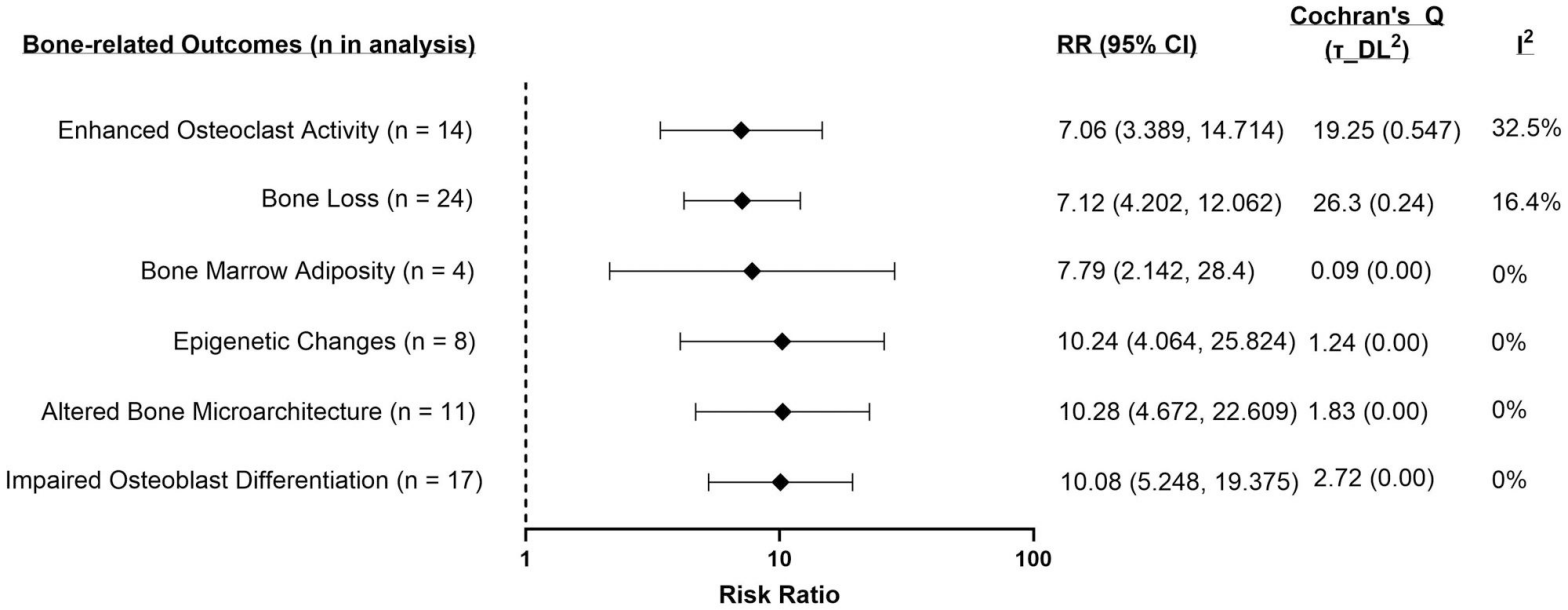
Forest plot of relative risks (RR) and 95% confidence intervals (CI) for bone-related outcomes associated with exposure. RRs were derived from reconstructed binary exposed-versus-control contrasts to harmonize heterogeneous biological endpoints across epidemiologic and experimental systems. These estimates are interpreted as indicators of directional consistency rather than adjusted causal risk estimates. Points represent RR estimates, and horizontal lines indicate 95% CIs. The vertical dashed line at RR = 1 denotes no association. Outcomes with CIs entirely above or below 1 are statistically significant at *P* < 0.05. Sample sizes (*n*) indicate the number of studies contributing to each estimate. Meta-analyses were performed in Python. Figure generated using GraphPad Prism.

Meta-analysis outcomes should be interpreted as a directional consistency hazard synthesis as opposed to a quantitative risk estimation since pooled calculated RRs encompasses several biological endpoint data. Because these reconstructed contrasts include experimental systems and binary exposed-versus-control comparisons across heterogeneous biological endpoints, the magnitude of pooled RRs reflects concordant adverse directionality under controlled conditions rather than population-level epidemiologic risk estimates. Across bone-related outcomes, exposure was consistently associated with increased hazard risk. The largest pooled effects were observed for impaired osteoblast differentiation and altered bone microarchitecture, followed by epigenetic changes (Fig. 2). Moderate effects were seen for bone marrow adiposity, bone loss, and enhanced osteoclast activity. Estimates were most precise for bone loss and osteoclast activity (*n* ≥ 14) and widest for bone marrow adiposity (*n* = 4), but the direction of effect was uniformly adverse across endpoints (Supplementary Materials—2×2 Tables). Study-level 2 × 2 tables and reconstructed log risk ratios contributing to each pooled estimate are provided in the Supplementary Materials to ensure transparency of the underlying data. For the outcome of bone loss, RR: 7.12; heterogeneity *Q* = 26.30, df = 22, *P* ≈ 0.24; *I*^2^=16.4%; τ^2^_DL = 0.241 (Duranova et al. 2014; Wu et al. 2014; Xu et al. 2014; Zhao et al. 2014, 2015; Liu et al. 2016, 2019, 2020; Tomaszewska et al. 2016; Buha et al. 2019; He et al. 2019; Zhang et al. 2020a; Luo et al. 2021; Ma et al. 2021a, 2021b; Chen et al. 2022; Torres-Rodríguez et al. 2022; Wang et al. 2022c; Song et al. 2023; Tong et al. 2023; Wan et al. 2023). Heterogeneity was low and not statistically significant, indicating study estimates were broadly consistent. For the outcome of impaired osteoblast differentiation, RR: 10.08; heterogeneity *Q* = 2.72, df = 16, *P* ≈ 1.00; *I*^2^=0%; τ^2^ ≈ 0, these study estimates were statistically consistent with a common effect (Wu et al. 2014, 2019; Xu et al. 2014; Zhao et al. 2014, 2015; Beier et al. 2015; Papa et al. 2015; Abnosi and Golami 2017; Liao et al. 2017; Al-Ghafari et al. 2019; He et al. 2019; Lv et al. 2019; Liu et al. 2020; Ma 2021a, 2021b; Wang et al. 2022c). For the outcome of bone marrow adiposity, RR: 7.79; heterogeneity *Q* ≈ 0.09, df = 3, *P* ≈ 1.00; *I*^2^=0%; τ^2^ ≈ 0, therefore these study estimates were statistically consistent with a common effect (Zhao et al. 2014; Beier et al. 2015; Abnosi and Golami 2017; Luo et al. 2021). For the outcome of epigenetic changes, RR: 10.24; heterogeneity *Q* ≈ 1.24, df = 7, *P* ≈ 1.00; *I*^2^ = 0%; τ^2^ ≈ 0 (Wu et al. 2014, 2019; Zhao et al. 2014; Li et al. 2021; Ma et al. 2021a; Chen et al. 2022; Wang et al. 2022b). The study estimates were statistically consistent with a common effect. For the outcome of enhanced osteoclast activity, RR: 7.06; heterogeneity *Q* = 19.25, df = 13, *P* ≈ 0.13; *I*^2^ = 30.3% (moderate); τ^2^_DL = 0.547 (Duranova et al. 2014; Wu et al. 2014; Brzóska et al. 2016; Liu et al. 2016, 2020; Tomaszewska et al. 2016; Luo et al. 2021; Ma et al. 2021a, 2021b; Chen et al. 2022; Torres-Rodríguez et al. 2022; Song et al. 2023; Tong et al. 2023; Wan et al. 2023). There was some between-study variability present. For the outcome of altered bone microarchitecture, RR: 10.28; heterogeneity *Q* ≈ 1.83, df = 10, *P* ≈ 1.00; *I*^2^ = 0%; τ^2^ ≈ 0; study estimates were statistically consistent with a common effect (Liu et al. 2016, 2020, 2024; He et al. 2019; Lv et al. 2019; Ma et al. 2021a, 2021b; Chen et al. 2022; Wang et al. 2022b, 2022c; Zhou et al. 2023) (Fig. 2). Study details from those included in the meta-analysis and GRADE summary are described in the sections Exposure to air pollution and/or PM and Exposure to heavy metals and exposure-related mechanisms.

### Exposure to air pollution and/or PM

**Fig. 3.**
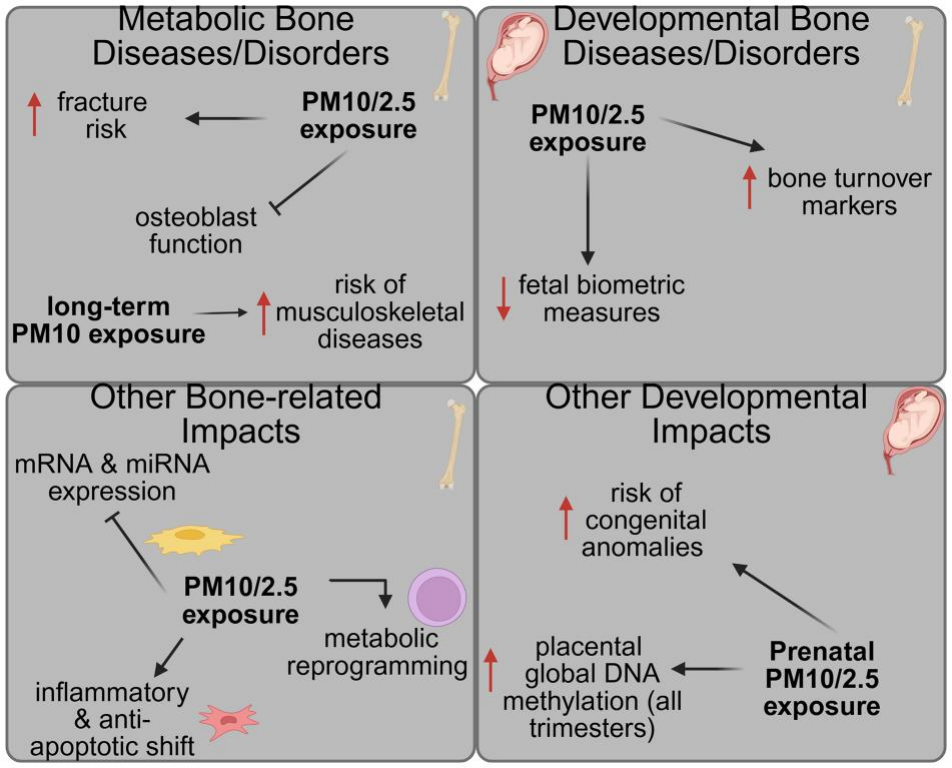
Effects of particulate matter (PM) and air pollution on bone and skeletal health. This is a hypothesized mechanism of action synthesizing relevant outcomes in this review. Chronic exposure to PM2.5 and PM10 is linked to increased risk of musculoskeletal disorders, fractures, and impaired fetal bone growth. Mechanistic evidence from experimental studies shows that PM disrupts osteoblast function, alters bone mineralization, and degrades skeletal architecture through oxidative stress, inflammation, and epigenetic modifications. Early-life exposures appear to cause alterations in bone development and homeostasis.

Figure 3 outlines hypothesized biological pathways linking PM exposure to a variety of mixed skeletal-related outcomes, summarizing the epidemiological and experimental evidence below.

#### Metabolic bone diseases/disorders

##### Epidemiological data of metabolic bone diseases/disorders

Cheng et al. (2024) conducted a prospective cohort study using the UK Biobank, which recruited adults aged 37 to 73 yr between 2006 and 2010 and followed participants until musculoskeletal disease diagnosis, death, loss to follow-up or December 31, 2019. The sample size included 271,321 participants, among whom 104,736 incident total musculoskeletal disease cases occurred over 1,135,752 person-years (median follow-up 10.85 yr) (Cheng et al. 2024). Cases were older (59.2 vs 55.39 yr), had higher BMI (28.03 vs 26.68), and were more often female with lower activity and socioeconomic status (Cheng et al. 2024). Exposure was via ambient outdoor air pollution (inhalation), estimated at each participant’s geocoded residential address using ESCAPE land-use regression model (annual average concentrations; PM2.5, PM2.5-10, NO2, and NOx) (Cheng et al. 2024). Mean baseline concentrations (µg/m^3^) among incident total musculoskeletal disease cases were PM2.5: 9.93, PM2.5-10: 6.39, NO2: 28.13, and NOx: 16.95. This study evaluates ambient PM rather than concentrated or experimentally generated PM exposures. In the fully adjusted Cox models (Model 2), higher PM2.5 was associated with increased risk of osteoporosis (HR = 1.064, 95% CI: 1.020–1.110) and dorsopathies (HR = 1.065, 95% CI: 1.039–1.091). PM2.5-10 showed similar patterns and were associated with inflammatory arthropathies (HR = 1.059, 95% CI: 1.037–1.081), degenerative joint diseases (HR = 1.035, 95% CI: 1.020–1.051), and total musculoskeletal diseases (HR = 1.021, 95% CI: 1.012–1.031). Per unit increase in the predicted annual-average concentration as modeled that each 10 µg/m³ increase in PM2.5 and 7 µg/m³ increase in PM2.5-10 was associated with higher risk of degenerative joint disease, dorsopathies, and osteoporosis (Table S1) (Cheng et al. 2024).

Qi et al. (2023) examined 446,395 UK Biobank participants who were free of fracture history at enrollment (2006–2010) and linked them to modeled residential (ambient outdoor) air pollution exposures (2007–2010), capturing inhalation-relevant exposure to annual-average ambient PM2.5, PM2.5-10, PM10, NO2, and NOx, as well as a weighted composite air pollution score. Exposure was assigned at the geocoded home address using land use regression approaches (ESCAPE-based models and EU-wide air pollution maps), emphasizing ambient (not concentrated) particle pollution at the residential location. Over a median 8-yr follow-up, 12,288 incident clinical fractures were documented. In fully adjusted Cox models, each 10 µg/m³ increase in PM2.5 was associated with a higher fracture risk (HR= 1.70, 95% CI: 1.42–2.03), whereas PM2.5-10 was not clearly associated. PM10 showed a smaller elevation (HR = 1.10, 95% CI: 1.00–1.21). Consistent with these continuous estimates, participants in the highest versus lowest quintile of the composite air pollution score had a 15.3% higher fracture risk (HR = 1.15, 95% CI: 1.09–1.22), with pollutant-specific top-to-bottom quintile contrasts of 16% for PM2.5 and ∼5% for PM10. Qi et al. (2023) further reported that a small portion of the air pollution-fracture association was mediated through serum 25(OH)D (e.g. 5.49% mediation for the composite score; similar modest mediation for PM2.5/10), supporting a plausible systemic pathway linking ambient mixtures to fracture risk (Table S1).

##### Experimental data of metabolic bone diseases/disorders

Ge et al. (2023) tested PM2.5 effects in mice using an inhalational whole-body exposure model. Specifically, twelve 5-wk-old male C57BL/6 mice were randomized (*n* = 6/group) to ambient PM2.5 versus filtered air (FA) for 6 mo (May–November 2018) in a controlled exposure system, where the PM chamber received ambient air with particles >2.5 µm removed by cyclone and the FA chamber removed PM2.5 using a HEPA filter (Ge et al. 2023). During the exposure period, PM2.5 was monitored in real time and chemically characterized, including trace metals (e.g. Al, Pb, Mn as major components) and other constituents. PM2.5-exposed mice exhibited a significantly shorter femur length compared with controls (14.8 ± 0.1 mm vs 15.0 ± 0.1 mm; *P* < 0.05), whereas femur width did not differ between groups. Consistent with impaired osteoblast maturation, the number of OSTEOCALCIN (OCN)-positive cells in distal femur sections was reduced (∼10 vs ∼15 cells per field; *P* < 0.05), and serum OCN concentrations were markedly lower in PM2.5-exposed mice (∼100 vs ∼150 ng/mL; *P* < 0.001). In contrast, bone-specific ALP levels increased (∼24 vs ∼8 U/L; *P* < 0.05), and ALP protein expression was upregulated (∼1.5 fold vs control; *P* < 0.05). Despite these alterations in bone turnover markers, micro-CT analysis showed no significant differences in cortical or trabecular bone mineral density (BMD) or trabecular microarchitecture parameters between groups. Collectively, these findings indicate that chronic whole-body inhalation exposure to ambient PM2.5 disrupts bone turnover and osteoblast maturation without overt changes in BMD under the exposure conditions tested (Supplementary Materials—Extraction) (Ge et al. 2023).

#### Developmental bone diseases/disorders

##### Epidemiological data developmental bone diseases/disorders

Evidence from pediatric and prenatal populations indicates that air pollution may alter bone development by accelerating turnover and impairing growth. In a German analysis of 10-yr-old children from the GINIplus and LISAplus birth cohorts (Munich and Wesel; *n* = 2,264), Liu et al. (2015) evaluated long-term (1 yr) modeled residential ambient air pollution exposures, including NO2, PM2.5, PM2.5-10, PM10, derived from ESCAPE land-use regression models, along with distance to a major road as a traffic-related exposure proxy. Children in LISAplus had higher OCN (99.1 ± 33.5 ng/mL) and C-terminal telopeptide of type I collagen (CTx) compared with GINIplus (OCN: 99.1 ± 22.5 vs 91.1 ± 30.5 ng/mL; CTx: 878.2 ± 346.5 vs 580.3 ± 290.9 ng/mL; *P* < 0.001), and Munich versus Wesel differences were also observed. In fully adjusted models (accounting for sex, age, BMI, pubertal status, fasting status, parental education, physical activity, and season). Both OCN and CTx were positively associated with coarse/fractional PM metrics: Per interquartile range increase, Pm2.5-10 was associated with ± 3.0 ng/mL OCN and ± 32.3 ng/L CTx, and PM10 with ± 3.2 ng/mL OCN and ± 30.7 ng/L CTx. Children living within ≤ 350 m of a major road also tended to have higher OCN and CTx (though not statistically significant), supporting a traffic-related exposure gradient. Notably, city-stratified analyses indicated Munich patterns were broadly similar to pooled estimates, whereas Wesel results were less consistent. Collectively, these findings suggest that ambient inhalation exposures (rather than concentrated PM dosing) may be linked to higher bone turnover during growth, consistent with the hypothesis that pollutant exposure could accelerate remodeling in developing skeletons (Supplementary Materials—Extraction) (Liu et al. 2015).

Complementary prenatal evidence supports impaired skeletal growth in utero following maternal inhalation exposure to ambient PM. In Shanghai, Cao et al. (2019) analyzed 7,965 pregnant women from the SMILE cohort (pregnancies between January 1, 2014 and April 20, 2015) and evaluated 29.926 repeated ultrasound examinations (1 to 10 per pregnancy) across gestation. Prenatal ambient PM2.5 exposure was assigned at the individual level using routine monitoring data (53 stations) and spatial estimation to residential address, with exposure summarized over pregnancy up to the week preceding each ultrasound; mean individual PM2.5 exposure was 51.2 µg/m^3^ (range: 29.1–82.1 µg/m^3^). In fully adjusted generalized estimating equation models controlling for demographic factors, co-pollutants, and meteorology, each 10 µg/m^3^ increase in PM2.5 was associated with smaller fetal biometry, including reduced abdominal circumference (AC: −5.48 mm), biparietal diameter (BPD: −5.57 mm), and femur length (FL: −5.47 mm), as well as lower estimated fetal weight (EFW: −14.49 h by Hadlock; −13.56 g by Shepard). Notably, the authors highlight that BPD and FL are more closely related to bone development, supporting the biological relevance of these ultrasound endpoints for skeletal growth. Together, these findings indicate that prenatal exposure to ambient PM2.5 is robustly associated with constrained fetal growth, including skeletal-relevant measures (FL/BPD), consistent with early-life susceptibility (Table S1) (Cao et al. 2019).

#### Other reported bone-related impacts

Beyond bone structure and turnover, PM disrupts gene expression, inflammatory signaling, and metabolism in progenitor cells.

##### Experimental data other reported bone-related impacts

Li et al. (2021) exposed 11-wk-old male C57BL/6J mice (*n* = 3/group) to concentrated ambient PM2.5 (CAP) via inhalation for 6 h/d over 30 consecutive days (mean CAP concentration: 67 ± 2.9 µg/m^3^) compared with filtered air controls. Following exposure, bone marrow-derived endothelial progenitor cells (EPCs) were isolated (then cultured for 10 d) and subjected to mRNA- and miRNA-sequencing. The authors identified 166 differentially expressed genes (122 upregulated; 44 downregulated) and 108 differentially expressed miRNAs (55 upregulated; 53 downregulated) in EPCs from CAP-exposed mice, implicating pathways relevant to EPC function and vascular biology. For example, *Ccl5* was among the upregulated genes (log2FC 2.5), and the study reported reciprocal mRNA–miRNA relationships (e.g. miR-214-3p/*Ccl5*, miR-450a-5p/*Dusp-10*, miR-92a-3p/*Tgfb2*) consistent with internal validation. Together, these findings suggest that inhaled PM2.5 (as CAP, i.e. a concentrated form of ambient PM2.5) can alter the mRNA and miRNA landscape of bone marrow EPCs, with potential implications for vascular remodeling and pathways that could intersect bone-vascular crosstalk (Supplementary Materials—Extraction) (Li et al. 2021).

Expanding on these cellular responses, Abu-Elmagd et al. (2017) examined human bone marrow-derived mesenchymal stem cells (BM-MSCs) obtained from five male osteoarthritis patients (52 to 64 yr) undergoing surgery, and exposed early-passage BM-MSCs (P3-4) in vitro to ambient PM2.5 or PM10 collected on filters (Jeddah, Saudi Arabia) and resuspended/sonicated into culture media (i.e. direct cellular dosing, not inhalation). BM-MSCs were treated with 15, 25, 50, 150, or 300 µg/mL for 24, 48, or 72 h, and proliferation/viability was assessed by MTT (absorbance at 570 nm). Viability showed a biphasic response for PM2.5, with increased proliferation at lower doses (mean percent increases at 48 h: 87.58% at 15 µg/mL; 86.93% at 25 µg/mL; 96.73% at 50 µg/mL; average ∼90% increase across these low-dose groups) and decreased proliferation at higher doses (mean percent decreases at 48 h: 11.11% at 150 µg/mL and 13.07% at 300 µg/mL; average ∼12.1% decrease), with reported changes statistically significant (*P* < 0.05), relative to their control MTT readout. In contrast, PM10 produced inhibition (e.g. mean percent decreases at 48 h: 23.71% at 50 µg/mL; 22.68% at 150 µg/mL; 31.96% at 300 µg/mL; average ∼26.1% decrease). Inflammatory gene expression responses were robust at 150 µg/mL for 48 h, with TNF-α increased 1.34-fold (PM2.5) and 5.80-fold (PM10), and IL-6 increased 3.54-fold (PM2.5) and 5.90-fold (PM10); the authors note that >2-fold upregulation met their statistical significance criterion. PM also shifted apoptotic/cell-cycle markers (mild *Bcl2* upregulation; *Bax* and p53 downregulation), though these particular fold-changes were reported as not statistically significant. Collectively, these findings indicated that ambient-derived PM, when delivered as a direct in vitro dose (µg/mL) to BM-MSCs, can induce dose-dependent proliferative effects and strong proinflammatory signaling in progenitor populations critical for skeletal repair (Supplementary Materials—Extraction) (Abu-Elmagd et al. 2017).

Bhattarai et al. (2023) exposed neonatal C57BL/6 mice (1.5 d old; *n* = 45/group) to inhaled PM2.5 using a whole-body atmospheric simulation chamber (ASC), delivering nebulized, atmospherically relevant artificial PM2.5 for 2 h/d over 5 consecutive days. Particles > 2.5 µm were removed using a filter, and the mean chamber PM2.5 concentration was 50.6 ± 11.6 µg/m^3^ (measured in the absence of mice). This design reflects a controlled inhalation exposure (i.e. not a personal ambient monitoring study), intended to model early-life exposure to fine PM under standardized conditions. Following exposure, the authors reported increased oxidative stress and inflammasome activation in the lungs and bone marrow, with downstream evidence of progressive hematopoietic stem cell (HSC) senescence emerging with aging (notably at 12 mo). In parallel, they observed preferential impairment of the bone marrow microenvironment, including age-related phenotypes consistent with increased osteoclastogenesis and adipogenesis, decreased osteogenesis, and reduced radioprotective potential in exposed mice (Supplementary Materials—Extraction) (Bhattarai et al. 2023).

#### Other reported developmental impacts

##### Epidemiological other reported developmental impacts

Farhi et al. (2014) analyzed a historical birth cohort of 216,730 infants in Israel (1997–2004), including 207,825 spontaneously conceived (SC) births and 8,905 assisted reproductive technology (ART) births, to evaluate whether ambient (outdoor) air pollution during pregnancy was associated with congenital malformations. Exposure was assessed was maternal residential ambient concentrations derived from air monitoring station data and assigned using a geographic information system with Kriging interpolation, separately from the first trimester and entire pregnancy (i.e. this was not a concentrated exposure paradigm). In fully adjusted models, higher PM10 and NOx during the entire pregnancy were each associated with a slightly increased odds of any congenital malformation (PM10: OR = 1.06, 95% CI: 1.01–1.11 per 10 unit increase; NOx: OR = 1.03, 95% 1.01–1.04 per 10-ppb increase). The authors reported that the signal was most evident for circulatory system malformations (PM10 and NOx) and genital organ malformations (NOx), whereas SO2 and O3 were not significantly associated with increased malformation risk in the overall cohort. Overall, these data support an association between prenatal PM10 exposure (inhalation-relevant ambient metrics) and risk of congenital anomalies, while also underscoring that the observed effects were modest and outcome-specific (Table S1) (Farhi et al. 2014).

Complementing these epidemiological associations, Maghbooli et al. (2018) conducted a nested case-control study in Tehran, Iran, enrolling 100 healthy pregnant women (< 14 wk gestation) from pre-selected high- versus low-pollution regions. After exclusions, 92 mother–newborn pairs were included in analyses. Maternal exposure reflected ambient outdoor PM (inhalation-relevant), estimated as regional background PM2.5 and PM10 (µg/m^3^) over pregnancy using monitoring station data mapped to 4×4 grids, with additional sport measurements using portable monitors. Average pregnancy exposures were higher in the high-pollution region (PM2.5: 37.12 ± 0.50 (high) vs 25.18 ± 0.69 µg/m^3^ (low); *P* = 0.0001 for both), with similar contrasts across trimesters. Placental global DNA methylation (reported as %5-mdC/(5-mdC + dC)) did not differ between regions overall (2.59 [0.70] vs 2.44 [0.86], *P* = 0.42), but first-trimester PM2.5 and PM10 were positively correlated with placental global DNA methylation (PM2.5: *P* = 0.03; PM10: *P* = 0.01). In contrast, birth outcomes (gestational age, weight, length, head circumference, Apgar scores) showed no significant correlations with PM2.5 and PM10 across pregnancy or by trimester (Supplementary Materials—Extraction) (Maghbooli et al. 2018).

Taken together, these results suggest that maternal PM exposure can influence development both through structural anomalies and via epigenetic reprogramming, potentially shaping long-term health trajectories even in the absence of immediate perinatal changes.

### Exposure to heavy metals and exposure-related mechanisms

Heavy metals commonly found as PM constituents (Pb, Cd, Hg, As), as well as internal metal biomarkers, were associated with adverse skeletal outcomes in the studies included. PM-bound metals can be inhaled and subsequently translocated systemically, whereas biomarker-based measures may reflect multi-route exposure and cumulative body burden. Once systemically available, these metals preferentially accumulate in bone, where they interfere with remodeling and development through oxidative stress, signaling disruption, and epigenetic regulation.

#### Reduced BMD, risk of osteoporosis, mineral loss

##### Epidemiological data

Wang et al. (2022b) evaluated adults with environmental Pb and Cd exposure recruited from a polluted community and a lower-exposure control area in China (final analytic sample: 194 women and 108 men, excluding occupationally exposed individuals). Pb and Cd exposure were quantified in blood and urine using graphite-furnace atomic absorption spectrometry, and the authors note that these metals can enter the body via the digestive tract, with bone as a key toxicity target. BMD was measured at the proximal forearm (nondominant arm) by peripheral DXA (pDEXA), osteoporosis was defined using a T-score ≤ −2.5, and anemia was defined as Hb < 130 g/L (men) or < 120 g/L (women). In fully adjusted models (accounting for age, BMI, Pb/Cd metrics, smoking/drinking, and kidney-related covariates), men with anemia had substantially higher odds of osteoporosis versus men with normal Hb (OR = 11.28, 95% CI: 1.94–65.54; and OR = 19.56, 95% CI: 2.98–128.78), whereas no analogous association was observed in women. This supports the framing that higher blood Pb correlates with lower BMD, particularly among anemic males, consistent with both direct skeletal (Pb/Cd) and systemic/hematologic vulnerability (anemia modifying skeletal risk) (Wang et al. 2022c).

Low-level heavy metal exposure is associated with reduced bone density in population data. Using nationally represented KNHANES data (2008–2011), Lim et al. (2016) analyzed 2,429 Korean adults (≥ 18 yr) and classified bone status by DXA-derived T-scores from the total proximal femur, femoral neck, and lumbar spine (L1–L4) (normal, osteopenia, osteoporosis). Blood metals were assessed from venipuncture samples, with blood Pb and Cd quantified by graphite furnace atomic absorption spectrometry (and Hg by gold amalgamation). In fully adjusted logistic models, individuals in the highest blood Pb quartile (≥ 2.933 µg/dL) had higher odds of osteopenia/osteoporosis compared with the lowest quartile (OR = 1.49; 95% CI: 1.12–1.97). Similarly, the highest blood Cd quartile (≥ 1.439 µg/L) was associated with increased odds (OR = 1.8; 95% CI: 1.35–2.4). Notably, Lim et al. (2016) report that Pb, Hg, and Cd concentrations were within WHO “normal range,” yet bone-loss risk still increased, supporting concern for skeletal impacts even at relatively low environmental exposures. Together, these studies support reduced BMD/osteoporosis as a reproducible human endpoint across both community-based and nationally representative cohorts, including at relatively low environmental exposure levels.

##### Experimental data

Brzóska et al. (2016) evaluated chronic Cd exposure in a rat model using oral dietary administration at 1 mg Cd/kg diet (environmentally relevant/low) and 5 mg Cd/kg diet for 3–4 mo, with bone collected at necropsy (including femurs/tibias). In trabecular-rich distal femoral epiphysis samples, the authors quantified oxidative/antioxidative status using enzymatic and nonenzymatic antioxidant markers (e.g. GPx, GR, SOD, CAT, GSH, total thiols), global oxidative indices (TAS, TOS, OSI), and oxidative damage endpoints (e.g. protein carbonyls, 8-iso-prostaglandin, lipid peroxidation, 8-OHdG). Across exposure durations, Cd increased bone oxidative stress even at the lower dose, including elevated H2O2 and higher TOS/OSI, alongside increases in oxidative damage markers such as protein carbonyls and 8-isoP. Cd also reduced components of the bone antioxidative barrier (e.g. decreases in total thiols at multiple timepoints), supporting a persistent pro-oxidant shift in the bone microenvironment. Consistent with this, the authors note that Cd, even at low concentrations, may directly influence bone tissue cell activity and the oxidative/antioxidative balance, providing mechanistic plausibility for skeletal impairment under low-level, chronic exposure conditions (Brzóska et al. 2016).

#### Altered bone-metal burden and mineral composition

##### Epidemiological data

Buha et al. (2019) paired an experimental endpoint (see below) to a real-world context by analyzing human femoral head samples (*n* = 20), quantifying bone Cd content, trabecular BMD (tBMD), and mineral measures. They found that higher bone Cd content correlated with lower tBMD and lower bone Ca content, supporting the interpretation that long-term (“natural”) Cd accumulation in bone is associated with poor mineral status in humans (Buha et al. 2019). This human data is consistent with the possibility that cumulative skeletal metal burden may lead to impaired mineral status and remodeling, mechanisms directly testable in controlled models.

##### Experimental data

Buha et al. (2019) assessed Cd-related bone effects in an in vivo rat model. Male Wistar rats (6–8 wk; 150–200 g) received oral Cd by gavage for 28 d across a dose range (0.3–10 mg Cd/kd body weight/day). Using a femur mineral composition as a sensitive skeletal endpoint, they reported significant reductions in femur calcium and phosphorus even at the lowest dose. Femur Ca decreased by ∼9–15% (mean ∼12%) and phosphorus decreased by ∼5–15% (mean ∼9%), with effects evident at 0.3 mg/kg/d and remaining similarly depressed across higher doses (Buha et al. 2019). These findings indicate that bone mineral endpoints respond at relatively low experimental Cd exposures, consistent with early skeletal sensitivity.

Using a controlled dietary exposure model in animals, Liao et al. (2017) evaluated subchronic bone effects of molybdenum (Mo) and/or Cd in 11-d-old male ducklings (*n* = 120) assigned to six diet groups for 120 d (control; 15 mg/kg Mo; 100 mg/kg Mo; 4 mg/kg Cd; 4 mg/kg Cd + 15 mg/kg Mo; or 4 mg/kg Cd + 100 mg/kg Mo). At days 60 and 120, they collected serum, excreta, and metatarsals and quantified trace elements and minerals (Mo, Cd, Ca, P, Cu, Fe, Zn, Se) using atomic absorption spectroscopy/UV methods, measured serum ALP activity, and assessed bone structure by radiography and histopathology. Overall, co-exposure groups showed increased Mo and Cd accumulation in metatarsal bone alongside decreased Ca and P, with higher Ca and P in excretion and increased ALP activity, consistent with disrupted mineral homeostasis and altered bone mineral deposition. Radiographic and histologic evaluations further indicated osteopenia/osteoporotic lesions with fewer and thinner trabecular structures in the combined exposure groups, supporting diet-derived metal exposure as sufficient to induce measurable skeletal damage (Liao et al. 2017).

#### Suppressed osteoblastogenesis and impaired mineralization

##### Experimental data

Beier et al. (2015) evaluated how low-level Pb exposure alters osteogenic signaling and skeletal accrual in juvenile male C57BL/6J mice. Dams received 0 or 50 ppm Pb acetate in drinking water beginning at delivery, and male offspring remained on the same water exposure after weaning. At 5 wk of age, mice were placed on either a low-fat diet (10% kcal fat) or high-fat (HFD; 60% kcal fat) and were harvested after 3, 6, or 12 wk for bone and metabolic phenotyping. In vivo, Pb (alone and with HFD) produced trabecular bone deficits and altered progenitor fate, promoting osteoclastogenesis and adipogenesis while suppressing osteogenesis, with accompanying evidence of reduced WNT/CTNNB1 activity. Mechanistically, the authors used MC3T3-E1 osteoblastic cells to probe pathway-level effects. After 24 h exposure, they assessed mRNA expression, and in luciferase reporter assays cells were treated for 48 h with Wnt3a (100 ng/mL) alone or with Pb (1 or 5 µM), and/or nonesterified fatty acids (NEFA; 400 µM), using reporters including TOPFLASH (Wnt/β-catenin) and a 7-kb human SOST promoter reporter (SOST-Luc). Notably, they report that both serum sclerostin and SOST gene expression doubled with Pb exposure, and that Pb/NEFA inhibit β-catenin activity, consistent with Wnt pathway restriction as a central mechanistic node (Beier et al. 2015).

To strengthen causal interference for sclerostin-mediated restriction of Wnt signaling as a mechanism that can suppress osteoblast function, Beier et al. (2017) used Sost knockout (SOST-KO) mice in bone marrow osteogenesis assays. Bone marrow stromal cells were cultured in osteogenic α-MEM (with β-glycerol phosphate and ascorbate) and assessed for alkaline phosphatase activity at 10 d and matrix mineralization by alizarin red staining at 21 d. Adipogenesis was assessed via Oil Red O staining. Under dexamethasone (Dex) exposure, wild-type cultures showed impaired mineralization, whereas SOST-KO osteoblasts formed more robust bone nodules (∼2 fold vs WT) and were significantly resistant to Dex’s negative effect. Dex also increased adipogenesis (1.0 µmol/L Dex; 5.9-fold Oil Red O increase in WT), an effect not observed in SOST-KO cultures (Beier et al. 2017). Together, these data support that the upregulation of SOST can functionally “brake” osteoblast mineralization via Wnt pathway restriction, making SOST a biologically plausible mediator of pollutant-associated suppression of osteogenesis when Pb increases circulating SOST.

Suppressed WNT/CTNNB1 (canonical Wnt/β-catenin) signaling is one route by which metal toxicants can impair osteoblast function and mineralization. Papa et al. (2015) treated human osteoblast-like Saos-2 cells with CdCl2 (10 µM) in 1% FBS and assessed early versus longer exposure windows (6, 15, and 24 h). Under these conditions, Cd initially triggered β-catenin nuclear translocation and increased expression of Wnt/β-catenin target genes at 6 h, but with prolonged exposure (15–24 h), Wnt transcriptional machinery was suppressed, reflected by reduced TCF-1/LEF-1 levels and declining c-Myc expression relative to the early response. This shift coincided with broader osteoblast homeostasis disruption, including reduced OCN at 15–24 h and a more pro-osteoclastogenic profile (increased RANK-L with decreased OPG, increasing RANK-L/OPG ratio) (Papa et al. 2015). Collectively, these results support that sustained Cd exposure can inhibit Wnt/β-catenin signaling in osteoblast-like cells, a plausible mechanism contributing to impaired osteogenic function.

Wu et al. (2019) investigated whether Cd impairs osteogenic differentiation by disrupting Wnt/β-catenin signaling using primary BM-MSCs harvested from 3-wk-old Sprague–Dawley rats and maintained as a submerged monolayer culture in α-MEM-based growth medium at 37°C/5% CO2 (passaged 3–5). For osteogenic experiments, BM-MSCs were seeded in 24-well plates (7×10^4^ cells/well), exposed to CdCl2 (0, 0.1, or 0.2 µM) (added directly to culture medium) with or without Wnt3a, and then induced with osteogenic induction medium. Endpoints include ALP staining at day 10 and Alizarin Red S staining at day 14 to assess mineralization. In parallel, ALP was quantified using a pNPP assay read at 405 nm after exposure to 0–0.2 µM CdCl2 ± Wnt3a during osteogenic induction. Mechanistically, CdCl2 exposure reduced protein levels in the Wnt/β-catenin pathway (including Wnt3a, β-catenin, LEF1, TCF2), and Wnt3a co-treatment (50 ng/mL) rescued pathway protein levels and osteogenic outcomes (including ALP/Runx2 and mineralization readouts) (Wu et al. 2019).

Wan et al. (2023) modeled metal-induced suppression of bone formation by treating human BM-MSCs submerged in osteogenic induction medium containing CdCl2 (0, 2.5, or 5.0 µM) for 14 d (media refreshed every 2 d). Mineralization was quantified by Alizarin Red S staining (40 µM, 20 min), following fixation with 10% formaldehyde (10 min), revealing that 2.5–5.0 µM CdCl2, strongly reduced calcification nodule quantity and volume. Mechanistically, CdCl2 suppressed BMP/SMAD signaling, BMP-4 protein decreased by 76.77%, SMAD by 52%, and the p-SMAD1/5/9 complex by 31%, supporting a pathway-level explanation for impaired mineral deposition. Consistent with BMP-4 being functionally limiting, adding recombinant BMP-4 (50 ng/mL) during CdCl2 exposure improved mineralization and increased osteogenic regulators (Runx2, OSX) and downstream SMAD signaling compared with CdCl2 alone (Wan et al. 2023).

In osteoblast-lineage cells, As downregulates key regulators of differentiation and mineralization. In a rat bone marrow stromal cell (BMSCs) osteogenic differentiation mode (submerged culture), Wu et al. (2014) treated BMSCs with 0.5–1 µM As2O3 over 5–20 d and observed significant decreases in ALP activity (days 5 to 7), calcium deposition/absorption (day 14), and osteoblast mineralization (days 14 and 20), alongside reduced mRNA expression of Bmp2 (day 5) and osteocalcin/OCN (days 10 to 14). Mechanistically, low-dose As2O3 increased ERK phosphorylation during differentiation, and the ERK inhibitor PD98059 (10 to 20 µM) reversed As2O3-associated suppression of RUNX2 protein, ALP activity, mineralization, and Bmp2/OCN expression, supporting an ERK–RUNX2 axis in As-impaired osteoblastogenesis. In vivo, drinking-water exposure to 0.05 or 0.5 ppm As2O3 for 12 wk decreased trabecular and cortical bone parameters, including BMD, BV/TV, and bone thickness measures, indicating that these differentiation defects translate to measurable structural deficits under environmentally relevant exposure routes (Wu et al. 2014).

Xu et al. (2014) reported a biphasic, dose-dependent effect of arsenic trioxide (ATO) on osteoblast growth using submerged in vitro culture of human hFOB1.19 osteoblasts (F12 + 10% FBS). Low dose ATO (0.25 to 1 µM, 24 to 72 h) increased viability and promoted collagen synthesis, mechanistically linked to upregulated TGF-β1 and activation of p-AKT signaling. At the higher doses (5 to 20 µM), ATO reduced viability and induced apoptosis, with ultrastructural evidence of mitochondrial injury (e.g. swelling and cristae disruption) and nuclear changes, accompanied by increased Bax/Bcl-2 ratio and CASPASE-3 activation, consistent with mitochondria-linked apoptotic signaling at cytotoxic concentrations (Xu et al. 2014).

Lv et al. (2019) modeled chronic low-dose Cd effects on osteoblast precursor programming using an in vivo oral exposure paradigm followed by ex vivo MSC assays. Female weaning Sprague–Dawley rats were intragastrically administered CdCl2 at 1 or 2 mg/kg body weight/day (days/wk) for 38 wk. After exposure, BM-MSCs were isolated from femurs, expanded to low passage, and induced toward osteogenesis in vitro. Osteogenic gene expression was measured by RT-qPCR during the early differentiation stage (day 7). Chronic Cd exposure altered bone remodeling signals (RANKL/OPG) and suppressed key osteogenic differentiation genes. Osterix/Sp7 was decreased in both dose groups (e.g. 3.70-fold decrease at 1 mg/kg), and in the 2 mg/kg group Sp7/Osterix, Osteopontin (Spp1), COL1a2, and RUNX2 were decreased by 1.79-, 1.67-, 1.45-, and 1.35-fold, respectively, relative to controls. Notably, ALPL expression was not significantly different, indicating Cd’s earliest impacts seen in this model were on transcriptional regulators and matrix-associated osteogenic genes rather than ALP at this time point (Lv et al. 2019).

In submerged in vitro cultures of chicken embryos BM-MSCs, Cd (5 µmol/L) inhibited proliferation and osteogenic differentiation (reduced ALP staining and mineralized nodule formation) while increasing apoptosis and disrupting mitochondrial membrane potential. Co-treatment with 1α,25-(OH)2D3 (10 nmol/L; 6 h pretreatment) partially attenuated these effects (Tong et al. 2023).

#### Enhanced osteoclastogenesis and proresorptive signaling

##### Experimental data

In osteoclast precursors, Cd promotes differentiation by inhibiting mTORY-P70S6K1 signaling. In an in vitro (submerged, plate-based) osteoclastogenesis model, Wang et al. (2022a) differentiated murine RAW264.7 osteoclast precursors using RANKL (50 ng/mL) for 4 d while exposing cultures to CdCl2 (0.025 to 0.075 µM). Low-dose CdCl2 (particularly 0.025 to 0.050 µM) did not reduce viability (and increased proliferation over 4 d at 0.025 to 0.050 µM), whereas ≥ 0.1 µM decreased viability, so subsequent mechanistic work focused on the low-dose range. Under these differentiation conditions, CdCl2 increased osteoclastogenesis, evidenced by higher expression of *TRAP/Acp5*, *Ctsk*, and *Rank*, increased TRAP activity, and an increased proportion of TRAP+ multinucleated osteoclasts at 0.025 to 0.050 µM. Mechanistically, CdCl2 exposure suppressed mTOR pathway activity, decreasing p-mTOR/mTOR and p-P70S6K1/P70S6K1 during RANKL-induced differentiation. Functional perturbations supported causality; chloroquine pretreatment for 3 h attenuated Cd-enhanced osteoclast differentiation, and MHY1485 pretreatment for 3 h (an mTOR activator) increased mTOR/P70S6K1 signaling and reduced Cd-associated autophagy/osteoclastogenic effects (Wang et al. 2022a). Collectively, these data support that low-dose Cd can enhance osteoclast differentiation in precursors via mTOR-P70S6K1 inhibition and downstream autophagy signaling.

Cd exposure also altered osteoblast-derived regulators of osteoclastogenesis. In an in vitro submerged culture system, Wang et al. (2022b) treated primary osteoblasts with CdCl2 (0, 30, 60 nM/L) for 3 d and quantified coupling factors by qPCR and western blot. Cd reduced osteoblast activity (ALP/mineralization) and produced a proresorptive signaling shift, with RANKL upregulated and OPG downregulated at both transcript and protein levels, supporting a mechanism by which low-level Cd can enhance differentiation through altered osteoblast–osteoclast crosstalk (Wang et al. 2022b).

Using murine bone marrow-derived monocytes (from 5- to 6-wk-old C57BL/6 mice) as osteoclast precursors, Liu et al. (2020) exposed differentiating cells to Cd (CdCls; 20 to 100 nM) in the presence of RANKL and M-CSF and observed increased osteoclastogenesis, including greater TRAP+ multinucleated osteoclast formation and upregulation of osteoclast differentiation markers (e.g. TRAP, CK, CAII) alongside increased NFATc1 protein. Mechanistically, Cd elevated intracellular Ca2+, and blocking intracellular Ca2_ signaling attenuated these effects. 2.5 µM BAPTA-AM reduced Cd-associated Ca2+ oscillations and osteoclast marker induction, whereas 1 µM 2-APB (inhibiting ER Ca2+ release) reversed Cd-induced increases in osteoclast differentiation-associated proteins. Downstream, inhibition of the Ca2+/calmodulin/CaMK axis (KN-93 1 µM, STO-609 1 µM, W-7 5 µM, 30 min pretreatment) suppressed Cd-driven osteoclast differentiation and reduced Cd-associated increases in TRAP and NFATc1, supporting a Ca2/CaM/CaMK-NFATc1 signaling mechanism for Cd-enhanced osteoclastogenesis (Liu et al. 2020).

Arsenic disrupts bone remodeling by enhancing resorption through redox-sensitive osteoclastogenic signaling. Liu et al. (2019) tested this mechanism using 40-wk-old female Nrf2+/+ and Nrf2−/− mice exposed to 5 ppm sodium arsenite in drinking water for 16 wk. A regimen chosen to approximate internal As levels relevant to endemic human exposures. Mico-CT and DXA analyses showed that bone loss was markedly worse in Nrf2−/− mice than in wild-type controls following iAs exposure, consistent with heightened susceptibility when antioxidant defenses are impaired. Complementary in vitro experiments using RAW264.7 cells and BMMs demonstrated that low-dose iAs (0.25 to 0.5 µM) increased RANKL/M-CSF-driven osteoclast differentiation (TRAP+ multinucleated cells) and upregulated osteoclast resorption genes, with stronger effects under Nrf2 deficiency/knockdown. Mechanistically, Nrf2 loss amplified iAs-induced ROS accumulation (including H_2_O_2_) and p38 phosphorylation, with downstream elevation of NFATc1. Importantly, ROS scavenging with N-acetylcysteine (functional ROS scavenger) or p38 inhibition (SB203580) abolished p38 activation and reversed the iAs-associated increase in osteoclastogenesis, supporting a ROS-p38-NFATc1 pathway driving As-enhanced bone resorption (Liu et al. 2019).

In murine models, Cd engages purinergic and cytoskeletal signaling linked to osteoclast function. Ma et al. (2021a) exposed BALB/c mice to Cd via drinking water (25 mg/L) for 4 mo (short-term) or 15 mo (long-term) and paired these in vivo exposures with submerged in vitro differentiation of bone marrow macrophages (BMMs) and bone marrow stromal cells (BMSCs) treated with CdCl2 (0, 0.1, 1 µM) under MCSF/RANKL conditions. Mechanistically, Cd altered the P2X7–PI3K–AKT axis. During differentiation, Cd reduced P2X7 expression and was associated with suppressed PI3K/AKT phosphorylation, consistent with impaired osteoblast/osteoclast differentiation. In contrast, under a short-term late-stage osteoclast exposure (BMMs differentiated with M-CSF/RANKL for 4 d, then treated with 1 µM Cd for 12 h), Cd increased osteoclast adhesion/cytoskeletal remodeling alongside increased PI3K/AKT phosphorylation despite decreased P2X7 (Ma et al. 2021a).

Ma et al. (2021b) investigated Cd-induced skeletal toxicity using duck BM-MSCs and BMMs exposed to Cd during osteoblast and osteoclast differentiation for 5 d, alongside in vivo Cd exposure in duck embryos. In vitro, Cd inhibited osteoclast formation and suppressed resorption, and reduced expression of osteoclast differentiation proteins (including TRAP, c-Fos, and NFATc1), while increasing the OPG/RANKL ratio, consistent with an anti-osteoclastogenic shift under their assay conditions. Mechanistically, Cd exposure decreased intracellular ATP-associated signaling, downregulated P2X7, and reduced PI3K/AKT phosphorylation in differentiating BM-MSCs and BMMs, indicating inhibition (not activation) of the P2X7-PI3K-AKT pathway (Ma et al. 2021a).

Liu et al. (2024) evaluated whether low-level Cd exposure promotes a proresorptive bone phenotype using a mouse model with myeloid-specific Nrf2 deletion. Male mice (14 wk old) were exposed to CdCl2 (100 mg Cd/L) via drinking water for 8 or 16 wk, after which femoral bone outcomes were assessed by micro-CT, TRAP histology, and plasma bone turnover markers. Cd exposure was associated with trabecular bone deterioration and increased osteoclast number/surface, and Cd-exposed Nrf2(M)-KO mice had higher plasma TRAP5b and CTX-I compared with corresponding controls, consistent with increased bone resorption. Notably, plasma RANKL and OPG (as well as P1NP and osteocalcin) did not differ by genotype or Cd exposure status, indicating that the resorptive phenotype occurred without measurable systemic shifts in circulating RANKL/OPG and may instead reflect local or downstream osteoclastogenic regulation (Liu et al. 2024).

#### Oxidative stress and mitochondrial dysfunction as a cross-cutting driver

##### Experimental data

Oxidative stress contributes to impaired osteogenesis and bone loss by impairing osteoblast bioenergetics and promoting mitochondrial dysfunction. In an in vitro study of human osteoblasts, Al-Ghafari et al. (2019) exposed cells to Pb (55 µM) or Cd (30 µM) for 24 h (MTT IC50 conditions) and found significant evidence of mitochondrial injury, including reduced mitochondrial membrane potential, decreased mitochondrial complex I and III activity, and lower oxygen consumption rate (OCR), accompanied by increased lactate production, consistent with a shift away from aerobic metabolism. In parallel, metals increased intracellular ROS (DCFDA assay; +39% with Pb and +54% with Cd) and suppressed antioxidant defenses (reduced catalase and SOD-1 activity and reduced glutathione), while increased lipid peroxidation products (TBARS), supporting a redox-stress mechanism that could plausibly contribute to impaired osteogenesis and/or dysregulated remodeling (Al-Ghafari et al. 2019).

He et al. (2019) evaluated a potential mitigation strategy for Cd-associated osteoblast injury using an in vitro, submerged culture model of MC-3T3-E1 osteoblasts. Cells were pretreated with geniposide (100, 200, or 400 µg/mL) for 24 h and then challenged with CdCl2 (20 µM) for 3 h. Under these conditions, geniposide dose-dependently improved cell viability and reduced CdCl2-induced apoptosis and ROS levels as assessed by flow cytometry. Consistent with an antioxidant mechanism, geniposide also modulated oxidative stress-related factors (MDA, LDH, SOD) measured by ELISA and increased expression of the Nrf2 pathway and downstream targets (HO-1, NQO1) at the mRNA and protein levels (He et al. 2019).

Hu et al. (2023) investigated Cd-induced skeletal toxicity using both in vitro and in vivo models to link cellular mechanisms with bone outcomes. In primary BM-MSCs (submerged culture), cells were treated with CdCl2 (0 to 10 µM) for 12 h and assessed for autophagy/lysosome function and inflammatory signaling. Cd exposure increased autophagosome abundance (e., MDC signal, LC3 puncta/LC3-II) and shifted flux markers toward autophagic flux obstruction (p62 up; Atg5/Atg7 down), consistent with impaired degradation rather than simply increased autophagosome formation. Lysosomal function was also suppressed (reduced LysoTracker signal/acidification) and fusion machinery was disrupted (Rab7 down), supporting blockage at the autophagosome–lysosome stage. Mechanistically, Cd activated an oxidative stress–inflammation axis, upregulating ROS/NLRP3/Caspase-1/IL-1β signaling, with pharmacologic modulation (e.g. rapamycin; NLRP3 inhibitor) partially alleviating Cd-associated apoptosis/inflammatory phenotypes. In Sprague Dawley rats, the authors modeled chronic exposure via CdCl2 in drinking water for 4 mo and then evaluated Cd burden (ICP-MS) and mandibular bone structure (micro-CT), reporting mandibular bone loss with reductions in BMD and BV/TV, linking systemic exposure to craniofacial osteoporosis-like outcomes (Hu et al. 2023).

These cellular effects can translate into structural and neuro-sensory deficit in vivo. In an adult male C57BL/6J mouse model, chronic Cd exposure via drinking water (CdCl2, 25 mg/L for 16 wk) induced pain-related behaviors (mechanical hypersensitivity and reduced rearing activity) and produced trabecular bone deterioration at the femoral neck and L5 vertebra by micro-CT, alongside reduced CGRP+ and PGP9.5 sensory nerve fiber density in the femoral neck, supporting neuro-skeletal toxicity (Torres-Rodríguez et al. 2022).

#### Cell fate disruption: apoptosis, autophagy, senescence, and loss of regenerative capacity

##### Experimental data

Zhao et al. (2015) investigated Cd-induced apoptosis in primary rat osteoblasts derived from calvariae of 18- to 19-d gestational Sprague–Dawley rats cultured under standard submerged conditions (DMEM + 10% FBS, 5% Co2). Cells were exposed to Cd acetate (0 to 20 µM; 0 to 48 h), with 1 to 5 µM used for mechanistic assays after viability screening (IC50 ∼ 2 µM at 24 h, ∼45% viable). Cd increased apoptosis by Annexin V/PI and nuclear morphology, including an ∼doubling of apoptotic cells at 2 µM, 24 h, alongside mitochondrial injury (e.g. mitochondrial membrane potential decreased by ∼35% at 5 µM, 24 h) and a proapoptotic shift in BCL2 family signaling (Bax up, Bcl-2 down; Bax/Bcl-2 ratio ∼2.96-fold higher at 24 h). Consistent with mitochondrial apoptosis, Cd increased caspase-3 activity and cleavage of PARP, caspase-9, and caspase-3, and apoptosis was attenuated by a pan-caspase inhibitor (Z-VAD-fmk; 50 µM). Cd also activated MAPK signaling (phosphorylation of p38, ERK1/2, and JNK) and MAPK inhibitors (SB203580, U0126, SP600125; 10 µM preincubation) and reduced the apoptosis-associated Bax/Bcl-2 response, supporting involvement of caspase-dependent and MAPK-mediated mitochondrial pathways (Zhao et al. 2015).

Using primary BM-MSCs, Luo et al. (2021) showed that Cd directly induces a senescent phenotype consistent with skeletal regenerative capacity. In vitro, BM-MSCs exposed to Cd (10 µM, 24 h) exhibited increased senescence-associated β-galactosidase (SA-β-gal) staining, reduced proliferative capacity by EdU labeling, and upregulation of canonical senescence markers p21/p53/p16INK4a. Mechanistically, Cd activated NK-ĸB signaling, increasing RelA/IĸBα phosphorylation and promoting RelA nuclear translocation. Pharmacologic inhibition with the p65 inhibitor SC75741 (2 µM pretreatment) partially attenuated Cd-induced senescence readouts. Cd also increased total ROS and mitochondrial ROS and induced DNA damage, alongside mitochondrial impairment (mtDNA copy number reduced by ∼50% after 24 h), supporting a mitochondria-ROS-damage axis that can reinforce senescence and SASP-associated inflammatory signaling. Functionally, Cd exposure was also evaluated in the context of osteogenic differentiation (e.g. 0.1 µM Cd during 7 d of differentiation/ALP staining), aligning senescence with impaired osteogenic programming (Luo et al. 2021). In vivo, the authors further report that chronic Cd exposure increased marrow adiposity, reduced mineralization, and delayed bone repair in a skull defect model, linking BM-MSC aging phenotypes to structural/repair deficits (Luo et al. 2021).

Zhou et al. (2023) investigated Cd-induced osteoblast senescence and DNA damage signaling using both in vitro submerged culture and in vivo chronic exposure models. In vitro, osteoblasts exposed to CdCl (20 to 80 µM) exhibited increased senescence markers (p53, P16^INKa, P21^CIP1) and higher SA-beta-gal positivity, consistent with a senescent phenotype. Mechanistically, Cd exposure triggered a DNA damage response: Comet assays showed increased tail DNA/tail length with rising CdCl2, and immunoblotting demonstrated decreased SIRT1 alongside ATM phosphorylation (p-ATM) and H2AX phosphorylation (gammaH2AX). gammaH2Ax induction was further supported by immunofluorescence (Zhou et al. 2023). In vivo, the authors modeled long-term exposure via CdCl2 in drinking water (50 mg/L) for 18 mo, which induced an osteoporotic phenotype with decreased BMD and trabecular microstructural disruption. Osteoblastic SIRT1 overexpression attenuated senescence readouts in bone tissue and reduced DNA-damage response marker staining (p-ATM, γH2AX) (Zhou et al. 2023).

In experimental models, Cd disrupts osteocyte homeostasis, in part, by suppressing PI3K-AKT-mTOR signaling and inducing autophagy. In the long-term mouse exposure paradigm, Song et al. (2023) administered Cd via drinking water (5 mg/L and 25 mg/L) for 16 mo (*n* = 11 mice/group) and observed reduced PI3K/AKT/mTOR pathway activation in bone, reflected by decreased p-PI3K/PI3K, p-AKT/AKT, and p-mTOR/mTOR ratios. Complementary submerged in vitro experiments used MLO-Y4 osteocytes cultured in complete DMEM and treated with Cd (0, 20, 40, 80 µM) for 6 h, where 80 µM Cd produced clear injury-associated morphological changes and increased autophagy signaling (increased LC3II, decreased p62). Mechanistically, 80 µM Cd increased autophagy proteins (ATG5/7, LC3II) while inhibiting phosphorylation of PI3K, AKT, and mTOR. Importantly, rapamycin (RAPA; 5 µM, 6 h) co-treatment further enhanced autophagy (increased LC3II, decreased p62) and alleviated Cd-induced osteocyte damage, supporting autophagy as a compensatory/protective response in Cd-exposed osteocytes rather than the primary driver of injury (Song et al. 2023).

Ran et al. (2023) provided mechanistic evidence that Cd can engage DNA damage-responsive stress signaling and promote osteoblast dysfunction through suppression of SIRT1, a deacetylase that restrains p53-driven apoptosis and senescence programs under oxidative stress and DNA damage conditions. In vivo, long-term CdCl2 exposure via drinking water (50 mg/L for 18 mo) induced an osteoporotic phenotype in rats consistent with impaired osteogenesis (e.g. decreased ALP), and the authors concluded this reflected inhibition of bone formation rather than increased osteoclastogenesis (Ran et al. 2023). In vitro, osteoblast-like ROS1728 cells treated with CdCl (0 to 80 µM for 6 h) showed dose-dependent apoptosis, linking Cd-induced oxidative stress with SIRT1-p53 signaling (Ran et al. 2023).

Liu et al. (2016) investigated Cd-triggered cell death mechanisms in primary rat osteoblasts isolated from 18- to 19-d Sprague–Dawley fetal calvariae and cultured in DMEM + 10% FBS. Osteoblasts were exposed in vitro (submerged culture) to Cd acetate (typically 2 µM, with dose testing at 1 to 5 µM) for short durations (e.g. 3 h), and apoptosis was assessed by Bax/Bcl-2 expression, PARP cleavage, and nuclear condensation. In parallel, autophagy activation was evaluated using LC3-Il, Beclin-1, and Atg5, alongside autophagic vacuole staining. Functionally, autophagy appeared cytoprotective. Rapamycin (100 nM, 1 h pretreatment) reduced Cd-associated apoptosis, whereas chloroquine (5 µM, 30 min pretreatment) or Beclin-1 siRNA (100 nmol/L) exacerbated cytotoxicity and apoptotic outcomes (Liu et al. 2016). Collectively, these data support Cd as a driver of osteoblast loss through coordinated apoptosis/autophagy responses, providing biological plausibility for reduced bone formation capacity under Cd exposure.

#### Structural and developmental deficits (growth, microarchitecture, growth plate integrity)

##### Experimental data

Animal models reveal consistent skeletal deficits following metal exposure. In an adolescent rat model, Tomaszewska et al. (2016) exposed 6-wk-old Wistar rats (*n* = 72; *n* = 12/group) to dietary Cd + Pb for 12 wk (7 mg Cd/kg diet + 50 mg Pb/kg diet), with comparison to an unexposed control group. Relative to controls, co-exposed rats showed reduced femur growth and strength, including shorter femur length (27.3 ± 1.4 vs 36.7 ± 1.8 mm) and lower ultimate strength (104 ± 8 vs 127 ± 7 N) in the Cd + Pb (negative control) group. Densitometric analysis by pQCT further supported bone loss, with lower distal femur total mineral content (TOT B CNT: 11.94 ± 1.66 vs 16.81 ± 2.83 mg/mm) and total bone density (TOT B DEN: 783 ± 43 vs 878 ± 11 mg/c^3^) compared with controls. Finally, histology indicated compromised joint/developmental cartilage structure, with reduced articular cartilage thickness across Cd + Pb-exposed groups and altered growth plate morphology (zone-specific thickness changes), consistent with disruption of normal growth-plate integrity during adolescent skeletal development (Tomaszewska et al. 2016).

Amphibian models further show impaired ossification and shortened bone length following early-life exposure, as well as a disruption to thyroid hormone signaling. Chen et al. (2022) conducted a chronic waterborne exposure study in *Bufo gargarizans* by exposing embryos (Gosner stage 2–3) through completion of metamorphosis to Cd and Pb, alone or combined (nominal 1 µM, Cd, 1 µM Pb, or 1 µM Cd + 1 µM Pb) in dechlorinated water, with solutions replaced every other day. Measured concentrations averaged approximately 108 µg/L Cd, 204 µg/L Pb, and 106 µg/L Cd + 201 µg/L Pb (mixture). Skeletal outcomes were assessed at metamorphic climax (Gs 42) and post-metamorphosis (Gs 46) using alcian blue-alizarin red staining with bone morphometrics (e.g. vertebral column, antebrachium, ilium, femur, tibia/fibula). At Gs 42, the Cd/Pb mixture significantly reduced multiple bone lengths relative to controls, including femur (1.71 ± 0.05 vs 2.03 ± 0.10 mm) and tibia/fibula (1.51 ± 0.07 vs 1.73 ± 0.14 mm), consistent with suppressed endochondral ossification and shortened long bones. The mixture also reduced overall growth metrics (e.g. total length and snout-to-vent length) at Gs 42, supporting the conclusion that combined Cd + Pb exposure impairs normal developmental growth and skeletal formation (Chen et al. 2022).

In vivo, subchronic oral Cd exposure alters compact bone microstructure in ways consistent with early osteoporotic change. Duranova et al. (2014) randomized 1-mo-old male Wistar rats to CdCl2 in drinking water (30 mg/L) for 90 d versus controls and evaluated femoral histology/histomorphometry. Cd exposure did not significantly change femoral length or cortical thickness but increased femoral weight (1.27 ± 0.14 g vs 1.05 ± 0.17 g; *P* = 0.006) and produced resorption lacunae interpreted as an early stage of osteoporosis, alongside significantly reduced size metrics (area/perimeter/max–min diameter) of primary osteons’ vascular canals, Haversian canals, and secondary osteons, indicating reduced bone vascularization and altered microarchitecture (Duranova et al. 2014).

Exacerbating effects are seen with combined exposures, including HFD, which can intensify trabecular deterioration under Cd co-exposure. In male C57BL/6J mice (8 wk old), Zhang et al. (2020a) used a 2×2 design (control, Cd, HFD, HFD + Cd) where Cd was administered intraperitoneally as CdCl2 (1.0 mg/kg body weight) twice weekly for 20 wk, and HFD provided 60% of kcal from fat. Micro-CT analyses of the distal femur showed that while Cd or HFD alone reduced trabecular bone quality, the combined HFD + Cd group exhibited further detrimental effects, including lower trabecular BMD, lower BV/TV, reduced Tb.N and Tb.Th, and increased Tb.Sp, consistent with more severe trabecular bone loss and microarchitectural disruption than either exposure alone (Zhang et al. 2020a).

#### Epigenetic and post-transcriptional regulation (miRNAs, translation discordance, nuclear architecture)

##### Experimental data

MicroRNAs (miRNAs) are increasingly recognized as mediators of Cd skeletal toxicity. Wu et al. (2020) exposed human BM-MSCs to soluble CdCl2 in submerged culture (0, 2.5, or 5.0 µM) and evaluated early signaling changes (e.g. 24 H) alongside osteogenic differentiation endpoints (7 to 14 d). Following CdCl2 exposure, miR-143-3p increased dose-dependently, and bioinformatic prediction plus luciferase reporter testing supporting direct binding of miR-143-3p to the ARL6 3′-UTR (WT responsive; mutant not), linking Cd to post-transcriptional suppression of ARL6. Consistent with this mechanism, CdCl2 exposure was associated with reduced ARL6 and inactivation of Wnt/β-catenin pathway (decreased Wnt3a, β-catenin, LEF1, TCF1), and ARL6 overexpression blunted these pathway effects while recuing osteogenic readouts (e.g. ALP/RUNX2 expression, ALP activity, and ALP/Alizarin Red S staining) during 7- to 14-d osteogenic differentiation. Functionally, miR-143-3p inhibition mitigated CdCl2-induced suppression of Wnt/β-catenin signaling and improved osteogenic differentiation, whereas ARL6 knockdown (shRNA-ARL6) diminished this rescue, supporting a (miR-143-3p)-ARL6-Wnt/β-catenin axis as a mechanistic route by which Cd impairs osteogenesis (Wu et al. 2020).

Supporting these post-transcriptional effects, Ou et al. (2021) exposed MC3T3-E1 subclone 14 osteoblast-like cells to CdCl (5 to 20 µM; viability assessed across a wider range up to 100 µM) for ∼60 h and evaluated osteogenic regulators at both the transcript and protein levels. Cd reduced *Runx2* (and *Col-1*) mRNA expression in a dose-dependent manner and suppressed ALP activity, but Western blotting showed that Runx2 and ALP protein levels were decreased only at the highest dose (20 µM), indicating an inconsistency between transcriptional and protein-level responses. This discordance suggests that Cd may disrupt osteoblast differentiation not only through transcriptional repression but also through post-transcriptional control of protein abundance (e.g. translation or protein stability/turnover mechanisms such as ubiquitination), which could contribute to impaired mineralization and osteogenic function (Ou et al. 2021).

Structural evidence of nuclear injury, including chromatin condensation and a significant reduction in nuclear size, further supports Cd-induced cellular damage. In MSCs evaluated using Hoeschst 33342 staining to assess chromatin morphology, nuclei diameter was reduced in the Cd-exposed condition (e.g. 11.86 ± 0.15 µm in controls vs 8.9 ± 0.36 µm at 24 h, with similarly reduced values at 48 to 72 h), accompanied by marked decreases in cytoplasmic area, consistent with apoptotic-like morphological changes (Abnosi and Golami 2017).

Zhao et al. (2014) investigated how ATO and miR-204 regulate adipogenic-osteogenic balance in BM-MSCs derived from aplastic anemia (AA) patients, a setting where BM-MSCs were more prone to adipogenesis than osteogenesis. Using a submerged in vitro exposure, they treated AA BM-MSCs with 1 µM ATO for 48 h, which partially rescued osteogenic defects (larger Alizarin Red mineralization nodes) and reduced adipogenic lipid droplet accumulation of Oil Red O staining. Stain quantification was performed by redissolving Alizarin Red and reading absorbance at 595 nm (with parallel semiquantification of Oil Red O). Over the differentiation time course, *Runx2* expression was lower in AA BM-MSCs than controls and was partially restored by ATO pretreatment, consistent with improved osteogenic staining. Mechanistically, they identified *Runx2* as a direct target of miR-204. A luciferase reporter assay showed that miR-204 reduced the activity of *Runx2* 3-UTR construct and that a seed-region mutation abolished this inhibition, supporting direct binding. Functionally, miR-204 mimic/inhibitor transfection (final 50 nM, 72 h) decreased or increased Runx2 protein, respectively, and shifting miR-204 levels promoted adipogenesis while suppressing osteogenesis, although ATO did not show a clear additional effect on Runx2 once miR-204 was experimentally manipulated, suggesting these factors may act through partially overlapping or parallel control points (Zhao et al. 2014).

Arsenic also perturbs regulatory signaling and epigenetic networks. In a study of endemic fluoride-As co-exposure, Zeng et al. (2019) integrated population biomarker patterns with mechanistic osteoblast experiments to interrogate canonical WNT/CTNNB1 signaling. In the SEAF population, increasing fluoride exposure corresponded to lower serum DKK1 and higher GSK3β, β-catenin, and osteogenic differentiation indicators (*Col1a1* and *Balp*), whereas As grouping showed upregulation of Gsk3β/β-catenin and *Balp* without evidence of altered DKK1, suggesting node-specific sensitivity within Wnt signaling. Mechanistically, the authors treated hFOB 1.19 osteoblast-like cells (submerged in culture medium) with 1 mM NaF and 0.1 µM NaAsO2 for 24 h (dose/time selected based on viability). Under these conditions, *DKK1* knockdown (∼50% protein reduction) increased Wnt-pathway activation and increased COL1A1 and BALP protein in fluoride, As, and combined exposures, consistent with enhanced osteogenic differentiation when the Wnt inhibition *DKK1* is suppressed. In contrast, GSK3β knockdown (∼40% reduction; *P*-value GSK3β ∼70% reduction) reduced β-catenin signaling capacity and lowered *Col1a1/Balp* relative to siRNA controls, indicating that GSK3β (rather than *DKK1*) was required to sustain osteogenic differentiation in this co-exposure context (Zeng et al. 2019).

Mercury can localize fetal cell populations, with a marked predilection for the nuclear membrane/nuclear envelope. In the prenatal mouse model, Pamphlett et al. (2019) exposed pregnant C57 mice to 0.5 mg/m3 Hg vapor for 4 h/d over 5 d in late gestation and detected inorganic Hg by autometallography in neonatal tissues. Hg deposition was observed in the nuclear membranes of retinal ganglion cells and endothelial cells, and in the optic nerve in glial cell nuclear membranes/processes and endothelial cells (Pamphlett et al. 2019). This nuclear-envelope localization supports that Hg can promote free radical formation, autoimmune reactions, and genetic/epigenetic changes, raising concern for persistent downstream effects of early life exposure.

### Risk of bias

Risk of bias was assessed with QAROBSTR and visualized using robvis (full study-level results in Table S2). Large cohort studies (Cao et al. 2019; Qi et al. 2023; Cheng et al. 2024) were rated low risk given robust designs, validated exposure measures, and confounder adjustment. Maghbooli et al. (2018) was high risk due to small sample size, unclear representativeness, and limited confounder control. Liu et al. (2015) and Abu-Elmagd et al. (2017) were moderate risk, primarily for exposure classification and generalizability issues. Most experimental studies were low risk, with rigorous exposure definitions, validated outcomes, and full data reporting (Wang et al. 2022a, 2022b; Bhattarai et al. 2023; Ge et al. 2023; Hu et al. 2023; Song et al. 2023; Zhou et al. 2023). Moderate-risk ratings reflected gaps such as absent blinding (Papa et al. 2015; Al-Ghafari et al. 2019), limited statistical reporting (He et al. 2019; Ma et al. 2021b), or incomplete validation of molecular targets (Liu et al. 2016; Ou et al. 2021).

Although many studies are well-conducted and exhibit low bias, a considerable proportion have moderate risk, primarily driven by methodological limitations such as lack of blinding, limited outcome validation, and insufficient control of confounders. A few studies were flagged for high risk, underscoring the importance of standardized and transparent reporting practices in preclinical and in vitro research.

## Discussion

This present review integrated epidemiologic evidence with in vivo and in vitro studies that assessed related skeletal endpoints across biological levels. This approach was intended to capture a broader evidence base relevant to air pollution and bone health, recognizing that endpoints are not perfectly comparable across human and experimental systems. Despite these challenges, synthesizing population-level associations alongside experimental mechanistic evidence provided a structured way to evaluate whether findings converge on coherent skeletal hazards.

Overall, the literature included supports an association between ambient air pollution exposures, particularly PM and airborne metals, and adverse skeletal outcomes across the lifespan. Importantly, PM mass metrics (e.g. PM2.5, PM10) represent complex mixtures of particles that vary by source and location and contain multiple chemical constituents (including metals, organics, salts, and carbonaceous material) as well as surface properties that influence toxicity (EPA 2019b, 2024; World Health Organization 2025). Across the included epidemiologic studies (*n* = 9), higher ambient PM exposure was associated with lower BMD, increased fracture-related outcomes, and/or altered bone turnover markers, with the magnitude and consistency of associations varying by population, exposure metric, and outcome definition. These associations were supported by experimental evidence from animal (*n* = 21) and in vitro (*n* = 25) studies reporting disruptions in bone remodeling and osteoblast/osteoclast function. Importantly, bone remains underrepresented in environmental health research and risk assessment frameworks relative to more commonly evaluated cardiopulmonary endpoints, and the reviewed evidence suggests skeletal outcomes warrant greater attention as potential targets of pollution-related toxicity.

In this review, we treat PM as a particle/mixture class, where PM mass metrics (e.g. PM2.5, PM10) represent correlated mixtures of chemical species and particle properties rather than a single agent (EPA 2019b, 2024; World Health Organization 2025). Airborne metals are interpreted primarily as chemical constituents within PM (i.e. PM-bound components that may contribute to toxicity through redox activity and bioaccessible dissolution following inhalation), while recognizing that some epidemiologic studies also evaluate using biomarker measures that can integrate exposure across multiple routes and may not be PM-specific (Kastury et al. 2018; Andrade-Oliva et al. 2025). Accordingly, where metal-specific results are reported, we interpret them as (i) potential contributors/mediators of PM mixture toxicity when measured as PM constituents, and (ii) partially independent exposure indicators when assessed via internal biomarkers, with likely co-exposure and confounding by other mixture components.

Taken together, the WoE across human and experimental studies indicate a potential skeletal hazard of PM and airborne metal exposures (Table 4 for certainty ratings). However, inference from epidemiologic associations remains constrained by residual confounding (e.g. socioeconomic status, diet, physical activity, smoking, co-morbidities), as well as misclassification inherent to fixed-site monitoring, satellite estimates, and residential proxies that may not capture individual-level variability (time-activity patterns, indoor exposures, occupational sources). To account for differences in PM exposure assessment in the synthesis and meta-analysis (monitoring vs satellite/model-based estimates, spatial resolution, and averaging windows), we coded featured during data extraction and prioritized pooling only among studies with comparable exposure metrics and time windows, while treating cross-approach comparisons qualitatively as a source of heterogeneity. Experimental studies strengthen biological plausibility but require careful interpretation due to species differences, differences in skeletal physiology and life stage timing, and common reliance on high-dose or simplified exposure paradigms. As such, experimental literature is best interpreted as hazard-identifying, demonstrating that exposures can disrupt skeletal biology under defined conditions, rather than predictive of low-dose human risk.

Mechanistic findings are broadly consistent with an Adverse Outcome Pathway (AOP)-aligned interpretation, in which oxidative stress and inflammation function as early key events that can propagate downstream perturbations in osteogenic signaling and bone remodeling (Ankley and Edwards 2018). The AOP framework is intentionally stressor-/chemical-agnostic, serving as a structural way to organize evidence linking mechanistic events to adverse outcomes rather than attributing effects to a single agent (Ankley and Edwards 2018). As an example of a developed skeletal AOP, AOP-Wiki describes AOP 482 “Deposition of energy leading to occurrence of bone loss,” with an OECD report evaluating evidence strength (Sandhu et al. 2025a, 2025b). In this AOP, deposition of energy is the molecular initiating event, whereas oxidative stress is an early event that propagates downstream signaling changes, affecting osteoblast/osteoclast homeostasis and remodeling (Sandhu et al. 2025a). Because ambient PM and PM-associated metals are not defined by “energy deposition,” we apply AOP 482 here as a conceptual organizing scaffold (convergence on shared downstream key events such as oxidative stress and inflammation), rather than asserting an established PM-specific AOP (Sandhu et al. 2025b). Accordingly, many proposed AOP linkages in this literature remain hypothesis-generating rather than established for air pollution exposures, because key event relationships and quantitative response relationships are not consistently demonstrated across models or across realistic exposure ranges. Moreover, some early molecular responses (e.g. transient antioxidant or inflammatory signaling) may be adaptive at low or short-term exposure levels, whereas persistent or high-magnitude perturbations are more plausibly linked to downstream adverse outcomes, such as impaired mineralization, altered bone architecture, or reduced BMD. In interpreting experimental findings within this causal framework, we do not treat isolated changes in stress markers (e.g. ROS, cytokines, or short-term pathway activation) as inherently adverse, because such responses can fall within normal biological variability or represent compensatory adaptation. Instead, we interpret these early responses as potentially adverse key events when they are sustained and accompanied by functional impairment in osteogenic or remodeling endpoints (e.g. reduced differentiation capacity, ALP activity, matrix mineralization, altered osteoclastogenesis/resorption signaling) and/or when they plausibly connect to tissue-level outcomes (e.g. microarchitecture changes or reduced BMD) (Ankley and Edwards 2018; Sandhu et al. 2025a). In studies that report molecular changes without downstream functional or structural endpoints, we describe these findings as mechanistic signals of biological perturbation rather than evidence of adversity. Therefore, when discussing AOP-like cascades, it is important to distinguish early compensatory responses from events that plausibly contribute to skeletal adversity.

### Absorption, distribution, metabolism, and excretion of PM- and heavy metal-embedded air pollution

**Fig. 4.**
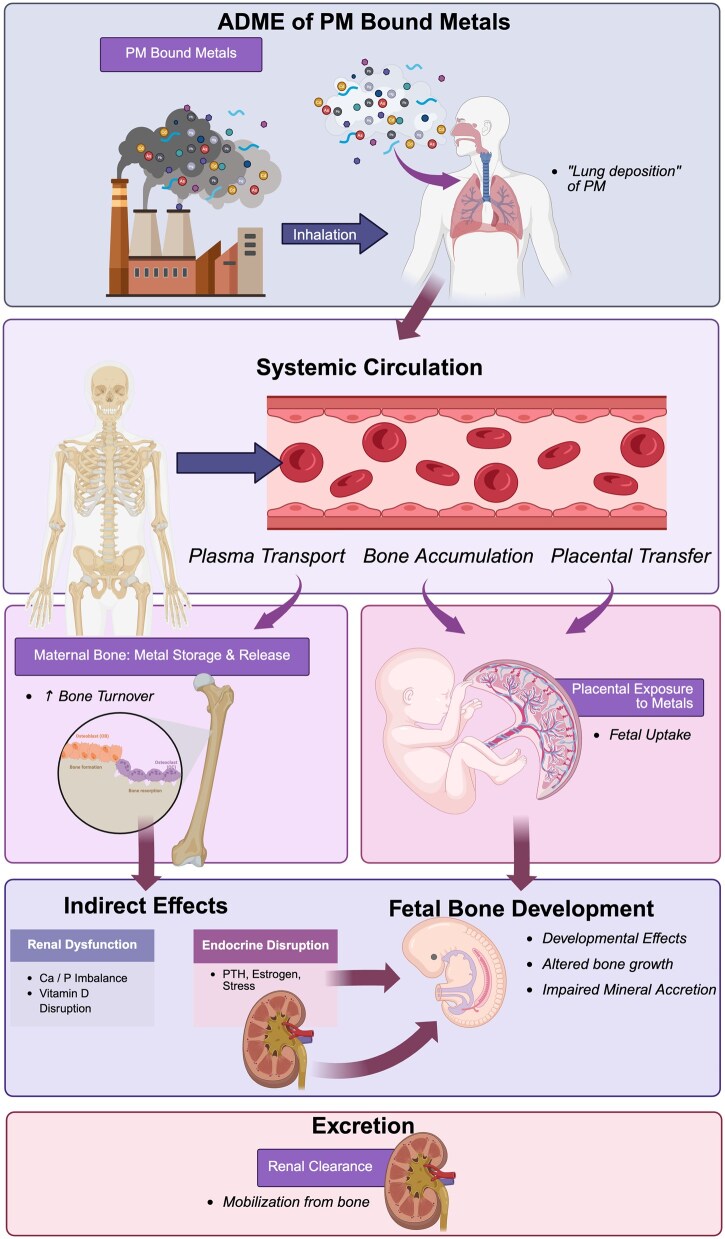
Proposed ADME mechanisms for heavy metal-bound PM and its systemic impact.

PM2.5 is well known to act as a carrier for heavy metals, as metals readily adsorb onto particle surfaces (Li et al. 2022). This effect becomes even more pronounced as particle size decreases, as ultrafine and nanosized particles have a much larger surface area relative to their mass, allowing greater deposition of contaminants such as Pb, Cd, and other trace metals (Kumari et al. 2021). Consistent with this, a study by Gao et al. (2023) found that heavy metals, including Cd, Pb, and others, were much more highly enriched in smaller particles (PM2.5) compared with larger particles (PM10). A general hypothesized schematic of heavy metal-bound PM and its absorption, distribution, metabolism, and excretion (ADME) can be seen in Fig. 4.

Health risk studies have further demonstrated that biological relevance of PM2.5-bound metals. In a study by Li et al. (2022), children were found to experience higher overall risk values from PM2.5-associated heavy metals than adults. Chromium (Cr), As, and Pb were the primary contributors to noncarcinogenic risk, whereas Cr(VI), As, and Cd contributed most strongly to carcinogenic risk, highlighting both particle size-dependent exposure and age-related susceptibility (Li et al. 2022).

Once inhaled, particle size strongly influences where metal-bearing PM deposits within the respiratory tract and ultimately the bioavailability of these metals. As particles travel during inhalation, their trajectories diverge depending on size and momentum, with heavier particles more likely to depart from the airflow at regions of curvature or branching in the airways. Coarse particles (∼5 to 30 µm) are primarily deposited in the nasopharyngeal region through inertial impaction. Smaller particles (≤ 5 µm) that evade upper airway capture are deposited within the tracheobronchial tree, mainly through sedimentation, and may subsequently be cleared via mucociliary transport. By contrast, fine and ultrafine particles (≤ 1 µm), including nanoparticles (≤ 100 nm), can reach the alveolar region, where particle removal is markedly slower and conditions favor prolonged residence and enhanced potential for metal release (Bakand et al. 2012).

Once at the alveolar level, ultrafine and nanosized particles come into close contact with the alveolar epithelium, increasing the likelihood of systemic uptake. These particles, or the metal ions released from them, can cross the air–blood barrier via transcytosis, paracellular pathways, or macrophage-mediated transport. As a result, inhaled metal-bearing PM because bioavailable and enters systemic circulation (Bakand et al. 2012).

Once in the bloodstream, heavy metals are transported predominantly bound to plasma proteins such as albumin, metallothionein (MT), and other ligands (Thévenod and Lee 2024). Through systemic distribution, these metals reach mineralized tissues, particularly bone, where they accumulate due to their affinity for hydroxyapatite and their ability to substitute for calcium through chemical mimicry (Wopenka and Pasteris 2005). This results in long-term sequestration of metals within the skeleton.

Importantly, the skeleton is not a uniform compartment. Trabecular bone has a turnover rate that is approximately 3 to 10 times higher than that of cortical bone (ICRP 2012), and metals such as Pb exhibit faster exchange rates in trabecular bone (Silbergeld et al. 1988). As a result, bone acts not only as a storage site but also as a dynamic reservoir, with metals being released back into circulation during periods of increased bone resorption (Silbergeld et al. 1988). Physiological and pathological conditions that alter bone turnover, including pregnancy, lactation, menopause, and osteoporosis, can therefore lead to increased mobilization of Pb and other metals, creating chronic low-level systemic exposure and increasing risk during sensitive life stages, such as fetal development (Berglund et al. 2000; Tsaih et al. 2001).

With respect to skeletal toxicity, inhaled metal exposure can affect bone through both direct and indirect mechanisms. Direct effects occur when metals are deposited in bone and interact with bone cells, where they can impair osteoblast differentiation and function, enhance osteoclast activity, and disrupt bone remodeling at the cellular level. Indirect effects arise through metal-induced dysfunction in other target organ systems, particularly the kidney and endocrine system (Silbergeld et al. 1988). For instance, bone interacts with the central nervous system and stress/inflammatory signaling (bone–brain axis), in part through bone-derived hormones such as osteocalcin that act on the brain and influence neurodevelopment and cognition (Oury et al. 2013). Renal impairment can alter calcium and phosphate homeostasis and vitamin D metabolism (bone–kidney axis), through a tightly coupled mineral homeostasis pathway that underlines the CKD-MBD framework (KDIGO 2017 Clinical Practice Guideline Update for the Diagnosis, Evaluation, Prevention, and Treatment of Chronic Kidney Disease–Mineral and Bone Disorder (CKD-MBD) 2017). Meanwhile, endocrine disruption, such as interference with estrogen or parathyroid hormone signaling, can further exacerbate bone loss (Park et al. 2024). Together, these pathways link inhalation exposure to PM-bound metals with direct bone-cell toxicity (direct osteoblast/osteoclast impact) and indirect, systemic mechanisms across interconnected organ pathways that regulate mineral balance, growth, and developmental programming that ultimately compromise skeletal integrity.

### Potential mechanisms to be explored further

Across experimental studies (animal *n* = 21), in vitro (*n* = 25), ROS generation frequently emerged as an early cellular response to PM exposures and PM-associated constituents. Multiple studies suggest that oxidative stress and inflammatory signaling can contribute to osteoblast dysfunction, altered osteogenic differentiation, and/or shifts in osteoclast activity (Brzóska et al. 2016; He et al. 2019; Liang et al. 2025). Consistent with an AOP-aligned interpretation, we treat these stress responses as mechanistically informative but not necessarily adverse unless accompanied by impaired osteogenic function (e.g. differentiation/mineralization deficits) or remodeling imbalance endpoints. For example, Abu-Elmagd et al. (2017) reported increased inflammatory markers (e.g. *TNF-α, IL-6*) in BM-MSCs exposed to PM under in vitro conditions, with higher doses linked to reduced proliferation and increased cell death. Similarly, Cui et al. (2015) reported increased intracellular ROS, quantified in bone marrow cells using a FITC-based ROS detection reagent (flow cytometry), alongside reduced Akt phosphorylation, consistent with ROS-linked (partially Akt-mediated) inhibition of PI3K/Akt signaling and reduced proliferation. In support of a ROS-dependent mechanism, these effects were attenuated by antioxidant/ROS-suppression approaches, including N-acetylcysteine treatment and a transgenic antioxidant network model overexpressing *SOD1, SOD3*, and *GPx1* (Cui et al. 2015). Depending on exposure context, downstream fate processes may involve apoptosis, senescence, autophagy disruption, or other stress responses (Zhao et al. 2015; Al-Ghafari et al. 2019; He et al. 2019; Luo et al. 2021; Hammad et al. 2023; Song et al. 2023).

Epidemiological findings (*n* = 9) are broadly consistent with these mechanisms, in that chronic PM exposure has been associated with lower BMD and fracture-related outcomes in some populations. Notably, because epidemiologic studies operationalize “exposure” using different averaging windows (e.g. annual or multi-year means vs shorter pregnancy windows), the implied biological timescale of exposure differs across studies, which may influence how directly observed associations reflect time-sensitive pathways such as oxidative stress and inflammation. However, mechanistic interpretation from human studies remains indirect, because systemic biomarkers of inflammation or oxidative stress are influenced by many co-exposures and behaviors, and exposure misclassification can attenuate or distort exposure-response patterns. Additionally, dose–response relationships may be nonlinear, with threshold-like behavior, saturation, or hormetic responses possibly depending on composition and susceptibility. This reinforces the need for future studies that link realistic exposure levels and internal dose measures to mechanistic endpoints.

Together, the evidence base indicates that oxidative stress, inflammation, and altered survival/osteogenic signaling are plausible contributors to skeletal effects, but stronger mechanistic integration will require harmonized endpoints, mixture-aware designs, and exposure characterization that supports cross-study comparability.

### Developmentally vulnerable windows sensitive to exposure

Critical windows for skeletal development span prenatal ossification and postnatal mineral accrual. In humans, major skeletal patterning and primary ossification occur during early-mid gestation, whereas rapid fetal growth and mineralization accelerate later in pregnancy. After birth, infancy through adolescence represents a second critical window as bone mass accrual and remodeling determines peak bone mass. In this context, trimester-specific PM and metal associations are most informative when aligned to developmental processes (e.g. early gestation patterning vs late gestation mineralization), and experimental evidence is strongest when exposure windows are tied to well-defined periods of osteogenesis and mineral accrual in the model organism.

Beyond skeletal-specific endpoints, a broader pregnancy and developmental literature consistently associate maternal PM exposure with general markers of impaired growth and developmental risk, including reduced birth weight and fetal growth restriction, higher risk of hypertensive disorders of pregnancy (including preeclampsia), and adverse birth outcomes (e.g. preterm birth) (Stieb et al. 2012; Dadvand et al. 2013; Wu et al. 2016; Bekkar et al. 2020). These outcomes are relevant to skeletal development because they reflect systemic placental and maternal–fetal perturbations (e.g. inflammation, oxidative stress, vascular dysfunction) that can secondarily influence fetal nutrient delivery, endocrine signaling, and mineral accrual, processes tightly coupled to bone growth trajectories. Maternal metabolic dysregulation, including gestational diabetes and hyperglycemia, has also been linked to altered fetal growth patterns and may interact with pollution-related inflammatory stress, representing a plausible susceptibility modifier for offspring skeletal programming (Hu et al. 2020; Kukreti and Subashini 2021).

Developmental bone formation may be particularly sensitive to environmental insults during fetal life and early postnatal life. Across the included epidemiologic studies of pregnancy and fetal growth (*n* = 2) maternal PM exposure was associated with fetal growth measures that include skeletal-related metrics (e.g. femur length), through findings varied by study design, exposure window, and covariate adjustment (Cao et al. 2019). Where trimester-specific estimates were available (*n* = 2), associations were most frequently reported for timed exposures in relation to skeletal metrics, supporting the interpretation that timing of exposure may modify vulnerability of the developing skeleton (Cao et al. 2019; Bhattarai et al. 2020). In synthesizing developmental findings, we treated trimester-specific and pregnancy-averaged exposure estimates as distinct exposure constructs rather than directly interchangeable measures, and we only combined estimates quantitatively when exposure window definitions were aligned. Experimental evidence (animal *n* = 21; in vitro *n* = 25) suggests that PM and related constituents can trigger oxidative stress and inflammatory responses at the maternal–fetal interface and/or within developing tissues, which could plausibly alter skeletal growth trajectories (Bhattarai et al. 2020). In experimental models, the most relevant developmental windows include gestational exposures during ossification and early postnatal periods of rapid bone growth. However, the extent to which these findings translate to human developmental risk remains uncertain due to differences in placental biology across species, exposure routes and dose, and the challenge of isolating skeletal effects from broader developmental outcomes.

Toxicokinetic factors may further amplify early-life susceptibility. During pregnancy, placental transfer and maternal–fetal partitioning can increase fetal internal exposure to circulating pollutants and PM-associated constituents, even when maternal exposures are within typical ambient ranges. In early postnatal life, developmental toxicokinetics, particularly immature renal clearance (lower glomerular filtration and evolving tubular handling) and age-dependent distribution, can prolong effective half-lives and increase internal dose per unit exposure in neonates and infants (Iacobelli and Guignard, 2021; Smeets et al. 2022). For metals that accumulate in maternal tissues (including bone), pregnancy and lactation can mobilize stored burdens and contribute to fetal and infant internal dose, complicating interpretation of external exposure metrics alone (Gulson et al. 1997, 2003; Téllez-Rojo et al. 2004). If relevant to susceptibility framing, early-life hemodynamic vulnerability (e.g. volume status-related reductions in renal perfusion, “pre-renal” physiology) may further modify elimination capacity and susceptibility during periods of rapid skeletal development (Andreoli 2009; Pandey et al. 2017).

Disruption of mineral homeostasis represents another plausible vulnerability pathway. Altered calcium–phosphorus metabolism, vitamin D signaling, and endocrine regulators (e.g. PTH, FGF23) can influence mineralization and bone turnover, and some evidence suggests these systems may be perturbed in exposure contexts linked to air pollution or metal co-exposures (Sun et al. 2020). Still human evidence is limited by confounding (diet, supplementation, sunlight exposure, kidney function) and measurement variability, and experimental findings require careful dose extrapolation.

A key limitation is that many studies report pregnancy-averaged exposures or broad developmental periods without mapping exposure timing to specific milestones of ossification and mineral accrual, limiting inference about which developmental windows are most sensitive. Overall, developmental literature supports heightened susceptibility as a plausible concern, but stronger longitudinal designs are needed to determine whether prenatal or early-life exposures produce persistent skeletal impacts (e.g. peak bone mass deficits) and to identify exposure windows most strongly linked to adverse outcomes (Tong et al. 2023).

### Heavy metals may alter bone homeostasis through oxidative and cellular pathways

Airborne heavy metals (e.g. Pb, Cd, Hg, As) are common constituents of PM and often co-occur with other toxic components, making it difficult to fully disentangle single-metal effects from broader mixture content. Because metal exposures were assessed using both PM-speciation measures (PM-bound constituents) and internal biomarkers (e.g. blood/urine), we did not assume these measures represent the same exposure pathway, and we synthesized them separately to avoid conflating PM-bound metal toxicity with multi-route cumulative body burden. In this review, we interpret metal-related findings primarily as effects of PM-associated constituents (i.e. metals carried on particles that may become bioavailable through dissolution and redox activity after inhalation), rather than as wholly independent exposures. However, we note that some epidemiologic studies assess metals using internal biomarkers (e.g. blood or urine), which can reflect multi-route exposure and cumulative body burden and therefore may not be specific to PM-bound uptake. Across experimental studies (animal *n* = 21, in vitro *n* = 25), multiple metals were associated with oxidative stress, mitochondrial dysfunction, inflammatory signaling, and altered osteogenic differentiation and/or osteoclast activity. These effects provide mechanistic plausibility for skeletal toxicity, but they remain primarily hazard-identifying findings, because exposure levels, routes, and bioavailability in experimental systems may not reflect typical ambient conditions.

Lead exposure has been linked to impaired osteoblast differentiation and altered osteogenic signaling in experimental systems, and epidemiologic studies have reported associations between Pb biomarkers and bone outcomes, through confounding and reverse causation (bone as a metal reservoir) remain important considerations (Beier et al. 2015; Rafiei et al. 2018). Cadmium has been associated with impaired osteogenic pathways and increased osteoclastogenic signaling in several experimental studies, with epidemiological evidence variably linking Cd biomarkers to BMD or fracture-related outcomes depending on population and exposure distribution (Al-Ghafari et al. 2019; Wu et al. 2019, 2020; Ma et al. 2021a; Hu et al. 2023; Ran et al. 2023; Tong et al. 2023; Wan et al. 2023). Mercury evidence in the skeletal context remains comparatively limited and inconsistent among epidemiologic studies. As studies suggest potential remodeling disruption through oxidative and signaling pathways, though exposure assessment and co-exposure patterns complicate interpretation.

Because PM is a mixture, observed “metal-associated” effects may reflect mixture interactions (co-pollutants, particle chemistry, and correlated exposures). Although synergistic effects are hypothesized and biologically plausible, the current evidence base does not consistently demonstrate synergism in a way that supports firm conclusions. Mixture-aware study designs, both epidemiological and experimental, are needed to determine whether combined exposures act additively, antagonistically, or synergistically across relevant dose ranges.

### Skeletal endpoints used to link PM exposure with bone-related phenotypes

Across the studies and supporting evidence reviewed, skeletal imaging and circulating biomarker readouts were commonly used as endpoints to support bone as a potential target tissue of PM-related toxicity (Farhi et al. 2014; Wu et al. 2014; Liu et al. 2015; Lim et al. 2016; Tomaszewska et al. 2016; Hu et al. 2020; Kumari 2021; Li et al. 2022; Torres-Rodríguez et al. 2022; Wang et al. 2022c; Qi et al. 2023; Yang et al. 2023; Cheng et al. 2024; Qu et al. 2025; Scheepers et al. 2025). In contrast to clinical practice, where measures such as DXA/DEXA-derived BMD and bone turnover markers are typically ordered in response to symptoms or established risk factors. These same tools function in environmental health research as interpretable, cross-study endpoints that can help connect population-level associations with mechanistic evidence.

When appropriately implemented, imaging and bone turnover biomarkers provide several observable advantages in this literature: (i) standardized, comparable outcomes across cohorts (e.g. BMD via DXA/DEXA), (ii) sensitivity to susceptible life stages and subpopulations, and (iii) linkage of exposures to intermediate phenotypes (e.g. bone turnover) that may precede overt clinical outcomes. Their interpretability depends strongly on alignment with exposure assessment, as differences in spatial resolution and averaging windows can shift “exposure” from longer-term background burden to shorter susceptibility periods.

Taken together, these endpoints offer translational value for integrating epidemiologic and experimental findings, particularly in pregnancy and early life, when clinical skeletal outcomes may not be observable until later. However, their feasibility and appropriateness remain study-dependent, shaped by design, ethical considerations, and participant burden. Accordingly, they are best framed as context-specific research translation endpoints rather than universal clinical or policy recommendations.

### Limitations of current evidence

Despite growing interest, several limitations constrain confidence in specific causal interpretations of this work. Most epidemiologic studies are observational and remain vulnerable to residual confounding (health behaviors, socioeconomic factors, nutrition, co-morbidities) and exposure misclassification stemming from proxy-based exposure estimation and limited personal monitoring. Study heterogeneity in exposure metrics, outcome definitions, and covariate adjustment further complicates synthesis and may contribute to variability in results.

Exposure assessment heterogeneity was a major driver of between-study variability and constrained quantitative pooling. Studies differed in whether PM was estimated via fixed-site monitoring, satellite/model-based surfaces, or hybrid approaches, and they also varied in spatial resolution and averaging windows (e.g. annual vs multi-year means; trimester-specific pregnancy windows). To avoid directly implying direct comparability across nonequivalent exposure constructs, we retained these attributes in the evidence synthesis and limited meta-analytic pooling to subsets with comparable exposure definitions and time windows, treating remaining differences as key contributors to heterogeneity and potential exposure misclassification.

Experimental studies provide valuable mechanistic insights but are not inherently predictive of human risk. Many studies employ exposure levels or simplified compositions that may exceed typical ambient conditions or fail to capture real-world variability, and translation is limited by species differences, life-stage alignment, and dose extrapolation challenges. Importantly, high-dose effects observed in controlled systems do not necessarily imply proportional effects at lower doses, and nonlinear dose–response relationships may occur depending on pollutant composition, susceptibility, and compensatory biology. Additionally, developmental toxicokinetics (placental transfer, age-dependent distribution, and immature renal clearance) means that the same ambient exposure can correspond to different internal doses across life stages, adding uncertainty to cross-species translation and high-to-low dose extrapolation (Iacobelli and Guignard, 2021; Smeets et al. 2022).

*Mixture complexity remains a central challenge.* Air pollution is not a single agent, yet many studies focus on individual pollutants in isolation. Although mixture interactions are biologically plausible, current designs rarely allow robust interference regarding synergism versus additivity, and advanced mixture modeling remains underutilized. Mechanistic integration is also limited by the relatively sparse application of multi-omics or systems approaches that could better connect early molecular events to downstream skeletal endpoints.

Using an OHAT-style framing, the overall body of evidence supports the conclusion that PM and PM-associated metal exposures pose a potential hazard for adverse skeletal outcomes, with the strongest coherence emerging from convergence between epidemiologic associations (*n* = 9) and mechanistic/experimental findings (animal *n* = 21, in vitro *n* = 25). At the same time, interpretation is constrained by residual confounding and exposure misclassification in human studies and by species differences and dose extrapolation limitations in experimental systems. Thus, this review does not establish precise low-dose human risk estimates. Developmental periods remain a key concern, as fetal and early-life skeletal development may be more susceptible to oxidative and inflammatory perturbation and to disruptions in mineral homeostasis, potentially influencing long-term bone trajectories. Key knowledge gaps include improved exposure characterization (including personal monitoring and internal dose measures), mixture-aware designs that test combined pollutant effects across realistic dose ranges, and longitudinal studies that track prenatal and pediatric exposure windows to later-life skeletal outcomes. Additional gaps include clearer reporting and harmonization of exposure estimation method (monitor vs satellite/model), spatial resolution, and averaging windows, along with validation efforts that quantify measurement error so estimates from different assessment approaches can be compared more defensibly. Addressing these gaps will improve confidence in mechanistic interpretation and strengthen the evidence base for understanding air pollution as a potential contributor to skeletal health disparities across the life course.

## Conclusions

This systematic review, conducted using the OHAT framework, concludes that prenatal and early-life exposure to airborne contaminants, in particular PM2.5 and heavy metals such as lead, cadmium, arsenic, and mercury, may be suspected of being a hazard to humans for adverse effects on bone development, growth, and homeostasis (OHAT category 3). Furthermore, this hazard conclusion draws from limited moderate-confidence human evidence for increased fracture risk, musculoskeletal disorders, congenital malformations, and impaired fetal bone growth (e.g. Cheng et al., Qi et al., Cao et al., Farhi et al.), alongside mostly low-to-moderate confidence experimental findings for bone loss, impaired osteoblast differentiation, altered microarchitecture, and enhanced osteoclast activity across 10 to 20+ studies per outcome. Overall certainty was rated low to moderate due to residual confounding, exposure misclassification, and heterogeneity across studies. Mechanistically, these effects appear to be driven by oxidative stress, inflammation, Wnt/β-catenin disruption, and epigenetic changes, though human evidence remains unclear. Early-life exposure, including in utero, represents a particularly sensitive window with potential long-term consequences, as hinted by associations with congenital skeletal abnormalities and disrupted osteogenesis. Despite consistent findings, current literature is limited by a lack of mechanistic human studies and insufficient data on combined exposures. Future research should investigate mixture effects and clarify whether skeletal impacts are reversible or progressive. Overall, these findings support the inclusion of skeletal health in environmental risk assessments and underscore the importance of reducing airborne toxicant exposure to potentially protect developing and adult bone health.

## Supplementary Material

kfag067_Supplementary_Data

## Data Availability

All data generated or analyzed in this systematic review are derived from previously published studies that are publicly available through PubMed, Google Scholar, and other academic databases. The full search strategy, inclusion criteria, and extracted datasets are provided in the Supplementary Materials. No new primary data were generated for this work.
