# Supplementary material for: Airborne contaminant exposure and bone development: a systematic review and meta-analysis
Source: Toxicol Sci. 2026 Jun 2;209(6):kfag067. doi: 10.1093/toxsci/kfag067 (PMC13283646; doi:10.1093/toxsci/kfag067)
Supplement: kfag067_Supplementary_Data [file kfag067_supplementary_data.zip › Appendix_A._Supplemental_Materials_260130-cnp.docx]

Supporting Information

Airborne contaminant exposure and bone development: A systematic review

Madeline K.M. Vera-Colón^a,b^, Ruth M. Meletz^a,b^, Celina N. Phillipson^a,b^, Nicole R.L. Sparks^a,b^

^a^ Environmental Health Sciences Graduate Program, University of California Irvine, Irvine, CA, 92697, United States

^b^ Department of Occupational and Environmental Health, Joe C. Wen School of Population & Public Health, University of California Irvine, Irvine, CA, 92697, United States

**Table S1. Statistical analysis of reported ratio outcomes.**

|  |  | Hazards Ratio | Full Model (mean) | Odds Ratio | 95% Confidence Interval |
| --- | --- | --- | --- | --- | --- |
| Cheng et al. (2024) | PM2.5 Degenerative joint disease | 1.054 |  |  | 1.036, 1.072 |
|  | PM2.5 Dorsopathies | 1.11 |  |  | 1.085, 1.137 |
|  | PM2.5 Osteoporosis | 1.079 |  |  | 1.035, 1.125 |
|  | PM10 Dorsopathies | 1.05 |  |  |  |
|  | PM10 Osteoporosis | 1.032 |  |  |  |
|  | Total musculoskeletal disorders | 1.021 |  |  |  |
| Qi et al. (2023) | PM2.5 Fracture Risk | 1.70 |  |  | 1.42, 2.03 |
|  | PM10 Fracture Risk | 1.10 |  |  | 1.00, 1.21 |
|  | Composite Pollution Score | 1.03 |  |  | 1.02, 1.03 |
| Liu et al. (2015) | PM2.5 - 10 |  | 32.3 |  | 6.1, 58.5 |
| Cao et al. (2019) | Abdominal Circumference |  | -5.48 |  | -9.06, -1.91 |
|  | Femur Length |  | -5.57 |  | -6.66, -4.47 |
|  | Biparietal Diameter |  | -5.47 |  | -6.39, -4.55 |
|  | Fetal Weight |  | -14.49 |  | -16.05, -13.49 |
|  | Head Circumference |  | -13.56 |  | -14.71, -12.5 |
| Farhi et al. (2014) | Congenital Anomaly Risk |  |  | 1.06 | 1.01, 1.11 |

**Table S2. Risk of Bias Assessment Across Epidemiological and Experimental Evidence Evaluating Air Pollution (PM and heavy metals) and Bone-related Outcomes.**

| **Study** | **Selection Bias** | **Confounding** | | **Measurement Bias** | **Missing Data** | **Reporting Bias** | **Overall RoB** |
| --- | --- | --- | --- | --- | --- | --- | --- |
| Abu-Elmagd et al. (2017) | Moderate: Dose ranges well-defined but not randomized | N/A | Low | | Low | Moderate: Limited validation | Moderate |
| Al-Ghafari et al. (2019) | Moderate: Design with unclear exposure/replicates | N/A | Moderate: Limited validation | | Low | Moderate: Statistical variability | Moderate |
| Beier et al. (2015) | Moderate: Zebrafish models with unclear randomization | N/A | Moderate: Behavior without blinding | | Low | Moderate: No validation of targets | Moderate |
| Beier et al. (2017) | Low | N/A | Low | | Low | Low | Low |
| Bhattarai et al. (2023) | Low | N/A | Low | | Low | Low | Low |
| Brzoska et al. (2016) | Low | N/A | Low | | Low | Low | Low |
| Buha et al. (2019) | Moderate: Unclear randomization; sex-specific effects not controlled | Moderate | Moderate: Cross-species and matrix differences in biomarkers | | Low | Moderate: Lacks blinding and statistical transparency | Moderate |
| Cao et al. (2019) | Low | Low | Low | | Low | Low | Low |
| Chen et al. (2022) | Low | N/A | Low | | Low | Low | Low |
| Cheng et al. (2024) | Low | Low | Low | | Low | Low | Low |
| Duranova et al. (2014) | Low | N/A | Low | | Low | Low | Low |
| Farhi et al. (2014) | Low | Low | Moderate: Modeled trimester exposures | | Low | Low | Low to Moderate |
| Ge et al. (2023) | Low | N/A | Low | | Low | Low | Low |
| He et al. (2019) | Moderate: No mention of randomization/ blinding | N/A | Moderate: No technical replicates or blinding reported | | Low | Moderate: Lacks statistical detail | Moderate |
| Hu et al. (2023) | Low | N/A | Low | | Low | Low | Low |
| Li et al. (2021) | Low | N/A | Low | | Low | Low | Low |
| Liao et al. (2017) | Moderate: Limited detail on dose selection | N/A | Moderate: Unclear if blinded quantification | | Low | Moderate: Lacks detail on analytical steps | Moderate |
| Lim et al. (2016) | Low | Moderate: no control for dietary or genetics | Low | | Low | Moderate: Limited causal inference | Moderate |
| Liu et al. (2015) | Moderate: Regional variation may affect generalizability | Moderate | Low | | Low | Low | Moderate |
| Liu et al. (2016) | Moderate: Mouse model used, but lacks detail on randomization | N/A | Moderate: Mechanistic endpoints unverified | | Low | Moderate: Some interpretive overreach | Moderate |
| Liu et al. (2019) | Moderate: No randomization, unclear blinding | N/A | Moderate: Quantification and replication unclear | | Low | Moderate: Pathway analysis undeveloped | Moderate |
| Liu et al. (2020) | Low | N/A | Low | | Low | Low | Low |
| Liu et al. (2024) | Low | N/A | Low | | Low | Low | Low |
| Luo et al. (2021) | Low | N/A | Low | | Low | Low | Low |
| Lv et al. (2019) | Moderate: Unclear if group allocation was randomized | N/A | Low | | Low | Moderate: Lacked blinding and mechanistic validations | Moderate |
| Ma et al. (2021a) | Low | N/A | Low | | Low | Low | Low |
| Ma et al. (2021b) | Low | N/A | Moderate: Some endpoints lack validation | | Low | Moderate: Mechanistic causality inferred | Moderate |
| Maghbooli et al. (2018) | High: Small sample, unclear representativeness | High: Unclear control for confounders | Moderate: Pollution exposure estimates may be imprecise | | Low | Moderate: Outcomes focused on 1 marker | High |
| Ou et al. (2021) | Moderate: Limited details on replicates and randomization | N/A | Moderate: Lacked blinding | | Low | Moderate: Inconsistencies with mRNA/protein | Moderate |
| Pamphlett et al. (2019) | Moderate: Small sample size, unclear randomization | N/A | Moderate: No blinding or technical replicates reported | | Low | Moderate: Limited detail on all statistical comparisons | Moderate |
| Papa et al. (2015) | Moderate: randomization not discussed | N/A | Moderate: Some endpoints lack blinding or independent replication | | Low | Moderate: Mechanistic pathways inferred without causal testing | Moderate |
| Qi et al. (2023) | Low | Low | Low | | Low | Low | Low |
| Rafiei et al. (2018) | Moderate: No randomization reported | N/A | Moderate: No protein or phenotypic corroboration | | Low | Moderate: Lacks validation & transparency of replicates | Moderate |
| Ran et al. (2023) | Low | N/A | Low | | Low | Low | Low |
| Song et al. (2023) | Low | N/A | Low | | Low | Low | Low |
| Taha et al. (2018) | Moderate: No randomization; exposure misclassification possible | Moderate: Residual confounding likely | Moderate: No blinding reported | | Low | Moderate: Mechanistic interpretation overstated | Moderate |
| Tang et al. (2022) | Low | Moderate: limited by cross-sectional | Low | | Low | Moderate: Cannot infer causality; findings speculative | Moderate |
| Tomaszewska et al (2016) | Moderate: Unclear randomization and potential for selection bias | N/A | Moderate: Histology blinding not reported | | Low | Moderate: Selective emphasis on protective effects | Moderate |
| Tong et al. (2023) | Low | N/A | Low | | Low | Low | Low |
| Torres-Rodriguez et al. (2022) | Moderate: No detail on randomization or group allocation | N/A | Moderate: Unclear if blinding was used | | Low | Moderate: Limited mechanistic analysis | Moderate |
| Wan et al. (2023) | Low | Moderate: No dietary/ genetic covariates | Low | | Low | Low | Low to Moderate |
| Wang et al. (2022a) | Low | N/A | Low | | Low | Low | Low |
| Wang et al. (2022b) | Moderate: Unclear detail on exposure duration and recruitment | Moderate | Moderate: Bone outcomes only assessed via BMD without mechanistic endpoints | | Low | Moderate: Limited mechanistic insight; reliance on cross-sectional associations | Moderate |
| Wang et al. (2022c) | Low | N/A | Low | | Low | Low | Low |
| Wu et al. (2014) | Moderate: Lacks randomization or control detail | Moderate: Limited info | Moderate: Unclear blinding | | Moderate: Sample size not reported | Moderate Incomplete description of exposure/analysis | Moderate |
| Wu et al.  (2019) | Low | N/A | Low | | Low | Low | Low |
| Wu et al. (2020) | Low | N/A | Low | | Low | Low | Low |
| Xu et al. (2014) | Moderate: No randomization or blinding | N/A | Moderate: No validations | | Low | Moderate: Limited controls/reps | Moderate |
| Zeng et al. (2019) | Low | Moderate: Cross-sectional design limits causality | Low | | Low | Moderate: Lack of adjustment for all potential confounders | Moderate |
| Zhang et al. (2020) | Moderate: Limited detail on randomization | N/A | Moderate: No functional/protein-level validation | | Low | Moderate: Lacks pathway dissection | Moderate |
| Zhao et al. (2014) | Moderate: No mention of randomization | N/A | Low | | Low | Moderate: No detail on reps/normalization | Moderate |
| Zhao et al. (2015) | Low | N/A | Low | | Low | Low | Low |
| Zhou et al. (2023) | Low | N/A | Low | | Low | Low | Low |
