# Supplementary material for: Airborne contaminant exposure and bone development: a systematic review and meta-analysis
Source: Toxicol Sci. 2026 Jun 2;209(6):kfag067. doi: 10.1093/toxsci/kfag067 (PMC13283646; doi:10.1093/toxsci/kfag067)
Supplement: kfag067_Supplementary_Data [file kfag067_supplementary_data.zip › Appendix_B.Supplementary_Materials.pdf]

Airborne contaminant exposure and bone development: a systematic review and meta-analysis

Nicole Sparks, Madeline Vera-Colon, Ruth Meletz

Citation
Nicole Sparks, Madeline Vera-Colon, Ruth Meletz. Airborne contaminant exposure and bone development: a systematic review and meta-analysis. Not yet published.

PREVIEW

REVIEW TITLE AND BASIC DETAILS

Review title
Airborne contaminant exposure and bone development: a systematic review and meta-analysis

Condition or domain being studied
Bone development and skeletal health, including bone mineral density, bone microarchitecture, bone turnover, fractures, musculoskeletal disease, and developmental skeletal outcomes. The review examines how airborne contaminants, particularly particulate matter (PM2.5 and PM10) and airborne heavy metals (Pb, Cd, As, Hg), impact skeletal health across the lifespan.

Rationale for the review
Airborne particulate matter and associated heavy metals are well-established systemic toxicants, but their effects on bone development and skeletal homeostasis remain underrecognized. Evidence from epidemiological, in vivo, and in vitro studies suggests potential links between exposure to PM and heavy metals and adverse skeletal outcomes, including reduced bone mineral density, altered bone microarchitecture, impaired osteogenesis, developmental deficits, and increased fracture risk. No previous systematic review has comprehensively integrated human, animal, and mechanistic evidence on airborne contaminants and bone health. A systematic and transparent evaluation is needed to inform environmental health assessment and identify gaps in current knowledge.

Review objectives
To systematically identify, evaluate, and synthesize evidence from human, animal, and in vitro studies assessing the effects of airborne contaminants, specifically PM2.5, PM10, and airborne heavy metals (lead, cadmium, arsenic, mercury), on bone development and skeletal health outcomes. Objectives include: (1) determining whether airborne contaminants adversely affect bone mass, microarchitecture, turnover, developmental skeletal outcomes, or fracture risk; (2) integrating epidemiological, experimental, and mechanistic evidence; and (3) conducting meta-analyses for specific bone-related outcomes where data permit.

Keywords
Air pollution; Particulate matter; PM2.5; PM10; Heavy metals; Lead; Cadmium; Arsenic; Mercury; Bone development; Bone mineral density; Skeletal health; Osteoblast; Osteoclast; Oxidative stress; Developmental toxicity; Toxicology; Environmental exposure; Systematic review; Meta-analysis

Country
United States of America

ELIGIBILITY CRITERIA

Population

Included
Human studies:

- Individuals of any age, sex, or geographic location exposed to airborne particulate matter (PM2.5, PM10) or airborne heavy metals (Pb, Cd, As, Hg).
- General population samples, community-based cohorts, birth cohorts, pediatric groups, pregnant individuals, and adult populations.
- Clinical or subclinical skeletal outcomes including bone mineral density (BMD), fractures, musculoskeletal disease, bone turnover markers, fetal skeletal growth, or developmental skeletal outcomes.

Animal studies:

- In vivo vertebrate models (e.g., rodents, zebrafish, avian species) used to examine skeletal or bone-related responses to airborne contaminants.

In vitro studies:

- Bone-relevant cell types, including osteoblasts, osteoclasts, mesenchymal stem cells, bone marrow–derived stromal or progenitor cells, and endothelial progenitor cells exposed to airborne particulate matter or heavy metals.

Excluded

- Studies involving only dietary, waterborne, or non-airborne exposure routes that are not linked to airborne contaminants.
- Studies with no bone-related or skeletal outcomes (e.g., respiratory-only or cardiovascular-only outcomes).
- Case reports or case series with fewer than 10 participants, unless they present mechanistic bone-related findings already better addressed in controlled studies.
- Non-vertebrate animal models (e.g., insects) or studies using irrelevant biological systems.
- In vitro studies using non–bone-related cell types unless they directly assess bone-relevant molecular pathways.
- Studies where exposure or outcome information is insufficient to determine an association.
- Editorials, reviews, commentaries, letters, conference abstracts without extractable original data.

Intervention(s) or exposure(s)

Included
Other Drugs Affecting Bone Structure And Mineralization; Air pollution control; Others

Eligible exposures include ambient or occupational airborne particulate matter (PM2.5, PM10, or other defined particle size fractions) and airborne heavy metals (Pb, Cd, As, Hg), whether measured as environmental concentrations, modeled exposures, personal monitoring metrics, or experimentally administered airborne/toxicant doses in animal or in vitro studies. Exposure must be delivered via inhalation, ambient air, or airborne pathways relevant to real-world environmental exposure. Studies assessing pollutant mixtures are included if they report effects of airborne PM or metals on bone outcomes.

Excluded
Studies will be excluded if the exposure is not airborne in origin, including exposures delivered exclusively via diet, water, injection, gavage, or dermal routes, and are not relevant to airborne contaminants. Studies examining chemical exposures not present in air pollution (e.g., pharmaceuticals, solvents) will be excluded unless directly linked to airborne particulate or metal exposure. Studies lacking measurable exposure characterization for PM or heavy metals, or that do not specify airborne pathways, will also be excluded.

Comparator(s) or control(s)

Included
PICO tags selected: Vehicle Control; Standard Dose; Usual Care

Eligible comparator groups include any population, animal model, or in vitro system exposed to lower, background, or zero levels of the airborne contaminants under investigation. Human epidemiological studies may compare higher

exposure strata with the lowest exposure category, reference quantiles, unexposed geographic groups, or baseline ambient air levels. Experimental animal and cell studies must include a clearly defined control condition such as vehicle control, sham exposure, air-only exposure, or standard-dose reference groups. Comparator conditions must allow an assessment of differences in bone-related outcomes attributable to variation in exposure intensity.

Excluded
Comparator groups will be excluded if they do not represent a clearly defined low, background, or no-exposure condition. Studies without any comparison group, studies where all groups receive similar levels of airborne contaminants, or studies in which exposure gradients cannot be determined will be excluded. Comparator conditions unrelated to exposure level (e.g., unrelated treatments, procedural controls, or interventions not serving as a baseline exposure group) will also be excluded. Studies lacking sufficient information to identify a valid reference category or control condition will not be included.

Study design
Only nonrandomized study types will be included.

Included
This review will include non-randomised study designs. Eligible human studies include observational designs such as cohort, case–control, and cross-sectional studies that evaluate associations between airborne contaminant exposure and bone-related outcomes. Eligible experimental studies include in vivo toxicology studies using animal models with defined airborne or metal exposures, as well as in vitro mechanistic studies using bone-relevant cell types (e.g., osteoblasts, osteoclasts, mesenchymal stem cells). Only original research with extractable data will be included.

Excluded
Randomized controlled trials and other intervention-based clinical studies will be excluded, as this review focuses on exposure-related effects rather than therapeutic interventions. Case reports, case series with fewer than 10 participants, conference abstracts without extractable data, reviews, editorials, commentaries, and opinion papers will also be excluded. Studies lacking original data, studies without a defined comparison group, and studies that do not report bone-related or skeletal outcomes will not be included. In vitro studies without bone-relevant cell types or exposure conditions will also be excluded.

Context
This review considers evidence from any global setting where exposure to airborne contaminants can occur. Eligible studies may be conducted in urban, suburban, rural, industrial, or occupational environments, as well as controlled laboratory settings for in vivo and in vitro experiments. No geographic or socioeconomic restrictions are applied. Human studies may involve community-based populations, birth cohorts, general population samples, or occupationally exposed groups. Animal and in vitro studies may be conducted in laboratory research settings. The context must involve airborne or inhalation-relevant exposure pathways and must assess bone-related or skeletal outcomes.

SIMILAR REVIEWS

Check for similar records already in PROSPERO
PROSPERO identified a number of existing PROSPERO records that were similar to this one (last check made on 1 December 2025). These are shown below along with the reasons given by that the review team for the reviews being different and/or proceeding.

- The effect of airborne particulate matter exposure on the human respiratory microbiome: a protocol for a systematic review and planned meta-analysis [published 29 April 2023] [CRD42023416230]. The review was judged **not to be similar**
- Association of Air Pollution Exposures with Osteoporosis: A Systematic Review and Meta-Analysis [published 12 August 2024] [CRD42024576104]. The review was judged **not to be similar**
- Ambient Fine Particulate Matter (PM2.5) Exposure and Incident Asthma in Children: A Systematic Review and Meta-Analysis of Cohort Studies [published 29 September 2025] [CRD420251151117]. The review was judged **not to be similar**

TIMELINE OF THE REVIEW

Date of first submission to PROSPERO
This record has not been submitted.

Review timeline
Start date: 1 January 2025. End date: 30 December 2025.

Date of registration in PROSPERO
This record has not been published.

AVAILABILITY OF FULL PROTOCOL

Availability of full protocol
A full protocol has been written but is not available because:

The protocol is embedded within a manuscript currently being prepared for journal submission. To avoid premature public release before peer review, it is not being made available. All key methodological details are fully reported in this registration.

SEARCHING AND SCREENING

Search for unpublished studies
Only published studies will be sought.

Main bibliographic databases that will be searched
The main database to be searched is *PubMed*.

Other important or specialist databases that will be searched
Google Scholar

Search language restrictions
There are no language restrictions.

Search date restrictions
Databases will be searched for articles published from 1 January 2014 and before by 24 November 2024.

Other methods of identifying studies
No other methods will be used.

Link to search strategy
A full search strategy is not available.

Selection process
Studies will be screened independently by at least two people (or person/machine combination) with a process to resolve differences.

Other relevant information about searching and screening
Searches were conducted using predefined eligibility criteria and a structured search strategy applied across PubMed and Google Scholar. All screening was performed independently by two reviewers at both the title/abstract and full-text stages, with disagreements resolved through discussion. Search limits included publication years 2014–2024, but no language restrictions. The search dates, terms, and inclusion criteria were established a priori as part of the protocol embedded within the manuscript.

DATA COLLECTION PROCESS

Data extraction from published articles and reports
Data will be extracted independently by at least two people (or person/machine combination) with a process to resolve differences.

Authors will not be contacted for further information.

Study risk of bias or quality assessment
Risk of bias will be assessed using:

Risk of bias will be assessed using an adapted OHAT framework appropriate for epidemiological, in vivo, and in vitro evidence. Domains include exposure assessment, outcome measurement, confounding, selection bias, and reporting quality. Criteria are standardized across evidence streams and based on NTP/OHAT environmental health systematic review guidance.

Data will be assessed independently by at least two people (or person/machine combination) with a process to resolve differences.

Additional information will **not** be sought from study investigators if required information is unclear or unavailable in the study publications/reports.

Reporting bias assessment
Reporting bias will be assessed qualitatively using OHAT-style criteria for selective outcome reporting, incomplete data presentation, and inconsistencies between methods and reported findings. We will consider whether planned outcomes were omitted, selectively emphasized, or insufficiently reported, and evaluate how missing or unreported results may influence overall certainty and evidence interpretation.

Certainty assessment
Certainty will be assessed using an OHAT-style evidence integration framework tailored to environmental health and toxicology. This approach evaluates the overall confidence in each body of evidence by considering study quality, risk of bias, consistency of findings, exposure–response relationships, precision, directness, and biological plausibility. Evidence from epidemiological, in vivo, and in vitro studies will be appraised both separately and in combination to determine coherence across streams. Factors such as mechanistic support, concordance in effect direction, and methodological limitations will be incorporated into confidence ratings. These domains will inform final judgments regarding the strength and certainty of the evidence.

OUTCOMES TO BE ANALYSED

Main outcomes
The main outcomes include bone development and skeletal health indicators assessed in human, in vivo, and in vitro studies. Acceptable outcomes include:

1. Bone mineral density and bone content (e.g., DXA, pQCT, micro-CT; reported as BMD/BMC, Z-scores, percent change, or standardized mean differences).
2. Bone microarchitecture (e.g., trabecular thickness, number, separation; cortical thickness; BV/TV; assessed via μCT or histomorphometry).
3. Osteoblast and osteoclast activity markers (e.g., ALP, RUNX2, OCN, OPN, RANKL/OPG, TRAP activity; measured via biochemical assays, gene/protein expression, or imaging).
4. Bone formation, resorption, and mineralization outcomes (e.g., mineral apposition rate, calcium deposition, collagen formation; assessed by histology, Alizarin Red, von Kossa, qPCR, or ELISA).
5. Bone marrow adiposity (e.g., adipocyte number/size, lipid accumulation; Oil Red O staining, histology).
6. Epigenetic or mechanistic bone outcomes (e.g., DNA methylation, oxidative stress markers, inflammatory mediators relevant to bone pathways).

Effect measures will include relative risks, odds ratios, hazard ratios, regression coefficients, mean differences, standardized mean differences, or percent changes, depending on study design and available data.

Additional outcomes

Additional outcomes include biomarkers and physiological indicators related to bone-relevant systemic or mechanistic pathways. These include oxidative stress markers (e.g., ROS generation, antioxidant enzyme activity such as SOD, CAT, GPx), inflammatory mediators linked to bone turnover (e.g., TNF-α, IL-6, IL-1β), and endocrine or metabolic markers influencing skeletal biology (e.g., PTH, vitamin D, IGF-1). Measurements may be obtained via ELISA, qPCR, Western blotting, immunostaining, or biochemical assays. Additional developmental or functional outcomes such as body weight, growth indices, or delayed ossification will be included when relevant. Effect measures may include mean differences, standardized mean differences, percent changes, or regression coefficients.

PLANNED DATA SYNTHESIS

Strategy for data synthesis

Data will be combined using a structured narrative synthesis and evidence-integration framework appropriate for environmental health, toxicology, and multi-stream systematic reviews. Because the included studies span epidemiological, in vivo, and in vitro evidence streams and use heterogeneous exposure metrics, outcome measures, and analytical approaches, quantitative meta-analysis is not planned. Instead, a structured synthesis following OHAT-style principles will be used to evaluate consistency, coherence, and biological plausibility across evidence streams.

Studies will first be grouped by evidence stream (human, in vivo animal, and in vitro mechanistic), by exposure type (e.g., PM2.5, PM10, heavy metals), and by bone-related outcome domain (bone mineral density/content, microarchitecture, osteoblast/osteoclast activity, mineralization, adiposity, and mechanistic biomarkers). Within each group, key information will be extracted and summarized, including the direction and magnitude of the association or difference, exposure–response gradients, statistical significance, and relevant methodological characteristics.

The synthesis will examine patterns of consistency within each stream by comparing whether results point in a similar direction, whether exposure–response relationships are present, and whether findings remain robust across study designs, populations, or experimental models. Risk of bias assessments will inform the interpretation of patterns: studies at higher risk of bias will be considered with caution, and sensitivity will be given to higher-quality evidence. Divergent findings will be examined in relation to exposure characterization, differences in outcome measurement, species differences, dose levels, and methodological limitations.

Next, cross-stream integration will be conducted to evaluate coherence between human findings and experimental evidence. This analysis will consider whether associations observed in epidemiological studies correspond with mechanistic pathways demonstrated in vitro or with phenotypic skeletal outcomes in animal studies. Convergence of human, in vivo, and in vitro evidence will be interpreted as stronger overall support for an effect. Biological plausibility will be evaluated by examining whether mechanistic findings (e.g., oxidative stress, inflammation, impaired osteogenesis) logically support observed structural or functional bone outcomes.

Subgroups defined by exposure type (PM vs. metals), life stage (early-life vs. adult), or bone outcome category will be synthesized separately when data allow. The direction, coherence, and strength of the evidence will be summarized for each outcome domain, and overall conclusions will be drawn by integrating findings across streams. Final interpretations will include ratings of the strength and certainty of the evidence, informed by consistency, biological plausibility, methodological quality, and the robustness of the reported outcomes.

CURRENT REVIEW STAGE

Stage of the review at this submission

Review stage	Started	Completed
Pilot work	✓	✓
Formal searching/study identification	✓	✓

Review stage	Started	Completed
Screening search results against inclusion criteria	✓	✓
Data extraction or receipt of IPD		
Risk of bias/quality assessment		
Data synthesis		

Review status
The review is currently planned or ongoing.

Publication of review results
Results of the review will be published in English.

REVIEW AFFILIATION, FUNDING AND PEER REVIEW

Review team members
Dr Nicole Sparks (review guarantor and contact) ORCID: 0000-0001-6176-7895. University of California Irvine. United States of America.

No conflict of interest declared.

Madeline Vera-Colon. University of California Irvine. United States of America.

No conflict of interest declared.

Ruth Meletz. University of California Irvine. United States of America.

No conflict of interest declared.

Named contact
Dr Nicole Sparks (nrsparks@hs.uci.edu). ORCID: 0000-0001-6176-7895. University of California Irvine. United States of America.

Review affiliation
University of California Irvine, Department of Environmental and Occupational Health, Irvine, USA

Funding source
National Institutes of Health (NIH).

Grant number
R00ES032486

Peer review
There has been no peer review of this planned review.

ADDITIONAL INFORMATION

Additional information
This review integrates epidemiological, in vivo, and in vitro evidence to evaluate how airborne particulate matter and airborne heavy metals influence bone development and skeletal health. Because study designs, exposure metrics, and outcome measures vary widely across evidence streams, a structured narrative synthesis and OHAT-style evidence integration approach will be used rather than meta-analysis. The review aims to provide a comprehensive, mechanistic, and multi-level assessment of how contemporary air pollution mixtures impact bone-related outcomes across the lifespan.

Review conflict of interest
Declared individual interests are recorded under team member details.. No additional interests are recorded for this review.

Medical Subject Headings

Air Pollution; Particulate Matter; Metals, Heavy; Bone Development; Bone Density; Osteoblasts; Osteoclasts; Bone and Bones; Bone Marrow; Oxidative Stress; Inflammation; Epidemiologic Studies; Animals; Cell Line; In Vitro Techniques

PROSPERO version history

No preview available

Disclaimer

The content of this record displays the information provided by the review team. PROSPERO does not peer review registration records or endorse their content.

PROSPERO accepts and posts the information provided in good faith; responsibility for record content rests with the review team. The guarantor for this record has affirmed that the information provided is truthful and that they understand that deliberate provision of inaccurate information may be construed as scientific misconduct.

PROSPERO does not accept any liability for the content provided in this record or for its use. Readers use the information provided in this record at their own risk.

Any enquiries about the record should be referred to the named review contact

PRISMA 2020 Checklist

Section and Topic	Item #	Checklist item	Location where item is reported
**TITLE**			
Title	1	Identify the report as a systematic review.	Manuscript Title; keywords
**ABSTRACT (2) nested in full checklist**			
Objectives	2	Provide an explicit statement of the main objective(s) or question(s) the review addresses.	Yes
Eligibility Criteria	3	Specify the inclusion and exclusion criteria for the review.	Yes
Information Sources	4	Specify the information sources (e.g. databases, registers) used to identify studies and the date when each was last searched.	Yes
Risk of Bias	5	Specify the methods used to assess risk of bias in the included studies.	Yes
Synthesis of Results	6	Specify the methods used to present and synthesis results.	Yes
Included Studies	7	Give the total number of included studies and participants and summarise relevant characteristics of studies.	Yes
Synthesis of Results	8	Present results for main outcomes, preferably indicating the number of included studies and participants for each. If meta-analysis was done, report the summary estimate and confidence/credible interval. If comparing groups, indicate the direction of the effect (i.e. which group is favored).	Yes
Limitations of Evidence	10	Provide a brief summary of the limitations of the evidence included in the review (e.g. study risk of bias, inconsistency and imprecision).	Not explicit; yes
Interpretation	11	Provide a general interpretation of the results and important implications.	Yes
Funding	12	Specify the primary source of funding for the review.	Yes
Registration	13	Provide the register name and registration number.<br>From: PROSPERO submitted, not confirmed yet	Yes
**INTRODUCTION**			
Rationale	3	Describe the rationale for the review in the context of existing knowledge.	Introduction: throughout
Objectives	4	Provide an explicit statement of the objective(s) or question(s) the review addresses.	Introduction: final sentence
**METHODS**			
Eligibility criteria	5	Specify the inclusion and exclusion criteria for the review and how studies were grouped for the syntheses.	Methods: *2.3 Inclusion and Exclusion Criteria and Study Selection* (**Table 2; Figure 1**)
Information sources	6	Specify all databases, registers, websites, organisations, reference lists and other sources searched or consulted to identify studies. Specify the date when each source was last searched or consulted.	Methods: *2.2 Search Strategy and Information Sources* (**Table 1**)
Search strategy	7	Present the full search strategies for all databases, registers and websites, including any filters and limits used.	Methods: *2.2 Search Strategy and Information Sources; 2.3 Inclusion and Exclusion Criteria and Study Selection* (**Table 1; Table 2; Figure 1**)

Selection process	8	Specify the methods used to decide whether a study met the inclusion criteria of the review, including how many reviewers screened each record and each report retrieved, whether they worked independently, and if applicable, details of automation tools used in the process.	Methods: *2.3 Inclusion and Exclusion Criteria and Study Selection* (**Table 2; Figure 1**)
**Section and Topic**	**Item #**	**Checklist item**	**Location where item is reported**
Data collection process	9	Specify the methods used to collect data from reports, including how many reviewers collected data from each report, whether they worked independently, any processes for obtaining or confirming data from study investigators, and if applicable, details of automation tools used in the process.	Methods: *2.3 Inclusion and Exclusion Criteria; 2.4 Data Extraction* (**Table 2; Figure 1; Table S1; Additional Materials-Extraction**)
Data items	10a	List and define all outcomes for which data were sought. Specify whether all results that were compatible with each outcome domain in each study were sought (e.g. for all measures, time points, analyses), and if not, the methods used to decide which results to collect.	Methods: *2.4 Data Extraction* (**Table S1; Additional Materials-Extraction**)
	10b	List and define all other variables for which data were sought (e.g. participant and intervention characteristics, funding sources). Describe any assumptions made about any missing or unclear information.	Methods: *2.4 Data Extraction* (**Table S1; Additional Materials-Extraction**)
Study risk of bias assessment	11	Specify the methods used to assess risk of bias in the included studies, including details of the tool(s) used, how many reviewers assessed each study and whether they worked independently, and if applicable, details of automation tools used in the process.	Methods: *2.5 GRADE and Risk of Bias Assessment* (**Table S2**)
Effect measures	12	Specify for each outcome the effect measure(s) (e.g. risk ratio, mean difference) used in the synthesis or presentation of results.	Methods: *2.6 Relative Risk Ratio Meta-analysis* (**Figure 2; Additional Materials-2x2 Tables**)
Synthesis methods	13a	Describe the processes used to decide which studies were eligible for each synthesis (e.g. tabulating the study intervention characteristics and comparing against the planned groups for each synthesis (item #5)).	Methods: *2.3 Inclusion and Exclusion Criteria; 2.4 Data Extraction* (**Table 2; Figure 1; Additional Materials-Extraction; Table S1**)
	13b	Describe any methods required to prepare the data for presentation or synthesis, such as handling of missing summary statistics, or data conversions.	Methods: *2.6 Relative Risk Ratio Meta-analysis* (**Figure 2; Additional Materials-2x2 Tables**)
	13c	Describe any methods used to tabulate or visually display results of individual studies and syntheses.	Methods/Results: *2.6 Relative Risk Ratio Meta-analysis*; *3.3 GRADE Summary; 3.7 Relative Risk Ratio Meta-analysis* (**Figure 2; Table 3; Table S1; Table S2**)
	13d	Describe any methods used to synthesize results and provide a rationale for the choice(s). If meta-analysis was performed, describe the model(s), method(s) to identify the presence and extent of statistical heterogeneity, and software package(s) used.	Methods: *2.6 Relative Risk Ratio Meta-analysis* (DerSimonian–Laird, Q, I^2, τ^2) (**Figure 2; Additional Materials- 2x2 Tables**)
	13e	Describe any methods used to explore possible causes of heterogeneity among study results (e.g. subgroup analysis, meta-regression).	Methods/Results: *2.6 Relative Risk Ratio Meta-analysis; 3.7 Relative Risk Ratio Meta-analysis* DerSimonian–Laird, Q, I^2, τ^2) (**Figure 2**)

	13f	Describe any sensitivity analyses conducted to assess robustness of the synthesized results.	Not performed / No sensitivity analyses reported
Reporting bias	14	Describe any methods used to assess risk of bias due to missing results in a synthesis (arising from reporting biases).	Methods/Discussion: *2.5 GRADE and Risk of Bias Assessment; 4.5*
**Section and Topic**	**Item #**	**Checklist item**	**Location where item is reported**
assessment			*Limitations of Current Literature*
Certainty assessment	15	Describe any methods used to assess certainty (or confidence) in the body of evidence for an outcome.	Methods/Results: *2.5 GRADE and Risk of Bias Assessment; 3.3 GRADE Summary* (**Table 3**)
**RESULTS**			
Study selection	16a	Describe the results of the search and selection process, from the number of records identified in the search to the number of studies included in the review, ideally using a flow diagram.	Results: *3.1 Study Selection* (**Figure 1**)
	16b	Cite studies that might appear to meet the inclusion criteria, but which were excluded, and explain why they were excluded.	Results: *3.1 Study Selection* (**Figure 1**)
Study characteristics	17	Cite each included study and present its characteristics.	Results: *3.2 Study Characteristics* (**Table S1; Additional Materials-Extractions**)
Risk of bias in studies	18	Present assessments of risk of bias for each included study.	Results: *3.6 Risk of Bias* (**Table S2**)
Results of individual studies	19	For all outcomes, present, for each study: (a) summary statistics for each group (where appropriate) and (b) an effect estimate and its precision (e.g. confidence/credible interval), ideally using structured tables or plots.	Results: *3.4 Exposure to air pollution and/or PM; 3.5 Exposure to heavy metals; 3.7 Relative Risk Ratio Meta-Analysis* (**Table S1; Additional Materials- Extractions; Additional Materials- 2x2 Tables**)
Results of syntheses	20a	For each synthesis, briefly summarise the characteristics and risk of bias among contributing studies.	Results: *3.3 GRADE Summary; 3.6 Risk of Bias; 3.7 Relative Risk Ratio Meta-analysis* (**Table 3; Table S2**)
	20b	Present results of all statistical syntheses conducted. If meta-analysis was done, present for each the summary estimate and its precision (e.g. confidence/credible interval) and measures of statistical heterogeneity. If comparing groups, describe the direction of the effect.	Results: *3.7 Relative Risk Ratio Meta-analysis* (**Figure 2; Additional Materials- 2x2 Tables**)
	20c	Present results of all investigations of possible causes of heterogeneity among study results.	Results: *3.7 Relative Risk Ratio Meta-analysis* (**Figure 2**)
	20d	Present results of all sensitivity analyses conducted to assess the robustness of the synthesized results.	Not performed / No sensitivity or subgroup analyses reported
Reporting biases	21	Present assessments of risk of bias due to missing results (arising from reporting biases) for each synthesis assessed.	Results/Discussion: *3.6 Risk of Bias; 4.5 Limitations of current evidence* (**Table S2**)
Certainty of evidence	22	Present assessments of certainty (or confidence) in the body of evidence for each outcome assessed.	Results/Discussion: *3.3 GRADE Summary; 4.0 Discussion* (**Table 3**)
**DISCUSSION**			
Discussion	23a	Provide a general interpretation of the results in the context of other evidence.	*Discussion* (**Figure 2; Figure 3; Table 3; Table S1; Table S2**)

23b	Discuss any limitations of the evidence included in the review.	Discussion: *4.5 Limitations of current evidence*

Section and Topic	Item #	Checklist item	Location where item is reported
	23c	Discuss any limitations of the review processes used.	Discussion: *4.5 Limitations of current evidence*
	23d	Discuss implications of the results for practice, policy, and future research.	Discussion/Conclusions: *4.1 Potential mechanisms to be explored further; 4.2 Developmental vulnerability; 4.3 Heavy metals alter bone homeostasis through oxidative and cellular pathways; 4.4 Integration of skeletal endpoints into environmental health assessments; 5.0 Conclusions* (**Figure 3; Table 3**)
**OTHER INFORMATION**			
Registration and protocol	24a	Provide registration information for the review, including register name and registration number, or state that the review was not registered.	Abstract/Methods: *2.1 Protocol* (**Additional Materials- Protocol**)
	24b	Indicate where the review protocol can be accessed, or state that a protocol was not prepared.	Methods: *2.1 Protocol* (**Additional Materials- Protocol**)
	24c	Describe and explain any amendments to information provided at registration or in the protocol.	Methods/Discussion: *2.2 Search Strategy and Information Sources; 4.5 Limitations in current evidence* Amendment after protocol was established: For Google Scholar search strategy, only first 100 web pages were screened due to an excessive high volume of studies included.
Support	25	Describe sources of financial or non-financial support for the review, and the role of the funders or sponsors in the review.	*Abstract; Acknowledgments*
Competing interests	26	Declare any competing interests of review authors.	*Declaration of competing interests*
Availability of data, code and other materials	27	Report which of the following are publicly available and where they can be found: template data collection forms; data extracted from included studies; data used for all analyses; analytic code; any other materials used in the review.	Methods: *2.6 Relative Risk Ratio Meta-analysis* include GitHub: https://github.com/mveracolon/Bone-Development-Air-Pollution.git; *Data availability* (**Additional Materials-Protocol; Additional Materials-Extraction; Additional Materials-2x2 Tables**)

From: Page MJ, McKenzie JE, Bossuyt PM, Boutron I, Hoffmann TC, Mulrow CD, et al. The PRISMA 2020 statement: an updated guideline for reporting systematic reviews. BMJ 2021;372:n71. doi: 10.1136/bmj.n71. This work is licensed under CC BY 4.0. To view a copy of this license, visit https://creativecommons.org/licenses/by/4.0/

Extraction Files

Epidemiological Studies:

Covidence #49
Study ID: Farhi 2014
Total number of participants: 216730

	Spontaneous	ART (p-value)
Total	207,825	8905; (<0.0001)
Maternal age ((17-19); (20-24); (25-29); (30-34); (35-39); (40-44))	(4938); (45,996); (70,001); (52,250); (27,238); (7,367)	(1); (448); (2,739); (3,371); (1,700); (646) (<0.0001)
Maternal Ethnicity ((Jewish); (others))	(125,770); (82,052)	(8002); (903) (<0.0001)
Maternal country of birth ((Israel); (other))	(177,188); (30,637)	(7,021); (1,884) (<0.0001)
Maternal education ((<12); (13+); (unknown))	(77,168); (51,044); (79,613)	(3,325); (3,518); (2,062) (<0.0001)
plurality ((singleton); (multiple))	(198,334); (9,491)	(4,294); (4,611) (<0.0001)
season of birth ((winter); (spring); (summer); (fall))	(49,783); (52,215); (55,156); (50,671)	(2,099); (2,165); (2,259); (2,382) (<0.0001)
infant gender ((M); (F))	(106,670); (101,155)	(4,486); (4,419) (0.08)

exposure conc.	Mean +/- SD
SO2 (1, 2, entire)	(2.81 +/- 0.96); (2.74 +/- 0.92); (2.74 +/- 0.75)
PM10 (1, 2, entire)	(53.5 +/- 13.6); (52.7 +/- 13); (52 +/- 8.3)
NOx (1, 2, entire)	(27.8 +/- 22.6); (26.8 +/- 20.4); (26.3 +/- 16.6)
O3 (1, 2, entire)	(32.4 +/- 6.2); (32.7 +/- 6.3); (32.1 +/- 4.5)

Covidence #44
Study ID: Cheng 2024
Total number of participants: 104736

	Incident of musculoskeletal diseases (n = 104,736)	Non-incident of musculoskeletal diseases (n = 166,585)
age	59.23+/- 7.23	55.39 +/- 7.98
median follow-up time	10.85	10.77
person-years	1,135,752	1,796,280
sex (F:M%)	57.07 / 42.94	52.45 / 47.55
BMI	28.03 +/- 4.58	26.68 +/- 4.15
education score (1, 2, 3, 4, 5 %)	21.34 / 17.59 / 4.72 / 29.94 / 26.41	11.82 / 17.13 / 5.76 / 28.21 / 37.08
annual household income (>100, 52-100, 31-51, 18-30, <18, idk, prefer not %)	2.91 / 14.1 / 21.97 / 24.6 / 21.67 / 3.95 / 10.8	6.45 / 22.65 / 25.65 / 20.86 / 13.76 / 2.65 / 8.26
smoking (never, ever %)	59.47 / 40.53	67.82 / 32.18
alcohol intake (never, ever %)	5.21 / 94.79	3.68 / 96.32
townsend deprivation index	-1.67 +/- 2.84	-1.81 +/- 2.73
physical activity (min/d)	54.14 +/- 70.14	47.62 +/- 61.65

duration of residence	18.95 +/- 12.87	16.82 +/- 11.94
air pollution (2.5, 10, NO2, NOx, APscore) (ug/m3)	9.93 / 6.39 / 28.13 / 16.95 / 47.34	9.89 / 6.4 / 28.22 / 16.66 / 46.9

Model 1 (HR (95% CI) / p value)	Degenerative JD	Dorsopathies	IR	Osteoporosis	Total MD
PM2.5	1.054 (1.036-1.072) / 2.52 * 10^-9	1.11 (1.085-1.137) / 4.01 * 10^-18	1.061 (1.036-1.086) / 1.18 * 10^-6	1.079 (1.035-1.125) / 3.79 * 10^-4	1.032 (1.021-1.043) / 8.24 * 10^-9
PM10	1.047 (1.032-1.063) / 8.56 * 10^-10	1.062 (1.04-1.084) / 1.39 * 10^-8	1.069 (1.047-1.091) / 2.64 * 10^-10	1.034 (0.997-1.072) / 7.03 * 10^-2	1.03 (1.021-1.04) / 1.13 * 10^-10
NO2	1.005 (1.003-1.007) / 3.34 * 10^-5	1.007 (1.003-1.01) / 8.41 * 10^-5	1.018 (1.015-1.021) / 2.76 * 10^-37	1.013 (1.007-1.019) / 9.78 * 10^-6	1.007 (1.006-1.009) / 7.52 * 10^-22
NOx	1.003 (1.001-1.005) / 4.31 * 10^-4	1.002 (0.999-1.004) / 1.5 * 10^-1	1.003 (1-1.005) / 1.89 * 10^-2	0.999 (0.994-1.003) / 4.61 * 10^-1	1.001 (1-1.002) / 7.12 * 10^-3

Model 2 (HR (95% CI) / p value)	Degenerative JD	Dorsopathies	IR	Osteoporosis	Total MD
PM2.5	1.014 (0.997-1.032) / 1.08 *10^-1	1.065 (1.039-1.091) / 3.92 * 10^-7	1.007 (0.983-1.032) / 5.8 * 10^-1	1.064 (1.02-1.11) / 4.14 * 10^-3	1.004 (0.993-1.014) / 5.17 * 10^-1
PM10	1.035 (1.02-1.051) / 4.21 * 10^-6	1.05 (1.028-1.072) / 4.88 * 10^-6	1.059 (1.037-1.081) / 6.97 * 10^-8	1.032 (0.995-1.07) / 9.17 * 10^-2	1.021 (1.012-1.031) / 4.95 * 10^-6
NO2	1.005 (1.003-1.008) / 3.46 * 10^-5	1.006 (1.002-1.009) / 9.08 * 10^-4	1.017 (1.013-1.02) / 4 * 10^-22	1.007 (1.001-1.012) / 2.58 * 10^-2	1.007 (1.006-1.009) / 1.48 * 10^-20
NOx	1.002 (1-1.003) / 4.51 * 10^-2	1 (0.998-1.002) / 9.63 * 10^-1	1.001 (0.999-1.004) / 2.19 * 10^-1	0.999 (0.995-1.003) / 5.43 * 10^-1	1 (0.999-1.001) / 4.21 * 10^-1

Covidence #36
Study ID: Qi 2023
Total number of participants: 446395

	no incident fractures	yes incident fractures
gender, female	232,569	8,019
age	56.44	58.95
Ethnicity, White European	407,202	11,914
Household income (<18, 18-30, 31-51, 52-100, >100) (%)	18.9 / 21.6 / 22.4 / 17.4 / 4.6	25.5 / 22 / 17.9 / 12.7 / 3.4
Season (Spring, Summer, Fall, Winter) (%)	29.2 / 26.2 / 23.7 / 20.8	28.6 / 26.1 / 24.8 / 20.5
BMI (kg/m2)	27.42	27.28
Serum 25(OH)D (nmol/L)	48.33	48.02
PM2.5 (ug/m3)	9.98	10.03
PM2.5-10	6.43	6.43
PM10	19.22	19.17
NOx	44.01	44.7

NO2	29.03	29.2
AP score	108.26	108.99

HR (95% CI) / p value	Model 1	Model 2	Model 3
PM2.5	1.91 (1.61, 2.28) / <0.01	1.79 (1.5, 2.13) / <0.01	1.7 (1.42, 2.03) / <0.01
PM2.5-10	1.1 (0.9, 1.35) / 0.04	1.08 (0.88, 1.33) / 0.08	1.07 (0.87, 1.32) / 0.1
PM10	1.16 (1.05, 1.28) / <0.01	1.12 (1.01, 1.23) / 0.03	1.1 (1, 1.21) / 0.07
NO2	1.09 (1.07, 1.12) / <0.01	1.08 (1.06, 1.11) / <0.01	1.08 (1.06,1.1) / <0.01
NOx	1.05 (1.04, 1.06) / <0.01	1.04 (1.03, 1.06) / <0.01	1.04 (1.03, 1.05) / <0.01
AP score	1.03 (1.02, 1.04) / <0.01	1.03 (1.02, 1.03) / <0.01	1.03 (1.02, 1.03) / <0.01

Covidence #32
Study ID: Liu 2015
Total number of participants: 2264

	osteocalcin (u, sd, p)	CTx (u, sd, p)
cohort (GINIplus / LISAplus)	(91.1, 30.5, <0.001) / (99.1, 33.5, <0.001)	(580.3, 290.9, <0.001) / (878.2, 346.5, <0.001)
city (munich / wesel)	(95.8, 32, <0.001) / (98.8, 34.7, <0.001)	(726.1, 330.2, <0.001) / (543.8, 306.8, <0.001)
sex (male / female)	(87.9, 27.1, <0.001) / (98.8, 34.7, <0.001)	(643.2, 315, 0.022) / (675.3, 351.1, 0.022)
puberty (N / Y)	(89.3, 27.9, <0.001) / (102.3, 37.2, <0.001)	(638.6, 313.1, <0.001) / (704.3, 372.9, <0.001)
BMI @ 10-years (norm / ow / obese)	(93.1, 31.1, 0.049) / (95.8, 33.7, 0.049) / (86.7, 28, 0.049)	(658.4, 331.6, 0.916) / (662.4, 344.8, 0.916) / (672.4, 322.5, 0.916)
fasting (Y/N)	(106.3, 34.3, <0.001) / (90.4, 30.2, <0.001)	(928.8, 377.7, <0.001) / (600.6, 292.1, <0.001)
parental education (L / M / H)	(90.5, 32.5, 0.219) / (91.8, 30.3, 0.219) / (93.9, 31.7, 0.219)	(625.9, 348.5, <0.001) / (604.4, 331.5, <0.001) / (682.5, 329.7, <0.001)
season ((mar-may) / (june-aug) / (sep-nov) / (dec-feb))	(95.2, 32.8, 0.007) / (90.8, 28.4, 0.007) / (91.9, 33.2, 0.007) / (96.3, 31.7, 0.007)	(676.3, 350.9, <0.001) / (588.1, 262.7, <0.001) / (647.8, 352.2, <0.001) / (752.2, 353, <0.001)
hours of physical activity/week (low / medium / high)	(93.6, 31.9, 0.915) / (93.2, 31.3, 0.915) / (92.8, 31.8, 0.915)	(675.3, 334.5, 0.033) / (666.6, 332.2, 0.033) / (627.5, 330.5, 0.033)
age in months (low, medium, high)	(93.6, 27.6, 0.014) / (91.7, 30.7, 0.014) / (96.7, 37.1, 0.014)	(665.4, 310.4, 0.038) / (644.9, 335.5, 0.038) / (689.9, 352, 0.038)

Association w osteocalcin est change (iq range)*	Model 1	Model 2	Model 3
NO2	2.4 (0.5, 4.3)*	2.3 (0.5, 4.2)*	2.5 (0.6, 4.4)*
PM2.5	-3 (-9.4, 3.3)	-3.6 (-9.8, 2.6)	-3.9 (-10.2, 2.3)
PM2.5-10	2.8 (-0.1,5.7)*	2.8 (0, 5.7)*	3 (0.1, 5.8)*
PM10	3.1 (0,6.3)	3.2 (0.1, 6.3)*	3.2 (0, 6.4)*
PM2.5 absorbance	1.4 (-1.5, 4.4)	1.2 (-1.7, 4.1)	1.3 (-1.8, 4.3)
distance to major road	-0.4 (-1.2, 0.3)	-0.4 (-1.4, 0.3)	-0.4 (-1.1, 0.3)

Association w CTx est change (iq	Model 1	Model 2	Model 3

range)*			
NO2	29.5 (10, 49.1)*	25.2 (6.8, 43.6)*	24 (6.7, 41.3)*
PM2.5	-41.8 (-107.6, 24)	-50.4 (-112.2, 11.5)	-53.7 (-109.9, 2.7)
PM2.5-10	42.6 (12.5, 72.7)*	36.9 (8.6, 65.2)*	32.3 (6.1, 58.5)*
PM10	34.9 (1.7, 68)*	35 (3.8, 66.1)*	30.7 (1.7, 59.7)*
PM2.5 absorbance	26.5 (-4.2, 57.3)	20.8 (-8.1, 49.7)	15.3 (-12.5, 43.1)
Distance to major road	-6.2 (-13.6, 1.3)	-5.8 (-12.9, 1.2)	-3.6 (-10.2, 2.9)

Covidence #10
Study ID: Maghbooli 2018
Total number of participants: 100

	polluted (48)	non-polluted (44); p-value
sex (F)	24	18; 0.35
gestational age	38.93	38.48; 0.09
preterm (<37 wks)	0	1; 0.44
season at birth (winter, fall)	(21); (27)	(12); (32); 0.09
apgar score 1min (9, 8, <8)	(42); (3); (3)	(41); (3); (0); 0.62
apgar score 5min (9-10, <9)	(46); (2)	(44); (0); 0.63
birth weight	3243..25	3285.25; 0.64
birth length	50.06	50.3; 0.67
head circumference	34.36	34.73; 0.27
chest circumference	33.93	33.3; 0.4

PM2.5	polluted	non-polluted	p-value
whole pregnancy	37.12	25.18	0.0001
1st	30.99	20.43	0.0001
2nd	38.44	26.37	0.0001
3rd	42.44	29.04	0.0001

PM10	polluted	non-polluted	p-value
Whole pregnancy	91.45	70.43	0.0001
1st	74.34	64.97	0.01
2nd	94.88	74.06	0.0001
3rd	104.89	72.13	0.0001

Covidence #06
Study ID: Cao 2019
Total number of participants: 7965

	N
maternal age ((22-28) / (29-35) / (>35))	2783 / 4516 / 666

registered residence (permanent / migrant)	4755 / 3209
party (1 / 2)	6405 / 1559
gravidity (1 / >2)	5076 / 2883
SMI (N / Y)	2448 / 5489
GH (N / Y)	7567 / 398
GDM (N / Y)	6771 / 1194
BMI (UW / NW / OW / obese)	573 / 6129 / 1062 / 201
height (mean/cm)	161.67 +/- 4.44
weight (mean/kg)	57.11 +/- 11.99
gender (M / F)	4045 / 3920
season of conception (sp / su / aut / win)	2216 / 1839 / 1756 / 2154
PTB (N / Y)	7586 / 379
LBW (N / Y)	7732 / 233

Association bt PM2.5 & ultrasound fetal growth beta (95%CI)	crude	adjusted-1	adjusted-2	adjusted-3 (full model)
AC	-0.62 (-3.35, 2.11)	-5.71 (-9.17, -2.25)	-5.57 (-9.06, -2.08)	-5.48 (-9.06, -1.91)
BPD	-1.63 (-2.48, -0.78)	-5.82 (-6.88, -4.75)	-5.77 (-6.84, -4.69)	-5.57 (-6.66, -4.47)
FL	-2.21 (-2.91, -1.5)	-5.82 (-6.71, -4.92)	-5.73 (-6.663, -4.83)	-5.47 (-6.39, -4.55)
EFW-H	-18.11 (-19.33, -16.96)	-14.45 (-16.96, -15.72)	-14.53 (-15.81, -13.34)	-14.49 (-16.05, -13.49)
EFW-S	-16.63 (-17.68, -15.63)	-13.4 (-14.5, -12.38)	-13.44 (-14.56, -12.41)	-13.56 (-14.71, -12.5)

Experimental Studies:

Covidence #13
Study ID: Li 2021

Genes (q <=0.05)	Col12a1	Flnc	Fbln2	Cd5l	Ccl5	Lgr6	Krt19	Itga3	Tead4	Serpinb9b	Fcgr1	Cobll1	Helz2	Tnfrsf21	Atp6v1c2	Oas2	Lyz2	Zfp36
miRNA (q <= 0.05)	miR-3106-5p	miR-1899	miR-222-5p	miR-34b-5p	miR-450a-3p	miR-314-3p	miR-6239	miR-378a-3p	miR-27b-3p	miR-214-3p	miR-708-5p	miR-34c-5p	miR-450a-5p	miR-365-3p	miR-484	miR-30d-3p	miR-7043-3p	miR-500-3p

Covidence #08
Study ID: Ge 2023

femur measurements (*)	FA	PM
length	15	14.8*
width	2.4	2.3
cortical BMD	1.05	1.1
trabecular BMD	0.06	0.05

Staining quant (*)	FA	PM
OCN+ ratio	15	10*
OCN levels in serum	150	100***

ALP+ratio	10	10
BALP levels in serum	8	24*
ALP expression	1	1.5*

Covidence #04
Study ID: Abu-Elmagd 2017

PM2.5 (MTT)	24h	48h	72h
ctl	0.17	0.15	0.17
15ug; 25ug	.21; .2	.28; .28	.27; .26
50ug;	0.17	0.3	0.26
150ug	0.15	0.14	0.16
300ug	0.14	0.14	0.15

PM10 (MTT)	24h	48h	72h
ctl	0.17	0.19	0.23
15ug; 25ug	.16; .17	.17; .16	.19; .23
50ug	0.15	0.14	0.17
150ug	0.16	0.16	0.15
300	0.15	0.14	0.13

(gene expression) (RQ*)	control	PM2.5 (150ug/mL)	PM10 (150ug/mL)
BCL2	1	2	2
BAX	1	1	0.5
p53	1	0.5	0.5
TNF-a	1	3	8*
IL6	1	5*	8*

Covidence #02
Study ID: Bhattarai 2023

fold change (GE)	Control	PM2.5
RANKL	1	2*
OPG	1	0.5*

OD	control	PM2.5
IL-1a	0.1	0.25*
IL-2	0.1	0.16*
IL-6	0.1	0.16*
IFN-Y	0.05	0.15*
TNF-a	0.1	0.2*

2x2 Tables

Bone loss from heavy metal exposure

		Outcome	No Outcome
**Liu et al., 2016**	Exposure	3	0
	No Exposure	0	3
**Xu et al., 2014**	Exposure	3	0
	No Exposure	0	3
**Wu et al., 2014**	Exposure	3	0
	No Exposure	0	3
**Zhao et al., 2014**	Exposure	3	0
	No Exposure	0	3
**Wang et al., 2022**	Exposure	12	0
	No Exposure	0	12
**Tomaszewska et al., 2016**	Exposure	12	0
	No Exposure	0	12
**Rafiei et al., 2018**	Exposure	8	0
	No Exposure	0	8
**Zhao et al., 2015**	Exposure	9	0
	No Exposure	0	9
**Zhang et al., 2020**	Exposure	7	0
	No Exposure	0	5
**Chen et al., 2022**	Exposure	10	0
	No Exposure	0	10
**Song et al., 2023**	Exposure	6	0
	No Exposure	0	6
**Tong et al., 2023**	Exposure	3	0
	No Exposure	0	3
**Wan et al., 2023**	Exposure	21.11	45.89
	No Exposure	14.35	52.65
**Torres-Rodriguez et al., 2022**	Exposure	10	0
	No Exposure	0	10
**Luo et al., 2021**	Exposure	3	0
	No Exposure	0	3
**Ma et al., 2021a**	Exposure	3	0
	No Exposure	0	3
**Ma et al., 2021b**	Exposure	3	0
	No Exposure	0	3
**Duranova et al., 2014**	Exposure	10	0
	No Exposure	0	10

**Brozoska et al., 2016**	Exposure	7	0
	No Exposure	0	8
**He et al., 2019**	Exposure	3	0
	No Exposure	0	3
**Liu et al., 2019**	Exposure	3	0
	No Exposure	0	3
**Buha et al., 2019**	Exposure	7	0
	No Exposure	0	7
**Liu et al., 2020**	Exposure	3	0
	No Exposure	0	3
**Cumulative**	Exposure	155.11	45.89
	No Exposure	14.35	188.65

Impaired Osteoblast Differentiation

		**Outcome**	**No Outcome**
**Xu et al., 2014**	Exposure	3	0
	No Exposure	0	3
**Wu et al., 2014**	Exposure	3	0
	No Exposure	0	3
**Zhao et al., 2014**	Exposure	3	0
	No Exposure	0	3
**Al-Ghafari et al., 2019**	Exposure	9	0
	No Exposure	0	9
**Beier et al., 2015**	Exposure	3	0
	No Exposure	0	3
**Rafiei et al., 2018**	Exposure	8	0
	No Exposure	0	8
**Wang et al., 2022a**	Exposure	12	0
	No Exposure	0	12
**Zhao et al., 2023**	Exposure	3	0
	No Exposure	0	3
**Wu et al., 2019**	Exposure	3	0
	No Exposure	0	3
**Lv et al., 2019**	Exposure	8	0
	No Exposure	0	8
**Papa et al., 2015**	Exposure	3	0
	No Exposure	0	3
**Ma et al., 2021a**	Exposure	3	0
	No Exposure	0	3

**Ma et al., 2021b**	Exposure	3	0
	No Exposure	0	3
**Abnosi et al., 2015**	Exposure	5	0
	No Exposure	0	5
**He et al., 2019**	Exposure	3	0
	No Exposure	0	3
**Liao et al., 2017**	Exposure	20	0
	No Exposure	0	20
**Liu et al., 2020**	Exposure	3	0
	No Exposure	0	3
**Cumulative**	Exposure	98	1
	No Exposure	1	95

Bone Marrow Adiposity

		**Outcome**	**No Outcome**
**Zhao et al., 2014**	Exposure	3	0
	No Exposure	0	3
**Beier et al., 2015**	Exposure	3	0
	No Exposure	0	3
**Luo et al., 2021**	Exposure	3	0
	No Exposure	0	3
**Abnosi et al., 2015**	Exposure	5	0
	No Exposure	0	5
**Cumulative**	Exposure	14	1
	No Exposure	1	14

Epigenetic Changes and miRNA Dysregulation

		**Outcome**	**No Outcome**
**Zhao et al., 2014**	Exposure	3	0
	No Exposure	0	3
**Chen et al., 2022**	Exposure	10	0
	No Exposure	0	10
**Rafiei et al., 2018**	Exposure	8	0
	No Exposure	0	8
**Wang et al., 2022a**	Exposure	12	0
	No Exposure	0	12
**Wu et al., 2014**	Exposure	3	0
	No Exposure	0	3
**Wu et al., 2019**	Exposure	3	0

	No Exposure	0	3
Ma et al., 2021a	Exposure	3	0
	No Exposure	0	3
Li et al., 2021	Exposure	3	0
	No Exposure	0	3
Cumulative	Exposure	45	1
	No Exposure	1	45

Enhanced Osteoclast Activity

		Outcome	No Outcome
Liu et al., 2016	Exposure	3	0
	No Exposure	0	3
Wu et al., 2014	Exposure	3	0
	No Exposure	0	3
Tomaszewska et al., 2016	Exposure	12	0
	No Exposure	0	12
Chen et al., 2022	Exposure	10	0
	No Exposure	0	10
Tong et al., 2023	Exposure	3	0
	No Exposure	0	3
Song et al., 2023	Exposure	6	0
	No Exposure	0	6
Wan et al., 2023	Exposure	22.11	45.89
	No Exposure	14.35	52.65
Torres-Rodriguez et al., 2022	Exposure	10	0
	No Exposure	0	10
Luo et al., 2021	Exposure	3	0
	No Exposure	0	3
Ma et al., 2021a	Exposure	3	0
	No Exposure	0	3
Ma et al., 2021b	Exposure	3	0
	No Exposure	0	3
Brzoska et al., 2016	Exposure	7	0
	No Exposure	0	7
Duranova et al., 2014	Exposure	10	0
	No Exposure	0	10
Liu et al., 2020	Exposure	3	0
	No Exposure	0	3
Cumulative	Exposure	108.11	45.89

	No Exposure	14.35	121.65

Altered Bone Microarchitecture

		Outcome	No Outcome
Liu et al., 2016	Exposure	3	0
	No Exposure	0	3
Chen et al., 2022	Exposure	10	0
	No Exposure	0	10
Wang et al., 2022	Exposure	12	0
	No Exposure	0	12
Wang et al., 2022a	Exposure	12	0
	No Exposure	0	12
Zhao et al., 2023	Exposure	3	0
	No Exposure	0	3
Ma et al., 2021a	Exposure	3	0
	No Exposure	0	3
Ma et al., 2021b	Exposure	3	0
	No Exposure	0	3
Lv et al., 2019	Exposure	8	0
	No Exposure	0	8
Liu et al., 2019	Exposure	3	0
	No Exposure	0	3
He et al., 2019	Exposure	3	0
	No Exposure	0	3
Liu et al., 2020	Exposure	3	0
	No Exposure	0	3
Cumulative	Exposure	63	1
	No Exposure	1	63
